# Exploring the immune-inflammatory mechanism of Maxing Shigan Decoction in treating influenza virus A-induced pneumonia based on an integrated strategy of single-cell transcriptomics and systems biology

**DOI:** 10.1186/s40001-024-01777-9

**Published:** 2024-04-15

**Authors:** Shiying Zhang, Bei Li, Liuting Zeng, Kailin Yang, Junyao Jiang, Fangguo Lu, Ling Li, Weiqing Li

**Affiliations:** 1 Shenzhen Traditional Chinese Medicine Hospital, Shenzhen, China; 2 Hunan University of Chinese Medicine, Changsha, Hunan China; 3 The Fourth Clinical Medical College of Guangzhou University of Chinese Medicine, Shenzhen, China; 4 Shenzhen Luohu People’s Hospital, Shenzhen, China; 5 The Third Affiliated Hospital of Shenzhen University, Shenzhen, China; 6 School of Life Science, Westlake University, Hangzhou, China

**Keywords:** Maxing Shigan Decoction, Influenza virus, Influenza A, Transcriptomics, Metabonomics, Microbiology

## Abstract

**Background:**

Influenza is an acute respiratory infection caused by influenza virus. Maxing Shigan Decoction (MXSGD) is a commonly used traditional Chinese medicine prescription for the prevention and treatment of influenza. However, its mechanism remains unclear.

**Method:**

The mice model of influenza A virus pneumonia was established by nasal inoculation. After 3 days of intervention, the lung index was calculated, and the pathological changes of lung tissue were detected by HE staining. Firstly, transcriptomics technology was used to analyze the differential genes and important pathways in mouse lung tissue regulated by MXSGD. Then, real-time fluorescent quantitative PCR (RT-PCR) was used to verify the changes in mRNA expression in lung tissues. Finally, intestinal microbiome and intestinal metabolomics were performed to explore the effect of MXSGD on gut microbiota.

**Results:**

The lung inflammatory cell infiltration in the MXSGD group was significantly reduced (*p* < 0.05). The results of bioinformatics analysis for transcriptomics results show that these genes are mainly involved in inflammatory factors and inflammation-related signal pathways mediated inflammation biological modules, etc. Intestinal microbiome showed that the intestinal flora *Actinobacteriota* level and *Desulfobacterota* level increased in MXSGD group, while *Planctomycetota* in MXSGD group decreased. Metabolites were mainly involved in primary bile acid biosynthesis, thiamine metabolism, etc. This suggests that MXSGD has a microbial–gut–lung axis regulation effect on mice with influenza A virus pneumonia.

**Conclusion:**

MXSGD may play an anti-inflammatory and immunoregulatory role by regulating intestinal microbiome and intestinal metabolic small molecules, and ultimately play a role in the treatment of influenza A virus pneumonia.

**Supplementary Information:**

The online version contains supplementary material available at 10.1186/s40001-024-01777-9.

## Introduction

Influenza is an acute respiratory infection caused by influenza virus [1, 2]. Influenza A virus is the main pathogen causing human and animal infections. The clinical manifestations of influenza are diverse. Common symptoms include fever, cough, headache, fatigue, and are often accompanied by gastrointestinal symptoms such as nausea, vomiting, diarrhea, and abdominal pain. Studies have reported that the incidence of gastrointestinal symptoms in patients with influenza is 30.9% [1, 2]. Influenza virus infection causes intestinal microbiome imbalance and mucosal local immune dysfunction may be an important reason why influenza is prone to gastrointestinal symptoms [3, 4]. The intestinal microbiome gradually colonizes in the digestive tract from the early stages of life, forming a stable intestinal microecosystem [5]. Intestinal microbiome and its metabolites are closely related to the host’s energy metabolism, immune homeostasis, and health status [5]. Studies showed that changes in the types and proportions of intestinal microbiome may have a positive or negative effect on the outcome of influenza virus infection, which is inseparable from the influence of intestinal microbiome and its metabolites on the immune function of the host [6]. It was found that after influenza virus infection, the structural composition of the intestinal microbiome changes, and intestinal epithelial cells can release excessive chemokines and pro-inflammatory cytokines [7, 8]. The interaction between the intestinal microbiome and local inflammatory factors in the mucosa will affect the course of influenza [7, 8]. Wang et al. found that influenza virus infection can promote pulmonary CCR9+CD4+ T cells to enter the intestinal tract and secrete interferon-γ (IFN-γ) to interfere with the homeostasis of the intestinal microbiota and cause imbalance of the intestinal microbiome [7]. The disordered intestinal microbiome can promote the secretion of interleukin-15 (IL-15) in small intestinal epithelial cells, induce Th17 cell polarization, and promote the production of IL-17 and other cytokines, thereby aggravating intestinal tissue damage. Li et al. [8] found that influenza virus infection can promote the growth of Enterobacteriaceae bacteria in the intestinal phylum Proteobacteria, which destroys the mucosal barrier function and induces intestinal epithelial cells to overexpress IL-22, IFN-α, IL-17A and other pro-inflammatory cytokines, causing inflammation and intestinal tissue damage. Inflammatory chemokines are important inflammatory factors that cause influenza immune pathological damage. They can strongly chemoattract inflammatory cells to accumulate in the lesion, and are positively correlated with the degree of inflammatory damage caused by influenza virus infection [9–11]. Studies have found that *Escherichia coli* or *Klebsiella pneumoniae* can promote the expression of inflammatory chemokines, thereby aggravating the degree of inflammatory damage [12]. Therefore, repairing the imbalance of the intestinal microecology and improving the local microenvironment of the mucosa are of great significance for the prevention and treatment of influenza.

**Fig. 1:**
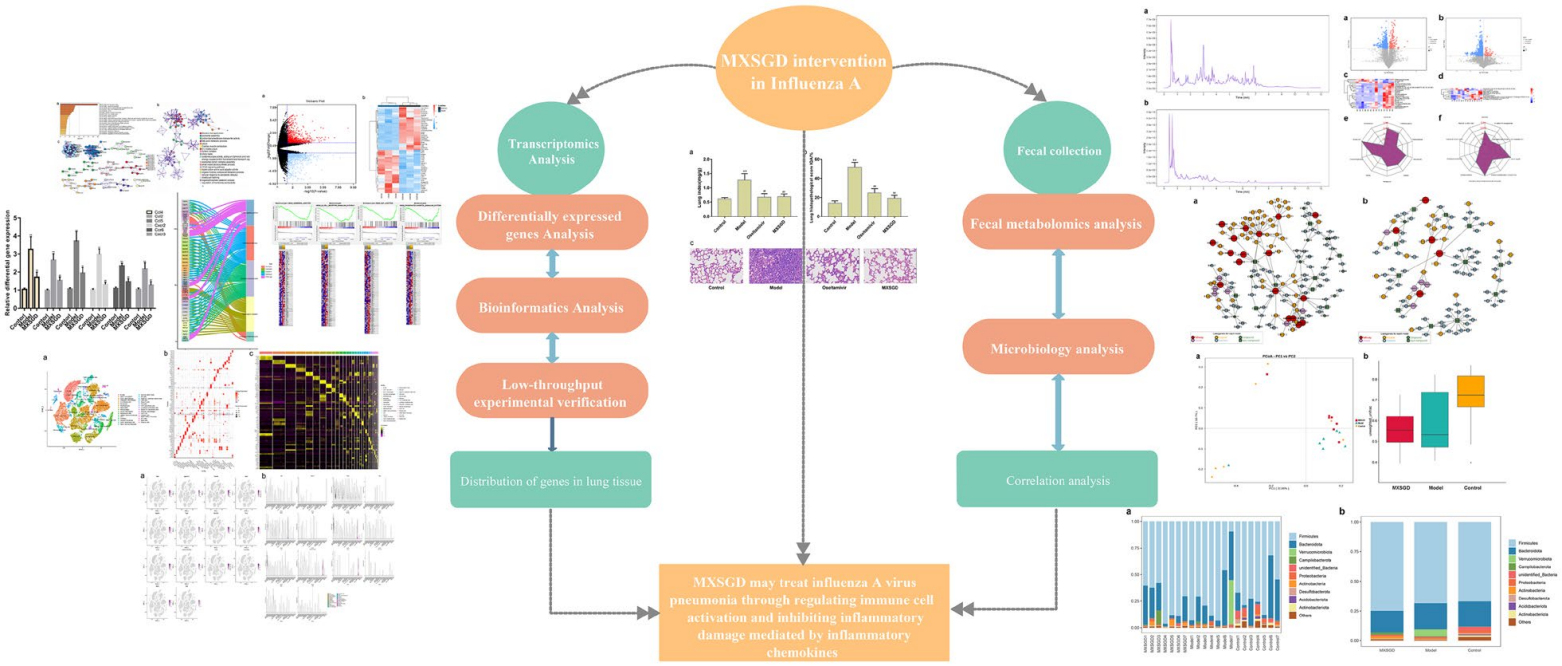
The research processes

Maxing Shigan Decoction (MXSGD), derived from Zhang Zhongjing’s Treatise on Febrile Diseases in the Han Dynasty, is a commonly used traditional Chinese medicine (TCM) prescription for the prevention and treatment of influenza. It is used to treat the syndrome of exogenous wind evil and heat evil blocking lung (namely, respiratory diseases such as upper respiratory tract infections and lung infections) [13]. In TCM, MXSGD is a classic prescription for cough and asthma, which has the effects of relieving heat and asthma, opening the nose, relieving skin itching, and benefiting the anorectum [14]. In addition to heat, MXSGD is also widely used in patients with anal fistula, hemorrhoids, and anal fissure, who are manifested as urgent defecation, strong body, or accompanied by cough and itchy skin [15]. Fan Wenfu, a famous modern physician, also used MXSGD to treat diarrhea of large intestine caused by lung heat, suggesting that MXSGD may be related to the intestinal microbiome [16]. Our previous research found that the prescription has the effects of interfering with virus adsorption, inhibiting virus proliferation, inhibiting the release of chemokines and inflammatory mediators [17, 18]. To further study the effects of the intestinal microbiome of mice with influenza A pneumonia and the mechanism of MXSGD intervention, this study used influenza virus infection in BALB/c mice as a model to explore the influence of influenza virus on the transcriptomics, intestinal microbiome and metabolomics of mice and the intervention effect of MXSGD, which further reveals the possible mechanism of MXSGD to prevent and treat influenza, and provides theoretical support and experimental data for expanding its clinical application. The research process is shown in Fig. 1.

## Materials and methods

### Experimental materials

#### Experimental animal

Eighty (80) Specified pathogen free (SPF) BALB/c mice (female:male = 1:1), body weight (20 ± 2) g, were purchased from Hunan Slack Jingda Experimental Animal Co., Ltd. Animal production license number: SCXK (xiang) 2016-0002, experimental unit use license number: SYXK (xiang) 2015-003. Mice were housed in the SPF animal room of the Experimental Animal Center of Hunan University of Chinese Medicine, with a temperature of (22 ± 2) °C, a relative humidity of 60% ± 10%, and a 12 h/12 h light–dark cycle. Mice had free access to food and water. The treatment of mice during the experiment has been approved by the Animal Ethics Committee of the Medical Innovation Experimental Center of Hunan University of Chinese Medicine.

#### Experimental drug

MXSGD is composed of *Ephedra herba* [*Ephedraceae*; Dried herbaceous stems of *Ephedra sinica* Stapf, *Ephedra intermedia* Schrenk et C.A.Mey. or *Ephedra equisetina* Bge.; (Chinese name: Ma Huang, MH)], *Armeniacae Semen Amarum* [*Rosaceae*; Dried mature seeds of *Prunus armeniaca* L.var.ansu Maxim., *Prunus sibirica* L., *Prunus mandshurica* (Maxim.) Koehne or *Prunus armeniaca* L.; (Chinese name: Ku Xing Ren, KXR)], *Glycyrrhizae Radix* et Rhizoma [*Fabaceae*; Dried roots and rhizomes of *Glycyrrhiza uralensis* Fisch., *Glycyrrhiza inflata* Bat. or *Glycyrrhiza glabra* L.; (Chinese name: Gan Cao, GC)] and Gypsum Fibrosum [Sulfate minerals; The main ingredient is hydrated calcium sulfate (CaSO_4_·2H_2_O); (Chinese name: Shi Gao, SG)]. MH (Lot number: 1411200312) 9 g, KXR (Lot number: 1505130152) 9 g, GC (Lot number: 20150901) 6 g and SG (Lot number: 1405220632) 18 g were purchased from the Traditional Chinese Medicine Pharmacy of the First Affiliated Hospital of Hunan University of Chinese Medicine. MH, KXR, GC and SG were identified by Associate Professor Dai Bing of Hunan University of Chinese Medicine. Oseltamivir phosphate capsules (Lot number: M1036; Produced by Roche S.p.A. in Italy, subpackaged by Shanghai Roche Pharmaceutical Co., Ltd.).

Preparation of MXSGD: MH 9 g, KXR 9 g, GC 6 g and SG 18 g were added, and then 1680 mL of distilled water (10 times the total amount of medicinal materials) was added to soak the medicinal materials for 30 min. After boiling, the medicinal materials were decocted for 30 min, and the first decoction liquid of MXSGD was filtered after the decoction was completed. During the second decocting, 1176 mL of distilled water was added to the medicinal residues (7 times of the total medicinal residues), boiled and then decocted for 20 min. After the decoction was completed, the second decoction liquid of MXSGD was filtered. Finally, the two decoctions of MXSGD were combined and concentrated to a concentration of 1 g crude drug/mL.

Preparation of oseltamivir: oseltamivir phosphate was fully dissolved in distilled water to make a suspension with a concentration of 1.08 mg/mL.

#### Instruments and reagents

Ephedrine hydrochloride (Lot number: 110736-201640), amygdalin (Lot number: 110820-201607), glycyrrhizic acid (Lot number: 110731-201616), glycyrrhetic acid (Lot number: 110723-201612) were provided by China National Institute for Food and Drug Control, and the purity was greater than 98%. Trizol Lysis Solution (Invitrogen Inc.). PrimeScript™ RT reagent kit with gDNA Eraser (RR047A), TB Green® Premix Ex Taq™ II (RR820A) (Takara Inc.). Phusion High-Fidelity PCR Master Mix with GC Buffer (Cat No. F532S; Thermo Scientific), TruSeq DNA PCR-Free Sample Preparation Kit (Cat No. FC-121-3003; illumina), Magnetic Bead Method Soil and Fecal Genomic DNA Extraction Kit (DP712) (Cat No. DP712-02; Tiangen Biochemical Technology (Beijing) Co., Ltd.), CTAB Solution (RNase free) (Cat No. ZT1008-1-500ml; Beijing Coollab Technology Co., Ltd.). Ultra High-Performance Liquid (Vanquish), High Resolution Mass Spectrometry (Q Exactive HFX), Centrifuge (Heraeus Fresco17) (Thermo Fisher Scientific).

#### Virus strain

A mouse lung-adapted strain of influenza A virus (A/PR/8/34), presented by the Virus Research Laboratory of Hunan Normal University, and preserved by the Pathogenic Biology Laboratory of Hunan University of Chinese Medicine. The virus was inoculated into the allantoic cavity of 10-day-old chicken embryos for culture and passage, and the hemagglutination titer was 1:640 for the experiment.

### Quality control of MXSGD by high performance liquid chromatography (HPLC)

#### Sample preparation

MXSGD sample solution was prepared according to “Experimental drug” section. Reference substance solution preparation: the reference substances of ephedrine hydrochloride, amygdalin, glycyrrhizic acid, and glycyrrhetinic acid were weighed in appropriate amounts, accurately measured, and 65% methanol was added to prepare a solution of ephedrine hydrochloride 0.192 mg/mL, amygdalin 0.216 mg/mL, glycyrrhizic acid 0.224 mg/mL, and glycyrrhetinic acid 0.100 mg/mL.

#### HPLC condition

Shim-Pack XR-ODS (4.6 nm × 250 nm, 5 μL) column, A: acetonitrile 0.1% phosphoric acid aqueous solution. B: mobile phase. The detection wavelength is 254 nm (0–50 min), the column temperature is 35 °C, the flow rate is 0.500 mL/min, and the injection volume is 10 μL. The results of HPLC were shown in Additional file 1: Fig. S1. The content of ephedrine hydrochloride was 0.2741 ± 0.0013 μg/mL, amygdalin 0.5501 ± 0.0031 μg/mL, glycyrrhizic acid 0.1702 ± 0.0021 μg/mL, and glycyrrhetinic acid 0.1669 ± 0.0011 μg/mL.

### Animal grouping and modeling

After 2 days of adaptive feeding, the mice were randomly assigned into normal control group, model control group, oseltamivir group and MXSGD group, with 20 mice in each group. Except for mice in the normal control group, mice in the other groups were nasally inoculated with 50 units of LD50 (50 LD50) influenza virus solution 0.05 mL to establish an influenza virus infection model [19]. The mice in the normal control group were inoculated with 0.05 mL of 0.9% saline in the same way.

### Animal intervention

The doses of MXSGD and oseltamivir were converted from clinical equivalent doses: the dose of Oseltamivir was 21.50 mg/(kg d); the dose of MXSGD was 2.8 g/(kg d). The reason why the dose of MXSGD is set to 2.8 g/(kg d) is that we have compared various doses of MXSGD in our previous studies [20–23], and found that 2.8 g/(kg d) has good curative effect and is representative. Therefore, despite the limitations of a single dose, MXSGD 2.8 g/(kg d) was selected as the representative dose for this study of transcriptomics, intestinal microbiome, and intestinal metabolomics.

Treatment begins 24 h after virus inoculation. Rats in the oseltamivir group were intragastrically administered oseltamivir 21.50 mg/(kg d). Rats in the MXSGD group were intragastrically administered with MXSGD 2.8 g/(kg d). Rats in the normal control group and the model control group were intragastrically administered with normal saline. The treatment was once a day for 3 days.

### Specimen collection and index testing

The mice were fasted after the last administration. The body weight of the mice was measured according to the routine, and the lungs were weighed to calculate the lung index. Lung index (mg/g) = lung mass (mg)/body mass (g).

After the mouse lung tissue was fixed with 4% paraformaldehyde for 1 week, hematoxylin–eosin (HE) staining was performed, and the pathological changes of the mouse lung tissue were observed under an optical microscope. Lung tissues were scored pathologically. Each group selected 6 slices, each with 4 fields of view (400×), and counted damaged alveoli [alveoli containing red blood cells or white blood cells > 2] as the index of quantitative assessment (IQA).

### Transcriptomics analysis

#### Total RNA extraction, cDNA library construction, and Illumina sequencing

The conventional Trizol method was used to extract total RNA from the lungs of each group of mice, and the residual DNA was digested with DNase I (RNase-free). The completeness and quality of RNA was checked by the Agilent 2100 Bioanalyzer. After enrichment, interruption, cDNA synthesis, end repair, PCR amplification and other processes, a sequencing library is prepared for Illumina sequencing (Shanghai Baiqu Biomedical Technology Co., Ltd.).

#### RNA-Seq quality assessment and sequence alignment

Quality control and filtering of raw reads obtained by the sequencing platform. Perform Q20, Q30, and GC content calculations on clean data, and select those with an error rate of less than 1% for subsequent transcriptome analysis, and more stringent filtering to obtain High quality clean reads. The HISAT2 software was used to quickly and accurately compare Clean Reads with the reference genome, and the gene expression levels between samples were analyzed by Pearson correlation test.

#### Differential expression analysis

The feature Counts tool in the subread software was used to quantitatively analyze the gene expression level of each sample, and the sequence depth and gene length were corrected successively using FPKM (Fragments Per Kilobase per Million). DESeq2 package (1.20.0) was used to analyze differentially expressed genes [24], and *p* < 0.05 and |log2 foldchange (FC)|  > 1 were used as the threshold for significant differential expression.

#### Bioinformatics analysis

Gene Ontology (GO, http://www.geneontology.org/, ver. 6.8) is the gene ontology database, including molecular function, biological processes and cellular component. Kyoto Encyclopedia of Genes and Genomes (KEGG, http://www.kegg.jp/, Last update 2022.01.01) is a database for systematic analysis of gene functions and genome information. The Metascape (http://metascape.org/gp/index.html#/main/step1) was used for enrichment analysis of differentially expressed genes [25].

#### Gene set enrichment analysis (GSEA)

Conventional enrichment analysis based on hypergeometric distribution relies on significantly up-regulated or down-regulated genes, and it is easy to miss some genes that are not significantly differentially expressed but have important biological significance. GSEA does not need to specify a clear threshold for differential genes. From the perspective of gene set enrichment analysis, it is easier to cover the impact of subtle but coordinated changes on biological pathways. This study uses the GSEA tool provided by Broad institute to perform GSEA (http://www.broadinstitute.org/gsea/index.jsp) on the KEGG dataset to supplement the results of the previous functional enrichment analysis [26].

#### RT-qPCR verification

The representative genes in the most enriched result (0002694: regulation of leukocyte activation) were verified by qRT-PCR. The transcriptomics results were verified by RT-qPCR using GAPDH as an internal reference. The 2-ΔΔCT method was used to determine the fold change of gene expression in the experimental group relative to the control group. The primer sequences are shown in Table 1. The RT-qPCR reaction system is 20 µL, and the reaction volume of each reagent is: TB Green® Premix Ex Taq™ II 10 µL, RNase Free H_2_O 6.4 µL, cDNA 2 µL, and the primers before and after PCR are 0.8 µL each. The reaction conditions are: 95 °C for 30 s; 95 °C for 5 s, 60 °C for 60 s; 40 cycles.

**Table 1:** Primers

	Upstream sequence	Downstream sequence
CXCR3	5′-GCTTGTCCTCCTTGTAGTTG-3′	5′-TAATGGTGTTGTCCTTGTTG-3′
CCR6	5′-AAAGTCCTCGCCTACACC-3′	5′-TTCTCATACACCACACATCCT-3′
CCL5	5′-GCTCCAATCTTGCAGTCGTG-3′	5′-GAGCAGCTGAGATGCCCATT-3′
CCL2	5′-GACCCCTAAATCTGAAGCTAATGC-3′	5′-AATTAAGGCATCACACTCCGACTC-3′
CCL4	5′-CCAGGCTTCTCAGCACCAAT-3′	5′-TTGGAGCAAAGACTGCTGCT-3′
Cxcr2	5′-CCACTCCCAGCATCGTAGAG-3′	5′-GTAAAGGGCGGGTCAGAACT-3′
GAPDH	5′-AGAAGGTGGTGAAGCAGGCATC-3′	5′-CGAAGGTGGAAGAGTGGGAGTTG-3′

#### Single cell sequencing data collection and processing

Single-cell sequencing data for lung tissue after influenza A virus infection were obtained from the GEO database (GSE202325). The single-cell dataset was preprocessed using the Seurat 4.0 package in R software, employing normalization techniques to correct for variations and eliminate low-quality cells [27]. Subsequently, an advanced algorithm called “FindVariableFeatures” was utilized to identify genes displaying high variability, facilitating subsequent analyses. To ensure equitable representation of individuals within the dataset, the “ScaleData” function was applied, determining a linear transformation that equalizes their influence in downstream principal component analysis (PCA). Following PCA, a dimensionality reduction method known as “tSNE” was employed, effectively mapping the data from the original high-dimensional space to a lower-dimensional space, while retaining local characteristics of the dataset. Finally, cell populations were identified and annotated using the “FindAllMarkers” function, leveraging the comprehensive CellMarker database (http://biocc.hrbmu.edu.cn/CellMarker/) to detect distinctive markers associated with each cell type.

### Intestinal microbiota and metabolite analysis

#### Fecal metabolite extraction

A 25 mg fecal sample was weighed, 500 μL of extraction solution (methanol: acetonitrile: water = 2:2:1 (V/V), containing isotope-labeled internal standard mixture) was added, and the mixture was vortexed for 30 s. The sample was then milled at 35 Hz for 4 min and ultrasonicated for 5 min (ice water bath). Then the sample was allowed to stand at − 40 °C for 1 h, and the sample was centrifuged at 4 °C at 12,000 rpm for 15 min. The supernatant of the sample was used for analysis, and all samples are taken and the same amount of supernatant was mixed to form a quality control (QC) sample for analysis.

#### UHPLC-QE-MS analysis

A Vanquish (Thermo Fisher Scientific) ultra-high performance liquid chromatograph was used for the analysis. The target compounds were chromatographed using Waters ACQUITY UPLC BEH Amide (2.1 mm × 100 mm, 1.7 μm) liquid chromatography column. The phase A of the liquid chromatography is the aqueous phase, containing 25 mmol/L ammonium acetate and 25 mmol/L ammonia, and the phase B is acetonitrile. The analysis was carried with elution gradient as follows: 0–0.5 min, 95%B; 0.5–7.0 min, 95–65% B; 7.0–8.0 min, 65–40% B; 8.0–9.0 min, 40% B; 9.0–9.1 min, 40–95% B; 9.1–12.0 min, 95% B. The flow rate was 0.5 mL/min. Mobile phase flow rate: 0.5 mL/min; column temperature: 30 °C, sample tray temperature: 4 °C, injection volume: 2 μL.

The Thermo Q Exactive HFX mass spectrometer was used for primary and secondary mass spectrometry data acquisition under the control of the control software. After the original data are converted into mzXML format by the ProteoWizard software, the R software was used to process peak identification, peak extraction, peak alignment and integration. Then an in-house MS2 database (BiotreeDB Ver. 2.1) was applied in metabolite annotation. The matrix data set is imported into SIMCA16.0 for Sample principal component analysis (PCA) and Orthogonal Partial Least Squares Method-Discriminant Analysis (OPLS-DA). *p* < 0.05 and (VIP) > 1 were used as the standard to screen the differential metabolites of the model/blank comparison group and the MXSGD/model comparison group.

#### 16S rRNA high-throughput bacterial population sequencing and analysis

The genomic DNA of the sample was extracted by the CTAB (hexadecyltrimethylammonium bromide) method, and then the purity and concentration of the DNA were detected by agarose gel electrophoresis. An appropriate amount of sample DNA is placed in a centrifuge tube, and the sample is diluted to 1 ng/μL with sterile water. The diluted genomic DNA was used as a template, and PCR was performed using specific primers with Barcode, New England Biolabs’ Phusion® High-Fidelity PCR Master Mix with GC Buffer, and high-efficiency high-fidelity enzymes. TruSeq® DNA PCR-Free Sample Preparation Kit was used for library construction. The constructed library was quantified by Qubit and Q-PCR. After the library was qualified, NovaSeq6000 was used for sequencing.

Uparse software (Uparse v7.0.1001, http://www.drive5.com/uparse/) was used to cluster all Effective Tags of all samples, clustering the sequences into Operational Taxonomic Units (OTUs). After the OTUs sequence was annotated for species, Qiime software (Version 1.9.1) was used to calculate Observed-otus, Chao1, Shannon, Simpson and Coverage, and R software was used to analyze the difference between groups of Alpha diversity index [28]. Then, Qiime software (Version 1.9.1) was used to calculate Unifrac distance, construct UPGMA sample clustering tree, and R software was utilized to analyze the differences between groups of Beta diversity index.

#### Correlation analysis of intestinal microbiota and metabolite

The cor.test function in R software was used for correlation analysis, and the calculation method was Spearman test. *p* < 0.05 indicated that there was a correlation between differential flora and differential metabolites. In the heat map, red indicates *r* > 0, which is a positive correlation; blue indicates *r* < 0, which is a negative correlation.

### Statistical analysis

The SPSS 21.0 statistical software is used for statistical analysis, and the measurement data are expressed as “Mean ± SD”, and a one-way analysis of variance is used. When the variance is uniform, the LSD test is used for pairwise comparison between groups; when the variance is not uniform, the Dunnett’s T3 test is used. *p* < 0.05 indicates that the difference is statistically significant.

## Results

### MXSGD effectively alleviates influenza A virus-induced lung inflammation in vivo

**Fig. 2:**
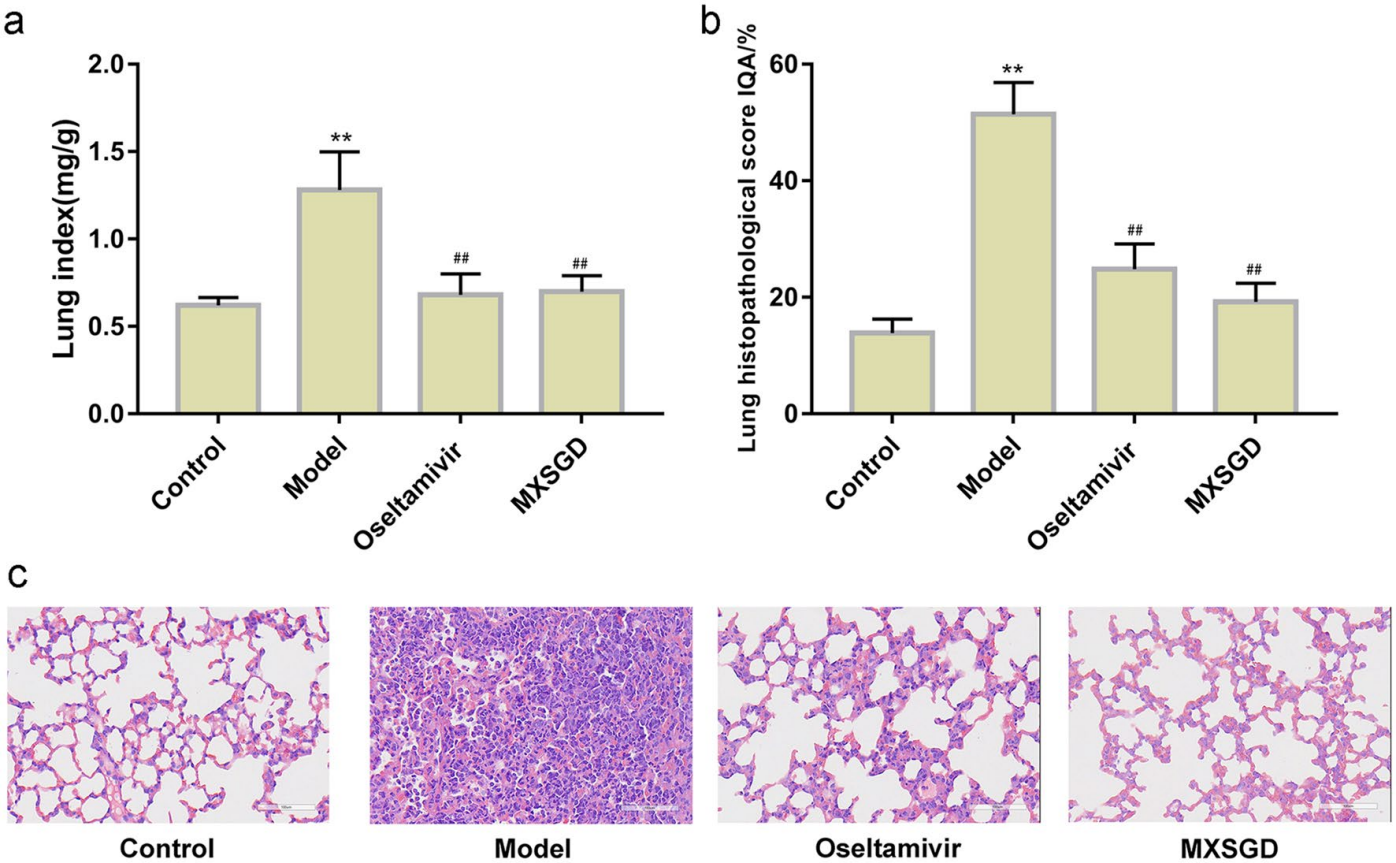
Effect of MXSGD on the lung index (**a** lung index; **b** lung histopathological score; **c** pathological change, HE staining, ×400. **Compared with control group, *p* < 0.05. ^##^Compared with model group, *p* < 0.05. *n* = 5)

The lung index reflects the edema of the lungs and indirectly reflects the level of lung inflammation. Compared with the normal control group, the lung index in model group was significantly increased (*p* < 0.05). Compared with the model control group, the lung index in MXSGD group and oseltamivir group was decreased (*p* < 0.05). There were no significant differences in the body mass and lung index of the mice between the MXSGD group and oseltamivir group (*p* > 0.05) (Fig. 2a).

The results of HE staining of lung tissue also revealed the role of MXSGD. In the control group, the alveolar, alveolar sac, alveolar duct, and alveolar septum were intact, with clear contours of the alveolar cavity, no secretions in the cavity, and no obvious inflammatory cell infiltration. In the model group, the alveolar structure of the mice was significantly destroyed, the alveoli collapsed or even disappeared, a large number of inflammatory cells such as lymphocytes, monocytes and neutrophils were seen in the alveolar cavity and lung interstitium; compared with the control group, the IQA value was significantly higher (*p* < 0.05). Compared with the model group, the degree of lung inflammation and injury in the oseltamivir group and MXSGD group were reduced. It is manifested as thickening of alveolar walls, a small amount of inflammatory cell infiltration in the lung interstitium, but the outline of the alveolar cavity is clear, the inflammatory cell infiltration in the cavity is lighter, and the IQA was significantly lower than that of the model group (*p* < 0.05) (Fig. 2b, c).

### Effects of MXSGD on transcriptomics

#### Differentially expressed genes

**Fig. 3:**
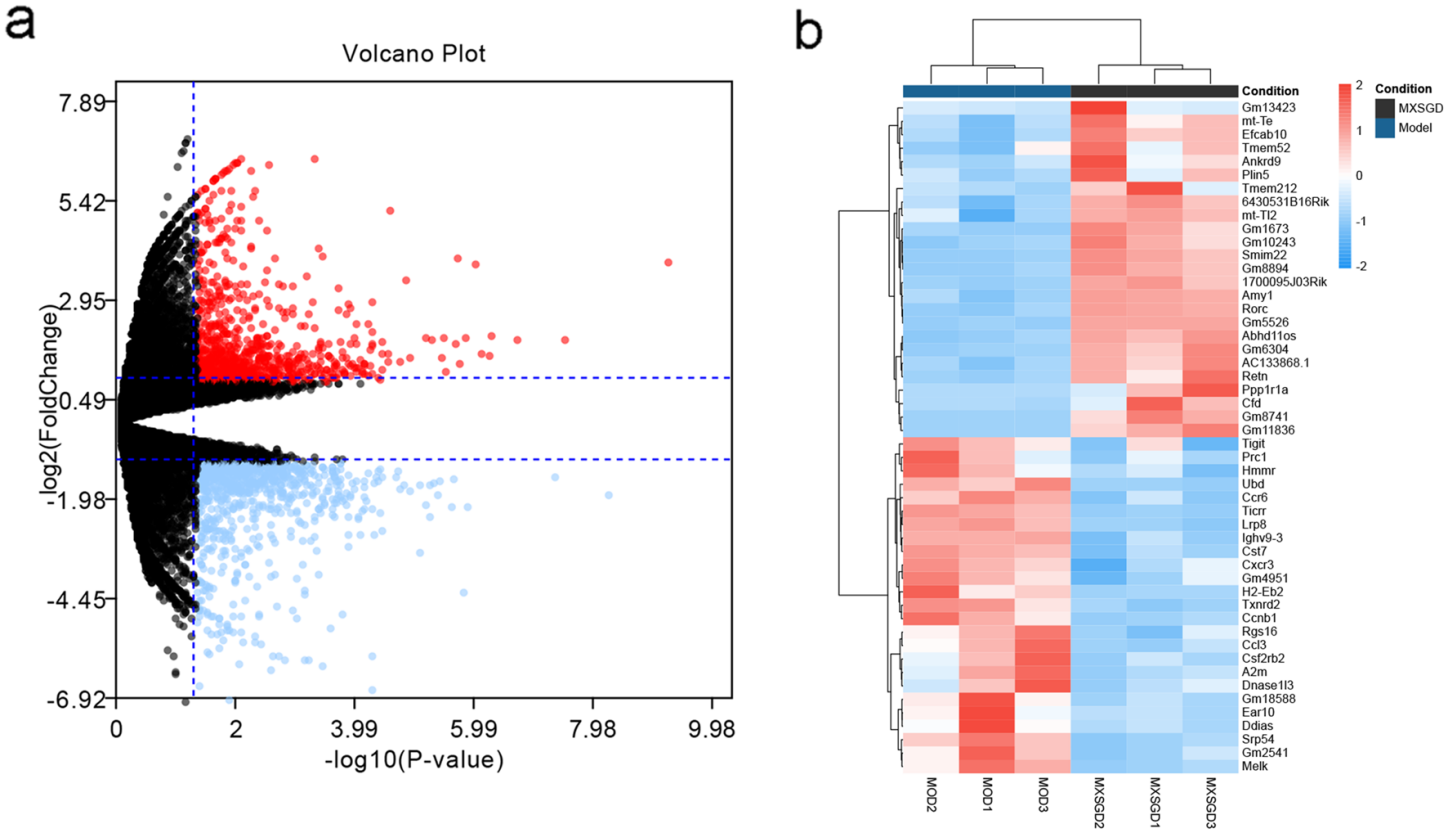
Differentially expressed gene (**a** volcano map; red represents up-regulated genes, blue represents down-regulated genes, and black represents genes with no differential expression. **b** heatmap of top 25 up-regulated and down-regulated differentially expressed gene.)

Comparing the gene expression values of MXSGD with the model group, there were a total of 1665 differentially expressed genes, including 807 down-regulated genes and 858 up-regulated genes. Among them, up-regulated genes include Cfd, Gm1673, Rorc, Smim22, Ndufa2, Zfp524, Gm10243, Wfdc2, Gm11836, Gm30025, Bcl2l1, Abhd11os, Gm19935, Efcab10, Gm5526; and down-regulated genes include Haus2, Gbp9, Ikzf3, Ighv9-3, Dennd4c, Eln, Gm10031, Coro1b, Cacna1d, Srp54, Nrcam, Vcl, Txnrd2, Nr4a3, Mat2a, Pom121. These genes were used for enrichment analysis to find the key pathways by which MXSGD intervenes in pneumonia (Fig. 3).

#### Enrichment analysis results of up-regulated gene

**Fig. 4:**
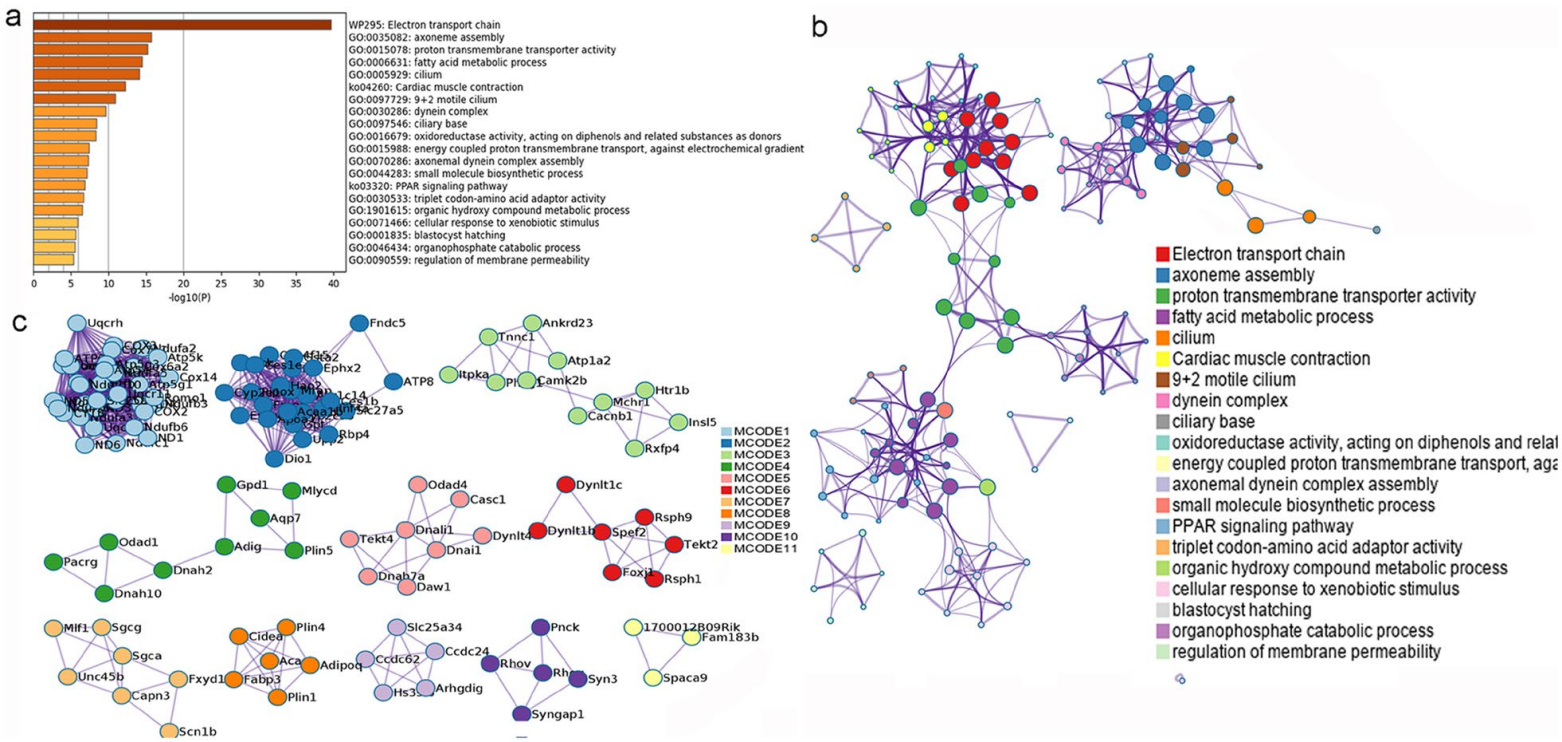
The enrichment analysis results of up-regulated genes (**a** bar graph colored by *p*-value [Fisher’s exact test]; **b** PPI network; **c** clusters)

The results showed that MXSGD may up-regulate some genes to regulate inflammatory factors and inflammation-related biological processes and signaling pathways, thereby improving lung inflammation. For example, the enrichment analysis results showed that MXSGD may regulate biological processes such as oxidative phosphorylation, ATP metabolic process, electron transport chain, cellular respiration, mitochondrial respiratory chain complex assembly, energy derivation by oxidation of organic compounds; cell components such as respirasome, inner mitochondrial membrane protein complex, mitochondrial respirasome; molecular function such as oxidoreduction-driven active transmembrane transporter activity, electron transfer activity, NADH dehydrogenase (ubiquinone) activity, NADH dehydrogenase (quinone) activity. The signaling pathway that MXSGD may regulated include Oxidative phosphorylation, Aminoacyl-tRNA biosynthesis, PPAR signaling pathway, Peroxisome, etc. (Fig. 4 and Additional file 2: Table S1).

#### Enrichment analysis results of down-regulated gene

**Fig. 5:**
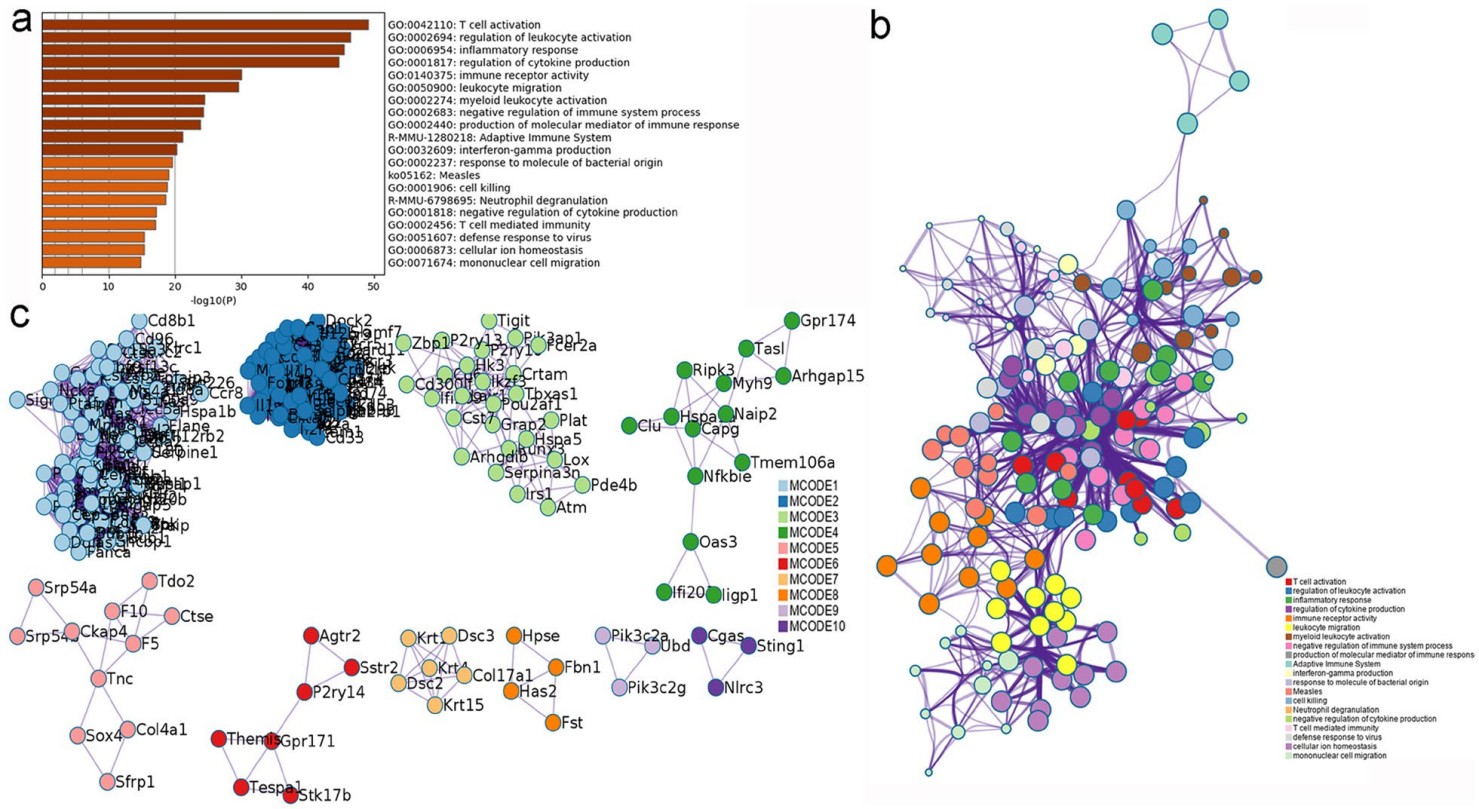
The enrichment analysis results of down-regulated genes (**a** bar graph colored by *p*-value; **b** PPI network, **c** clusters)

The results showed that MXSGD may down-regulate some genes to regulate inflammatory factors and inflammation-related biological processes and signaling pathways, thereby improving lung inflammation. For example, the enrichment analysis results showed that MXSGD may regulate biological processes such as regulation of leukocyte activation, regulation of cell activation, positive regulation of immune response, regulation of lymphocyte activation, positive regulation of leukocyte activation; cell components such as external side of plasma membrane, immunoglobulin complex, circulating, immunoglobulin complex; molecular function such as immune receptor activity, antigen binding, immunoglobulin receptor binding, cytokine receptor activity. The signaling pathway that MXSGD may regulated include cytokine–cytokine receptor interaction, Hematopoietic cell lineage, NOD-like receptor signaling pathway, T cell receptor signaling pathway, Th17 cell differentiation, Th1 and Th2 cell differentiation, Toll-like receptor signaling pathway, etc. (Additional file 3: Table S2 and Fig. 5).

#### Enrichment analysis results of all genes

**Fig. 6:**
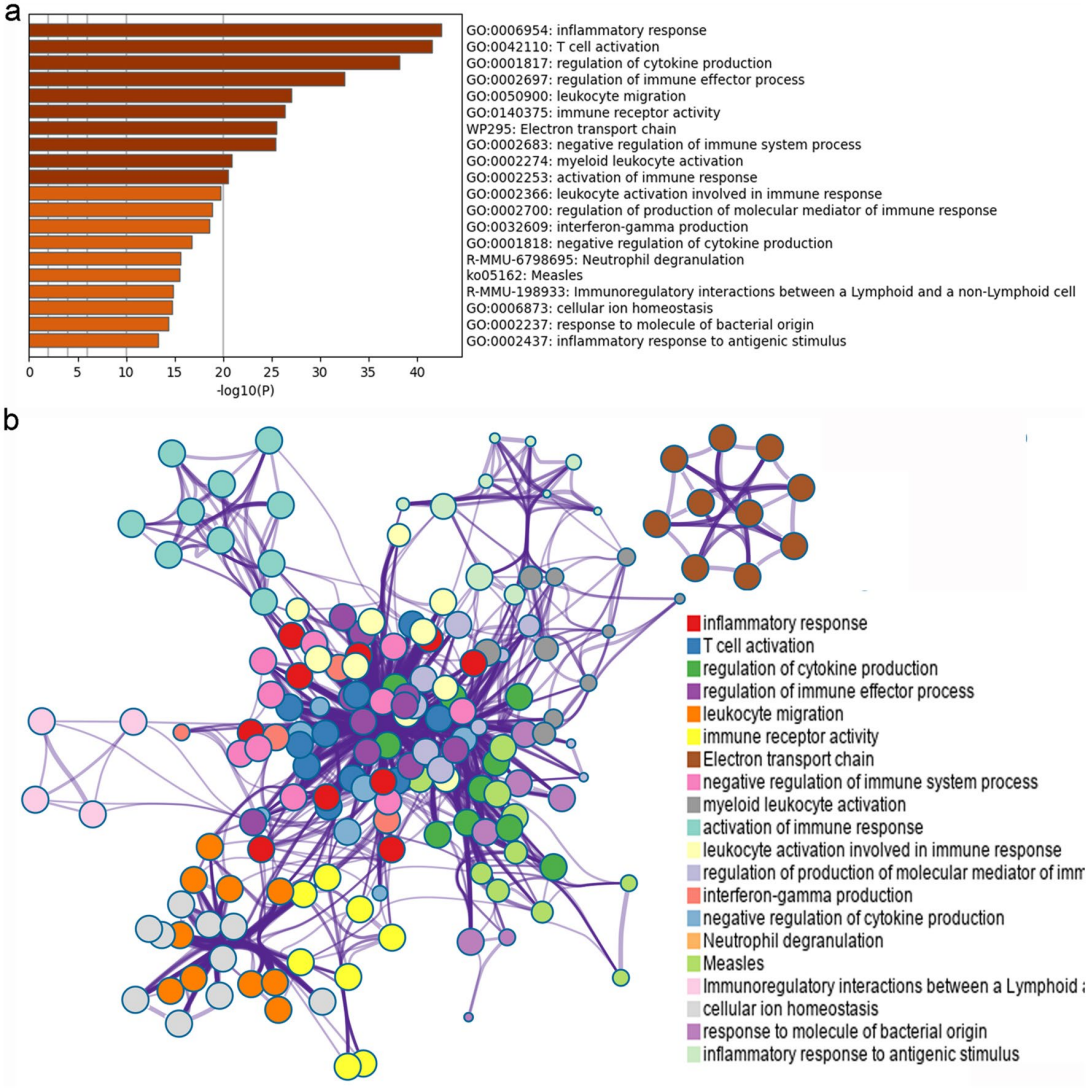
The enrichment analysis results of all genes (**a** bar graph colored by *P*-value; **b** PPI network)

Overall, the results showed that MXSGD may regulate those genes to regulate inflammatory factors and inflammation-related biological processes and signaling pathways, thereby improving lung inflammation. For example, the enrichment analysis results showed that MXSGD may regulate biological processes such as regulation of leukocyte activation, regulation of cell activation, regulation of lymphocyte activation, positive regulation of cell activation, positive regulation of leukocyte activation, positive regulation of immune response; cell components such as external side of plasma membrane, respirasome, respiratory chain complex, oxidoreductase complex; molecular function such as immune receptor activity, antigen binding, cytokine binding, cytokine receptor activity, oxidoreduction-driven active transmembrane transporter activity, immunoglobulin receptor binding. The signaling pathway that MXSGD may regulate include Parkinson’s disease, Cytokine–cytokine receptor interaction, Oxidative phosphorylation, Huntington’s disease, Hematopoietic cell lineage, Alzheimer’s disease, T cell receptor signaling pathway, etc. (Additional file 4: Table S3 and Fig. 6).

#### GSEA results

**Fig. 7:**
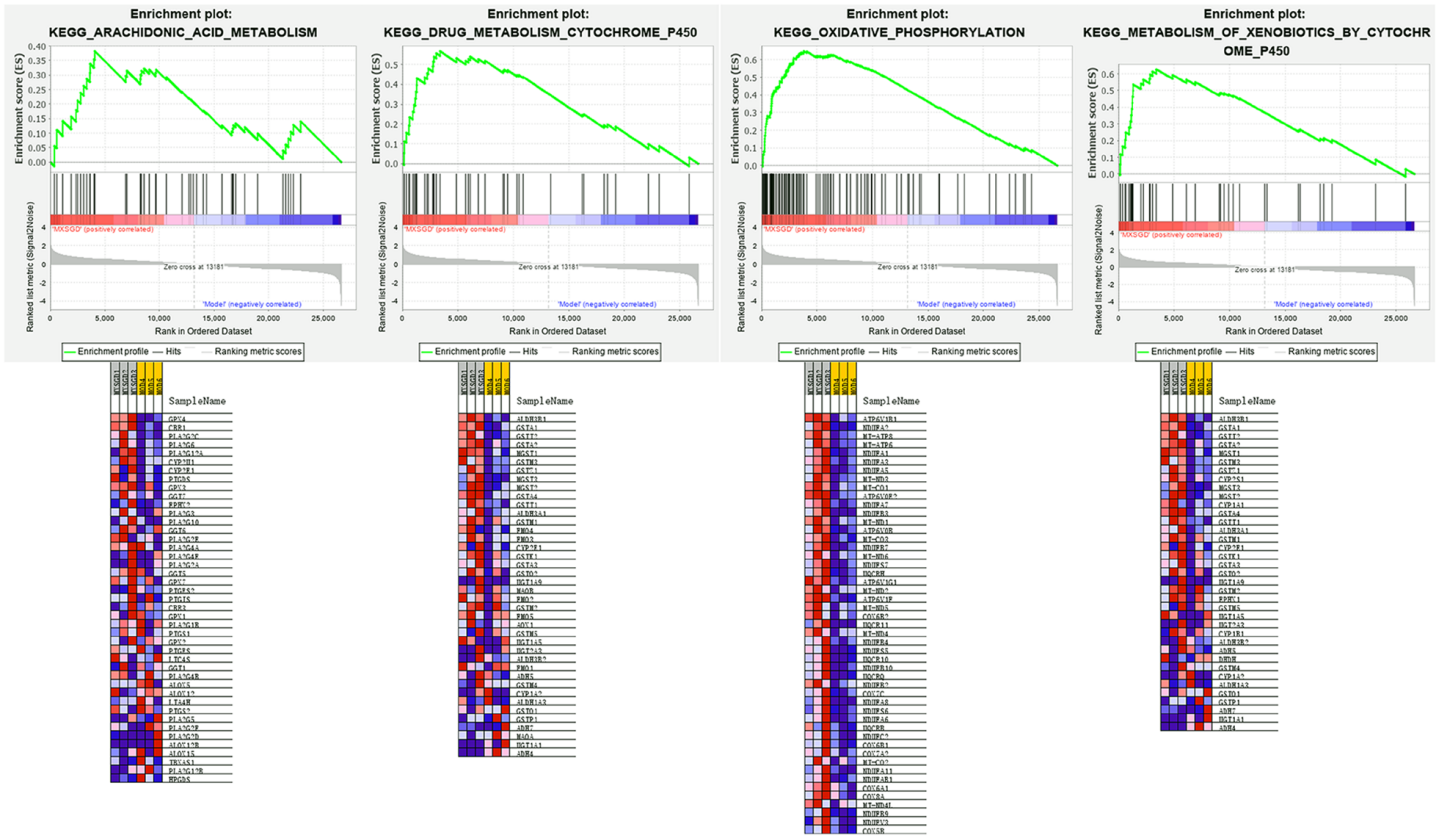
The pathways in up-regulated gene sets

**Fig. 8:**
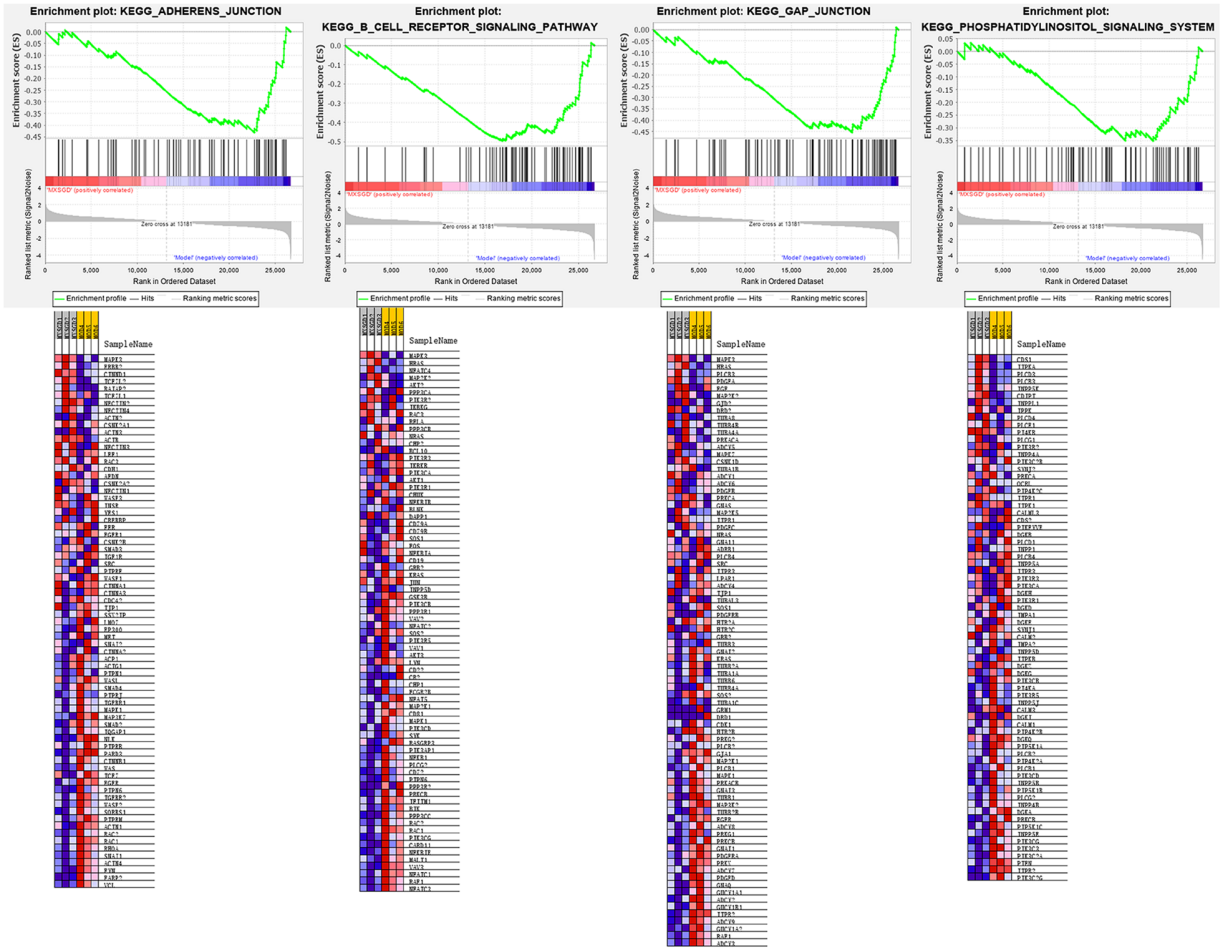
The pathways in down-regulated gene sets

The GSEA results showed that 49/169 gene sets were up-regulated in phenotype MXSGD, while 120/169 gene sets were down-regulated in phenotype MXSGD. The pathways in up-regulated gene sets include metabolism of xenobiotics by cytochrome p450, drug metabolism cytochrome p450, oxidative phosphorylation, arachidonic acid metabolism (Fig. 7). The pathways in down-regulated gene sets include gap junction, adherens junction, phosphatidylinositol signaling system, b cell receptor signaling pathway (Fig. 8).

#### Effect of MXSGD on the expression of CCL4, CCL2, CCL5, CXCR2, CCR6 and CXCR3 mRNA

**Fig. 9:**
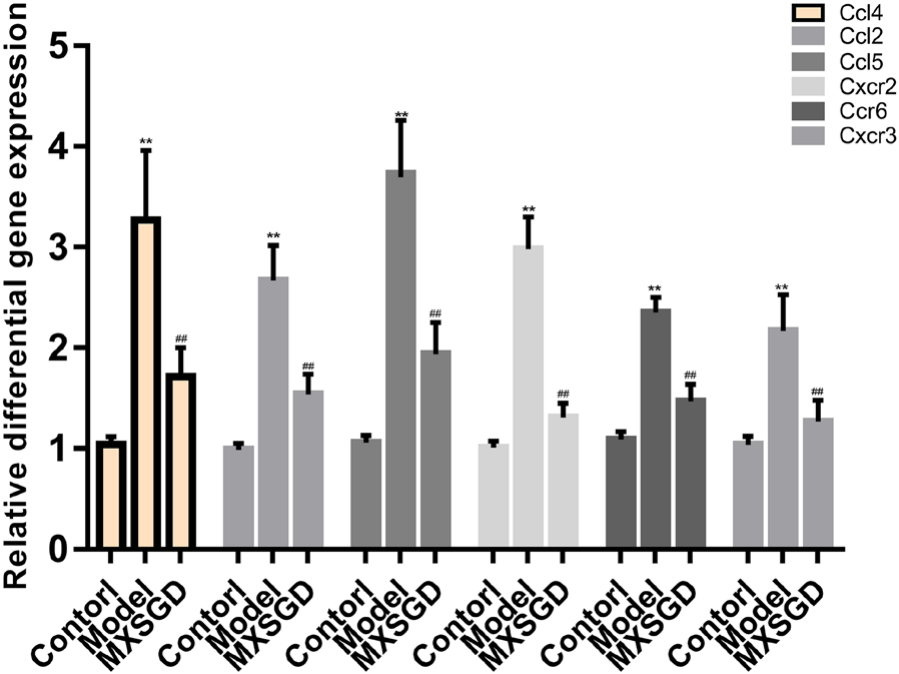
Effect of MXSGD on the expression of CCL4, CCL2, CCL5, CXCR2, CCR6 and CXCR3 mRNA (**compared with control group, *p* < 0.05; ^##^compared with model group, *p* < 0.05)

The central target CCL4, CCL2, CCL5, CXCR2, CCR6, CXCR3 of multiple inflammatory signaling pathways and immune signaling pathways in the transcriptome differential gene enrichment analysis in GO/KEGG bio-enrichment was used as the gene to verify the transcriptomic results. Compared with the blank control, CCL4, CCL2, CCL 5, CXCR2, CCR6, CXCR3 were up-regulated in the model group, and down-regulated after MXSGD intervention, which was consistent with the transcriptomics results (Fig. 9).

#### Transcriptomic gene expression distribution in lung tissue cells

**Fig. 10:**
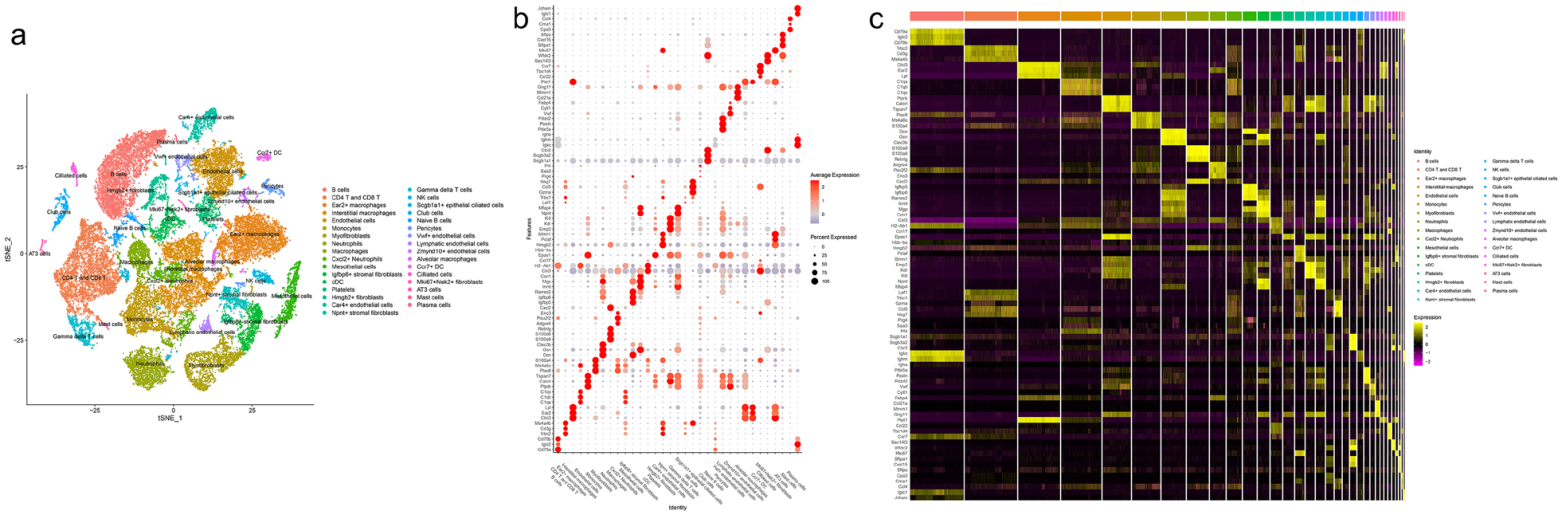
Cell cluster identification (**a** identified cell clusters of lung tissue; **b** average expression of top 5 genes of cell clusters; **c** expression of top 3 gene of cell clusters in single cells)

**Fig. 11:**
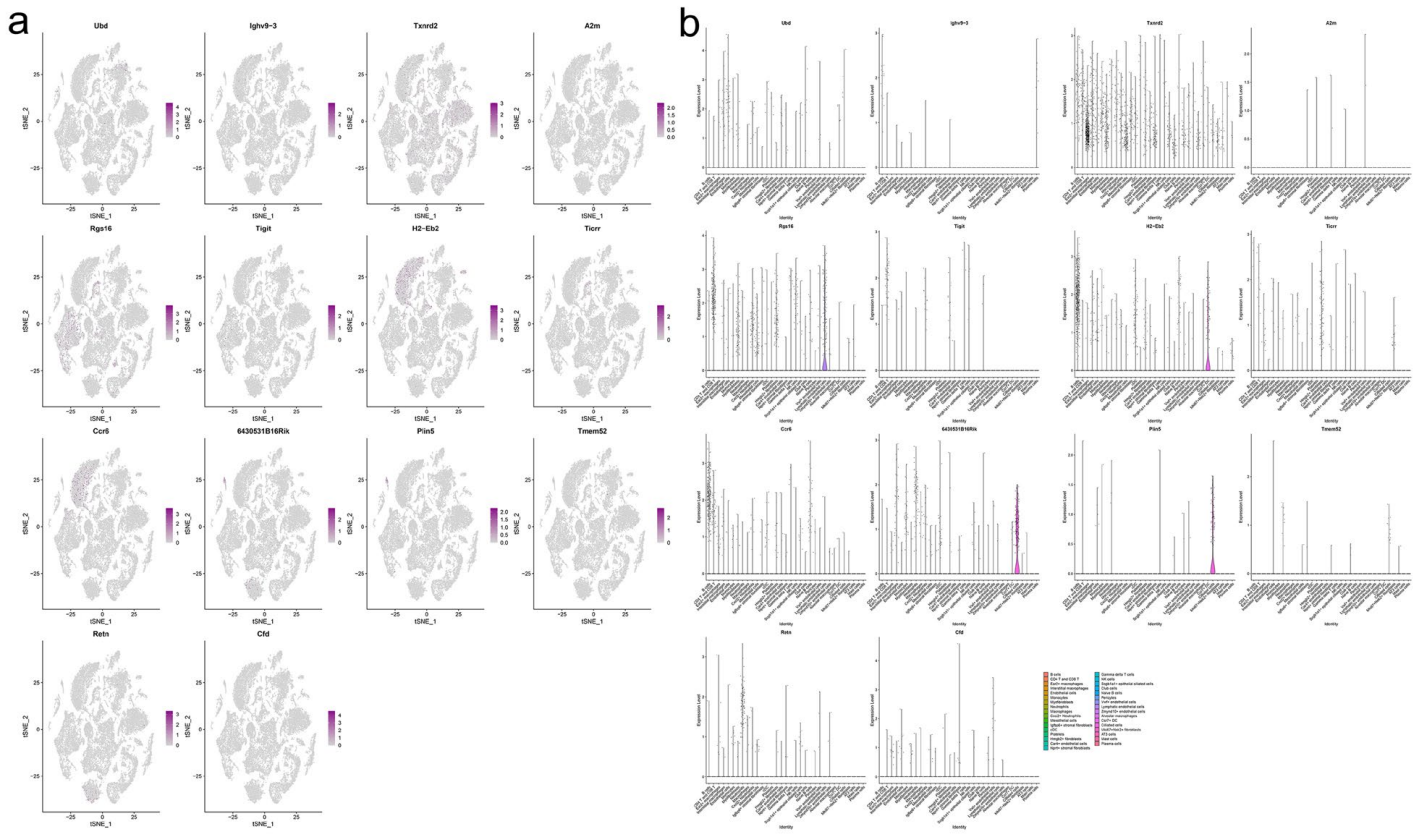
Distribution of the top 10 transcriptomic up- and down-regulated genes in lung tissue (**a** distribution of genes; **b** average expression of genes)

Single-cell sequencing data for lung tissue after influenza A virus infection were obtained from the GEO database (GSE202325). Cells with nFeature_RNA > 200 and < 2500, and percent.mt < 20% were retained for further analysis. Data after quality control were further processed to generate tSNE cluster plots. A total of 32 cell clusters were isolated and identified: B cells, CD4 T and CD8 T, Ear2+ macrophages, Interstitial macrophages, Endothelial cells, Monocytes, Myofibroblasts, Neutrophils, Macrophages, Cxcl2+ Neutrophils, Mesothelial cells, Igfbp6+ stromal fibroblasts, cDC, Platelets, Hmgb2+ fibroblasts, Car4+ endothelial cells, Npnt+  stromal fibroblasts, Gamma delta T cells, NK cells, Scgb1a1+ epithelial ciliated cells, Club cells, Naive B cells, Pericytes, Vwf+ endothelial cells, Lymphatic endothelial cells, Zmynd10+  endothelial cells, Alveolar macrophages, Ccr7+ DC, Cilliated cells, Mki67+Nek2+ fibroblasts, AT3 cells, Mast cells, Plasma cells (Fig. 10). Mapping the top 10 transcriptomic up- and down-regulated genes to lung tissue single-cell sequencing data, it was found that the genes in these subclusters had obvious distribution differences. For example, Txnrd2 was mainly distributed in Ear2+ macrophages; Rgs16 was mainly distributed in lymphatic endothelial cells; H2-Eb2 was mainly distributed in Ccr7+ DC and so on (Fig. 11).

### Effects of MXSGD on intestinal microbiota

**Fig. 12:**
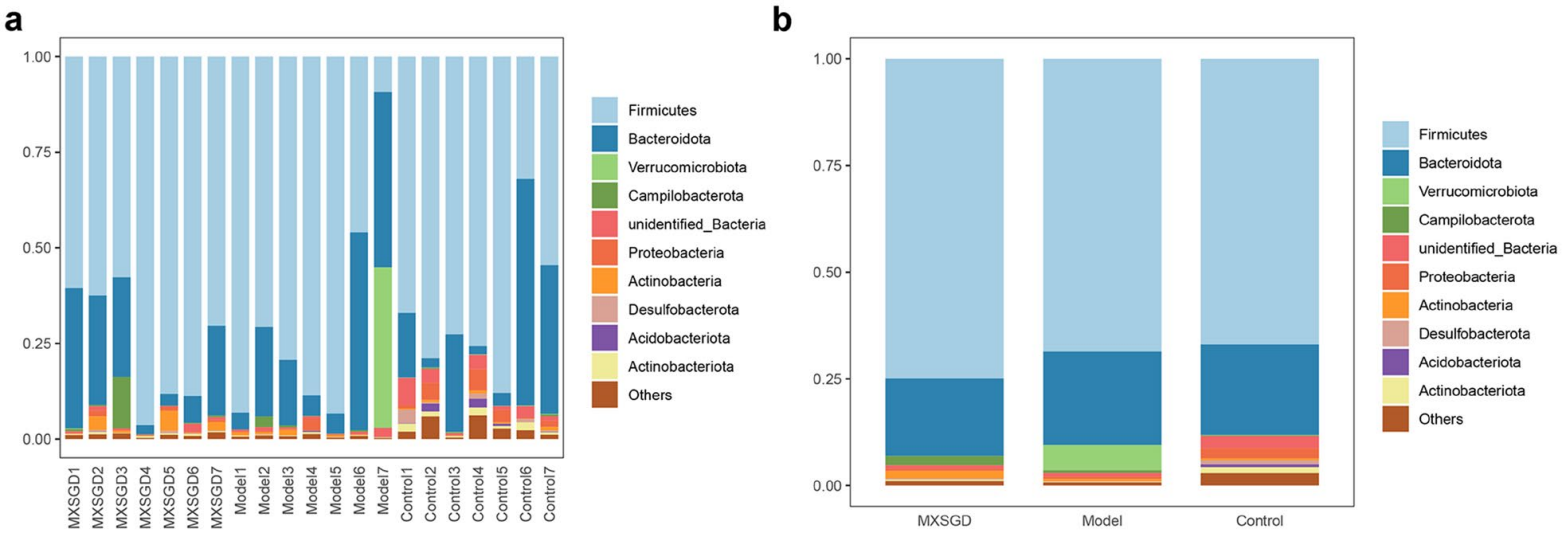
The distribution of microorganisms in the intestines of mice in Phylum level (**a** the composition of each sample; **b** the composition of each group. *Y* axis represents the proportion)

The composition of the intestinal microbiome of mice between the blank group, the model group and the MXSGD group was compared from the Phylum level of bacteria. The bacterial composition of the Phylum level is shown in Fig. 12, Fig. 12a is the percentage of bacterial composition of each sample, and Fig. 12b is the percentage of bacterial composition of each group. At the phylum level, the intestinal microbiome is mainly *Firmicutes*, *Bacteroidota*, *Verrucomicrobiota*, *Campilobacterota*, *unidentified_Bacteria*, *Proteobacteria*, *Actinobacteria*, *Desulfobacterota*, *Acidobacteriota*, *Actinobacteriota* (Table 2). In high abundance flora, compared with the control group, *Actinobacteriota* and *unidentified_Bacteria* in model was lower (*p* < 0.05); compared with model group, the intestinal microbiome *Actinobacteriota* level and *Desulfobacterota* level increased after MXSGD intervention. In addition, compared with control group, *Cyanobacteria*, *Gemmatimonadota*, *RCP2-54*, *Zixibacteria*, *Kapabacteria* in model group decreased (*p* < 0.05); compared with model group, *Planctomycetota* in MXSGD group decreased (*p* < 0.05) (Table 2).

**Table 2:** The distribution of microorganisms in the intestines of mice in Phylum level (%)

Phylum	Group		
	MXSGD	Model	Control
*Firmicutes*	74.9012	68.5926	66.9455
*Bacteroidota*	18.1925	21.8872	21.2303
*Verrucomicrobiota*	0.0203	5.9858	0.0836
*Campilobacterota*	2.1842	0.5805	0.1603
*unidentified_Bacteria*	0.7799	0.8328*	2.885
*Proteobacteria*	0.5142	0.5763	2.2962
*Actinobacteria*	1.7294	0.5067	0.4595
*Desulfobacterota*	0.2368#	0.0726	0.96
*Acidobacteriota*	0.0137	0.0484	0.7306
*Actinobacteriota*	0.3413#	0.2093*	1.3186
Others	1.0866	0.708	2.9304

*Compared with control group, *p* < 0.05, indicating a significant difference; ^#^compared with model group, *p* < 0.05, indicating a significant difference

**Fig. 13:**
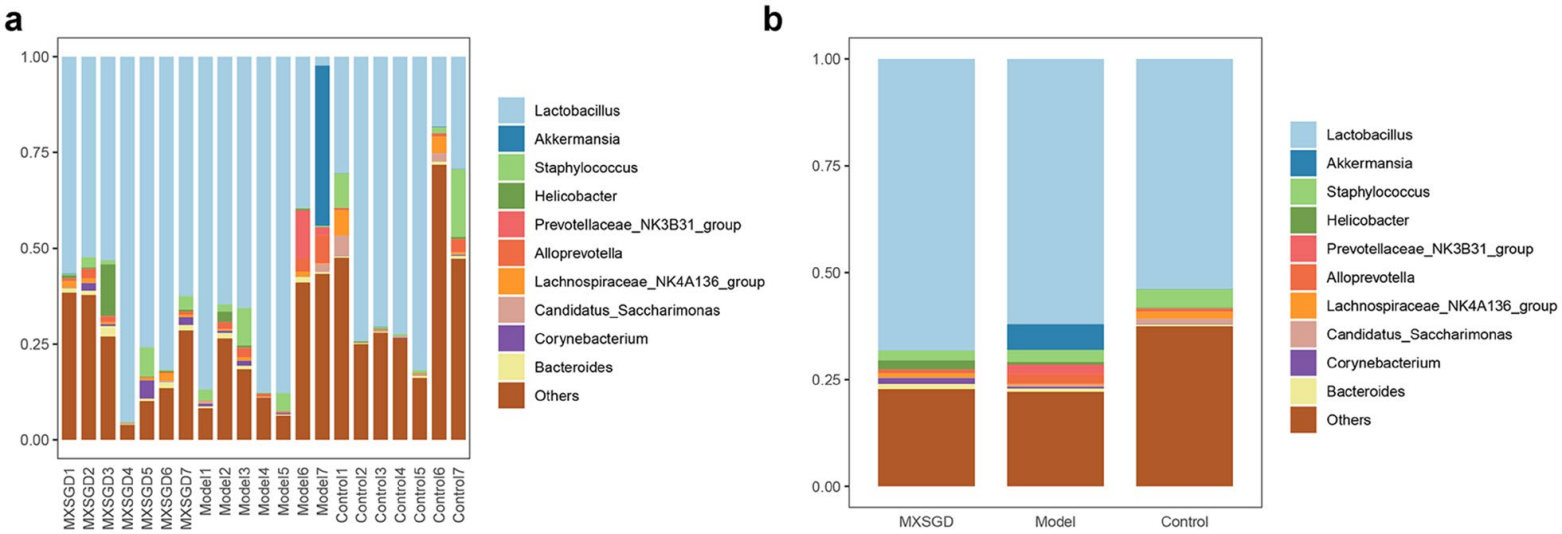
The distribution of microorganisms in the intestines of mice in Genus level (**a** the composition of each sample; **b** the composition of each group)

At the Genus level, the intestinal microbiome is mainly *Lactobacillus*, *Akkermansia*, *Staphylococcus*, *Helicobacter*, *Prevotellaceae_NK3B31_group*, *Alloprevotella*, *Lachnospiraceae_NK4A136_group*, *Candidatus_Saccharimonas*, *Corynebacterium*, *Bacteroides* (Table 3). The bacterial composition of the Phylum level is shown in Fig. 13, Fig. 13a is the percentage of bacterial composition of each sample, and Fig. 13b is the percentage of bacterial composition of each group. In high abundance flora, there was no significant difference in the intestinal microbiome at the level of Genus among the three groups (*p* > 0.05). In addition, compared with control group, *Desulfovibrio*, *Enterorhabdus*, *Bifidobacterium*, *Monoglobus*, *Gemella*, *Gaiella*, *Anaerostipes*, *RB41*, *Ellin6055*, *Bryobacter*, *[Eubacterium]_ventriosum_group*, *Ellin6067*, *Skermanella*, *Subgroup_10*, *Lysobacter, [Eubacterium]_brachy_group*, *Pseudomonas*, *Streptomyces*, *Paracoccus*, *unidentified_Gemmatimonadaceae*, *Adhaeribacter*, *Candidatus_Alysiosphaera*, *Stenotrophobacter*, *Aeromicrobium*, *Altererythrobacter*, *Ilumatobacter*, *Candidatus_Udaeobacter*, *Nordella*, *Nocardioides*, *Chryseolinea*, *Pedomicrobium*, *Nitrosospira*, *Bauldia*, *Flavitalea*, *Pseudochrobactrum*, *Edaphobaculum*, *Rubellimicrobium*, *Steroidobacter*, *Parviterribacter*, *Desulfobacca*, *Subsaxibacter*, *YC-ZSS-LKJ147*, *Agromyces*, *Caulobacter*, *Rhodococcus*, *Fluviicola*, *Bosea*, *Hyphomicrobium*, *Limibaculum*, *Flavihumibacter*, *Roseisolibacter*, *Pontibacter*, *Flavobacterium*, *Candidatus_Entotheonella*, *Vicinamibacter*, *unidentified_SAR324_clade(Marine_group_B)*, *Lacibacter* in model group decreased, while *unidentified_Chloroplas and Rikenellaceae_RC9_gut_group* increased (*p* < 0.05). Compared with model group, *Paenibacillus*, *Rikenellaceae_RC9_gut_group*, *Microvirga*, *Flavisolibacter*, *Pedomicrobium* in MXSGD group decreased, while *Enterorhabdus*, *Candidatus_Arthromitus*, *Anaerotruncus, Anaeroplasma*, *Alcaligenes, [Eubacterium]_brachy_group, Microbacterium*, *Negativibacillus*, *Oligella*, *Paracoccus*, *Tuzzerella* increased (*p* < 0.05).

**Table 3:** The distribution of microorganisms in the intestines of mice in Genus level (%)

Genus	Group		
	MXSGD	Model	Control
*Lactobacillus*	68.2035	62.0484	53.8542
*Akkermansia*	0.0203	5.9828	0.0606
*Staphylococcus*	2.26	2.8551	4.2842
*Helicobacter*	2.1842	0.567	0.1603
*Prevotellaceae_NK3B31_group*	0.0484	2.1504	0.069
*Alloprevotella*	0.8375	2.2842	0.6778
*Lachnospiraceae_NK4A136_group*	0.9928	0.4285	1.73
*Candidatus_Saccharimonas*	0.0812	0.3959	1.1735
*Corynebacterium*	1.3771	0.4243	0.1123
*Bacteroides*	1.2475	0.7312	0.4153
Others	22.7474	22.132	37.4628

### Effects of MXSGD on intestinal metabolite

#### Differential analysis of intestinal metabolites

**Fig. 14:**
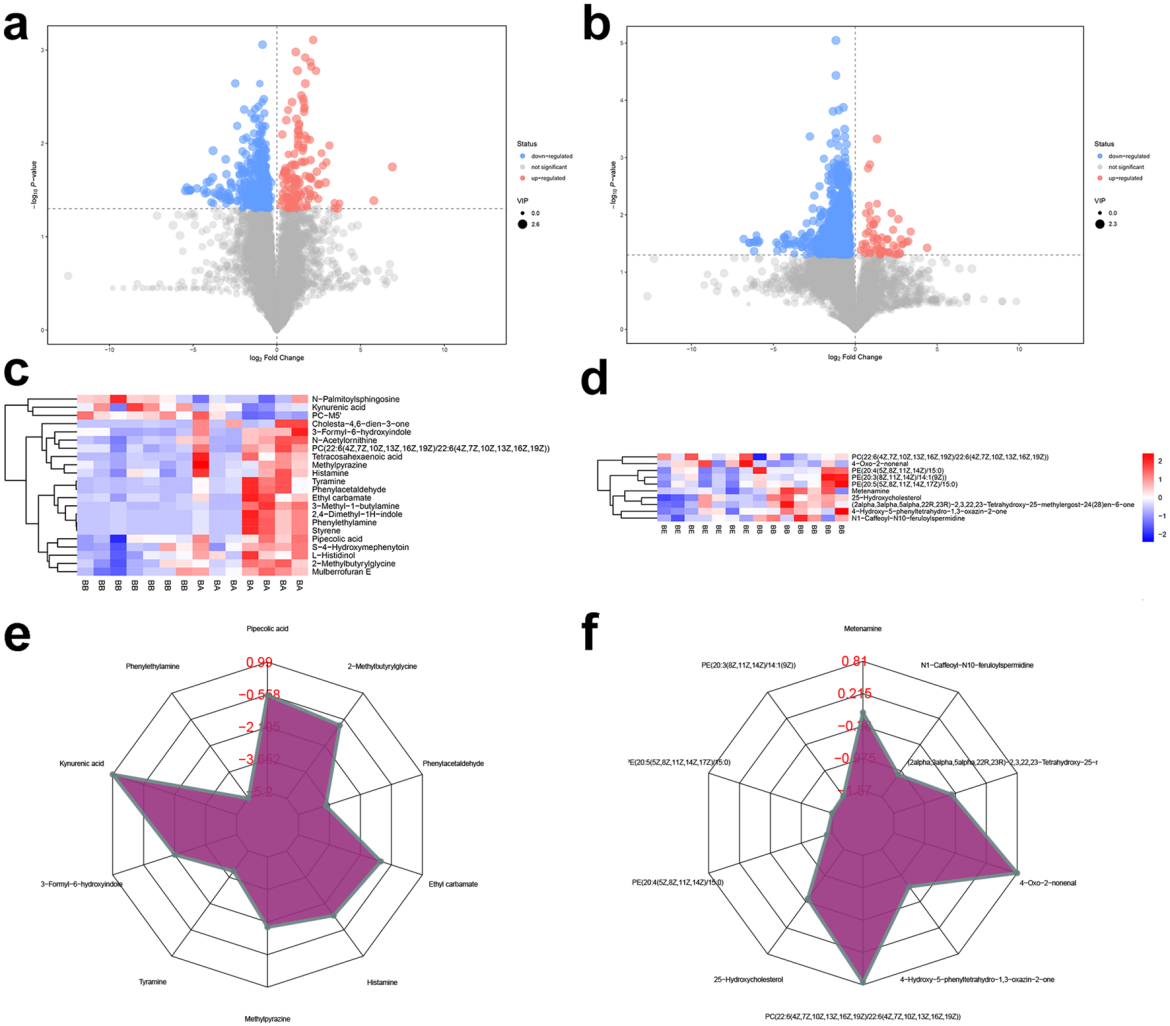
Differential analysis of intestinal metabolites in POS (**a** volcano map of model/blank comparison group; **b** volcano map of MXSGD/model comparison group; **c** cluster heatmap of model/blank comparison group; **d** cluster heatmap of MXSGD/model comparison group; **e** radar plot of top 10 metabolites in model/blank comparison group; **f** radar plot of top 10 metabolites in MXSGD/model comparison group)

**Fig. 15:**
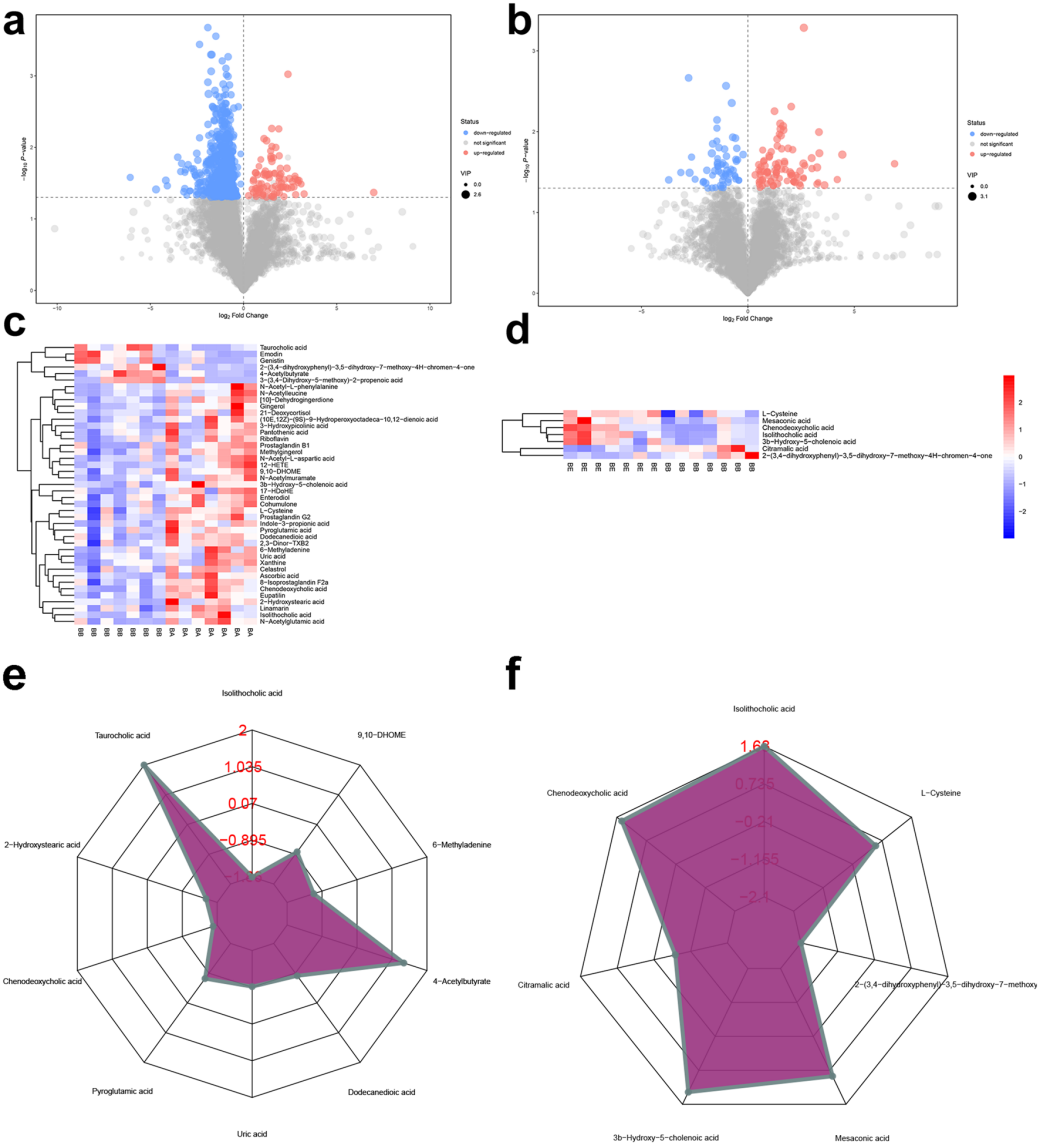
Differential analysis of intestinal metabolites in NES (**a** volcano map of model/blank comparison group; **b** volcano map of MXSGD/model comparison group; **c** cluster heatmap of model/blank comparison group; **d** cluster heatmap of MXSGD/model comparison group; **e** radar plot of top 10 metabolites in model/blank comparison group; **f** radar plot of top 10 metabolites in MXSGD/model comparison group)

The metabolite identification results were shown in “Additional file 7: Intestinal Metabolite”. In model/blank comparison group, there are 151 different metabolites that can be annotated in POS model, and 138 different metabolites that can be annotated in NES model (Figs. 14, 15 and Additional file 5: Table S4). In MXSGD/model comparison group there are 106 different metabolites that can be annotated in POS model and 31 different metabolites that can be annotated in NES model (Figs. 14, 15 and Additional file 6: Table S5).

#### Differential metabolite enrichment analysis

Differential metabolites were annotated in signaling pathways using Kyoto Encyclopedia of Genes and Genomes (KEGG) and Metaboanalyst (http://www.metaboanalyst.ca/).

**Fig. 16:**
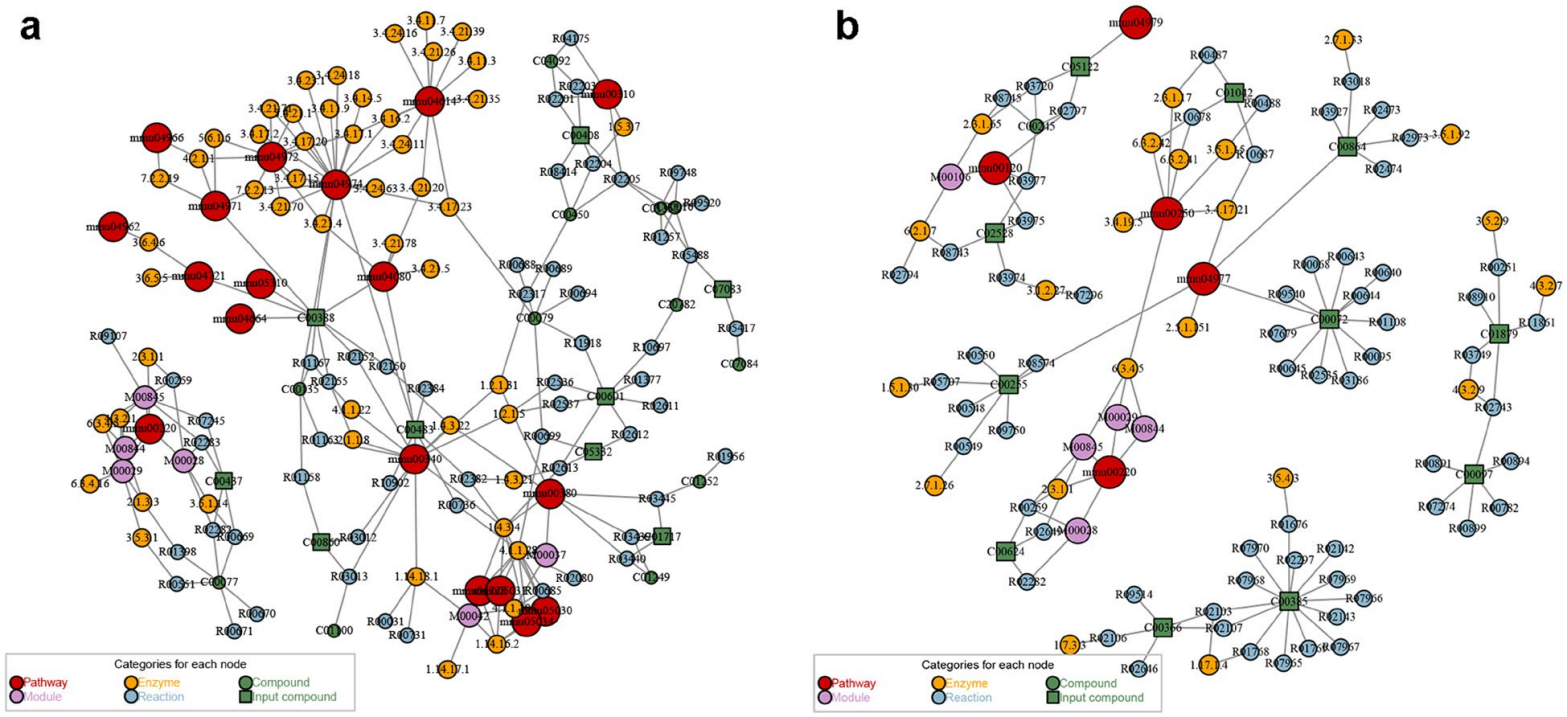
Network plot of pathways in model/blank comparison group (**a** in POS; **b** in NEG)

**Fig. 17:**
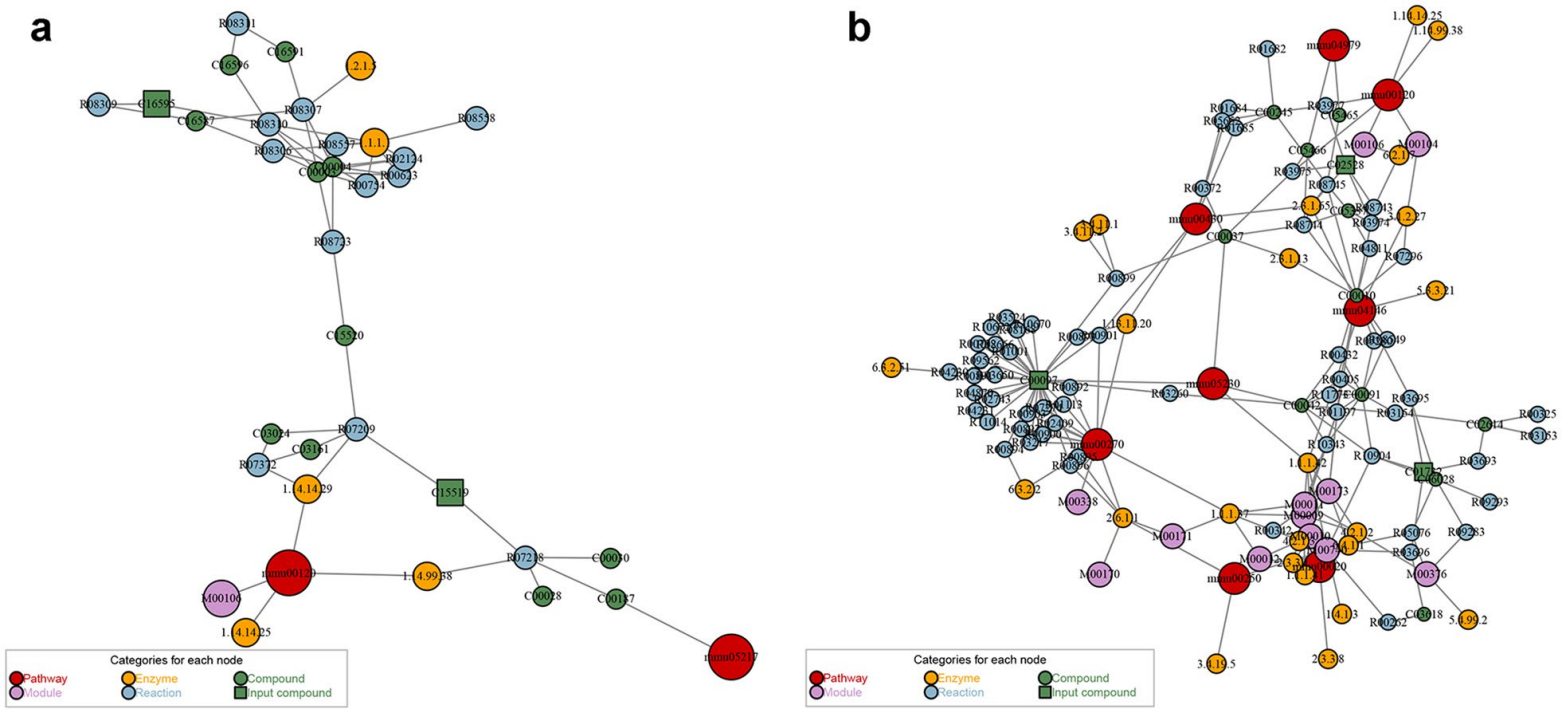
Network plot of pathways in MXSGD/model comparison group (**a** in POS; **b** in NEG)

1. Pathways in model/blank comparison group:
  In POS, the differential metabolites were related to Phenylalanine metabolism, Histidine metabolism, Arginine and proline metabolism, Tyrosine metabolism, while in NES they were related to Taurine and hypotaurine metabolism, Pantothenate and CoA biosynthesis, Glutathione metabolism, Thiamine metabolism, Ascorbate and aldarate metabolism, Riboflavin metabolism, Primary bile acid biosynthesis, Alanine, aspartate and glutamate metabolism, Purine metabolism, Cysteine and methionine metabolism, Glycine, serine and threonine metabolism, Arachidonic acid metabolism, Arginine and proline metabolism, Aminoacyl-tRNA biosynthesis, Steroid hormone biosynthesis (Table 4). After obtaining the matching information of the different metabolites, we performed pathway search and regulatory interaction network analysis on the KEGG database of *Mus musculus* (mouse) (Fig. 16).
2. Pathways in MXSGD/model comparison group:
  In POS, the differential metabolites were related to Primary bile acid biosynthesis, while in NES they were related to Thiamine metabolism, Taurine and hypotaurine metabolism, Pantothenate and CoA biosynthesis, Glutathione metabolism, Cysteine and methionine metabolism, Glycine, serine and threonine metabolism, Primary bile acid biosynthesis, Aminoacyl-tRNA biosynthesis (Table 5). After obtaining the matching information of the different metabolites, we performed pathway search and regulatory interaction network analysis on the KEGG database of *Mus musculus* (mouse) (Fig. 17).

**Table 4:** Pathways in model/blank comparison group

Pattern	Pathway	Counts	*i*-value	FDR	Compounds
POS	Phenylalanine metabolism	2	0.002385	0.19553	Phenylacetaldehyde cpd:C00601; Phenylethylamine cpd:C05332
	Histidine metabolism	1	0.10126	1	Histamine cpd:C00388
	Arginine and proline metabolism	1	0.27128	1	*N*-Acetylornithine cpd:C00437
	Tyrosine metabolism	1	0.27128	1	Tyramine cpd:C00483
NEG	Taurine and hypotaurine metabolism	2	0.0098	0.80358	l-Cysteine cpd:C00097; taurocholic acid cpd:C05122
	Pantothenate and CoA biosynthesis	2	0.033739	1	l-Cysteine cpd:C00097; pantothenic acid cpd:C00864
	Glutathione metabolism	2	0.091433	1	l-Cysteine cpd:C00097; pyroglutamic acid cpd:C01879
	Thiamine metabolism	1	0.13065	1	l-Cysteine cpd:C00097
	Ascorbate and aldarate metabolism	1	0.16484	1	Ascorbic acid cpd:C00072
	Riboflavin metabolism	1	0.19774	1	Riboflavin cpd:C00255
	Primary bile acid biosynthesis	2	0.22973	1	Chenodeoxycholic acid cpd:C02528; taurocholic acid cpd:C05122
	Alanine, aspartate and glutamate metabolism	1	0.38305	1	*N*-Acetyl-l-aspartic acid cpd:C01042
	Purine metabolism	2	0.39257	1	Xanthine cpd:C00385; uric acid cpd:C00366
	Cysteine and methionine metabolism	1	0.41953	1	l-Cysteine cpd:C00097
	Glycine, serine and threonine metabolism	1	0.46496	1	l-Cysteine cpd:C00097
	Arachidonic acid metabolism	1	0.51693	1	Prostaglandin G2 cpd:C05956
	Arginine and proline metabolism	1	0.59012	1	*N*-Acetyl-l-alanine cpd:C00624
	Aminoacyl-tRNA biosynthesis	1	0.75625	1	l-Cysteine cpd:C00097
	Steroid hormone biosynthesis	1	0.77113	1	21-Deoxycortisol cpd:C05497

**Table 5:** Pathways in MXSGD/model comparison group

Pattern	Pathway	Counts	*P*-value	FDR	Compounds
POS	Primary bile acid biosynthesis	1	0.032463	1	25-Hydroxycholesterol cpd:C15519
NEG	Thiamine metabolism	1	0.024491	1	l-Cysteine cpd:C00097
	Taurine and hypotaurine metabolism	1	0.027951	1	l-Cysteine cpd:C00097
	Pantothenate and CoA biosynthesis	1	0.051892	1	l-Cysteine cpd:C00097
	Glutathione metabolism	1	0.088558	1	l-Cysteine cpd:C00097
	Cysteine and methionine metabolism	1	0.091834	1	l-Cysteine cpd:C00097
	Glycine, serine and threonine metabolism	1	0.10485	1	l-Cysteine cpd:C00097
	Primary bile acid biosynthesis	1	0.15231	1	Chenodeoxycholic acid cpd:C02528
	Aminoacyl-tRNA biosynthesis	1	0.22117	1	l-Cysteine cpd:C00097

### Correlation analysis of intestinal microbiota and metabolite

**Fig. 18:**
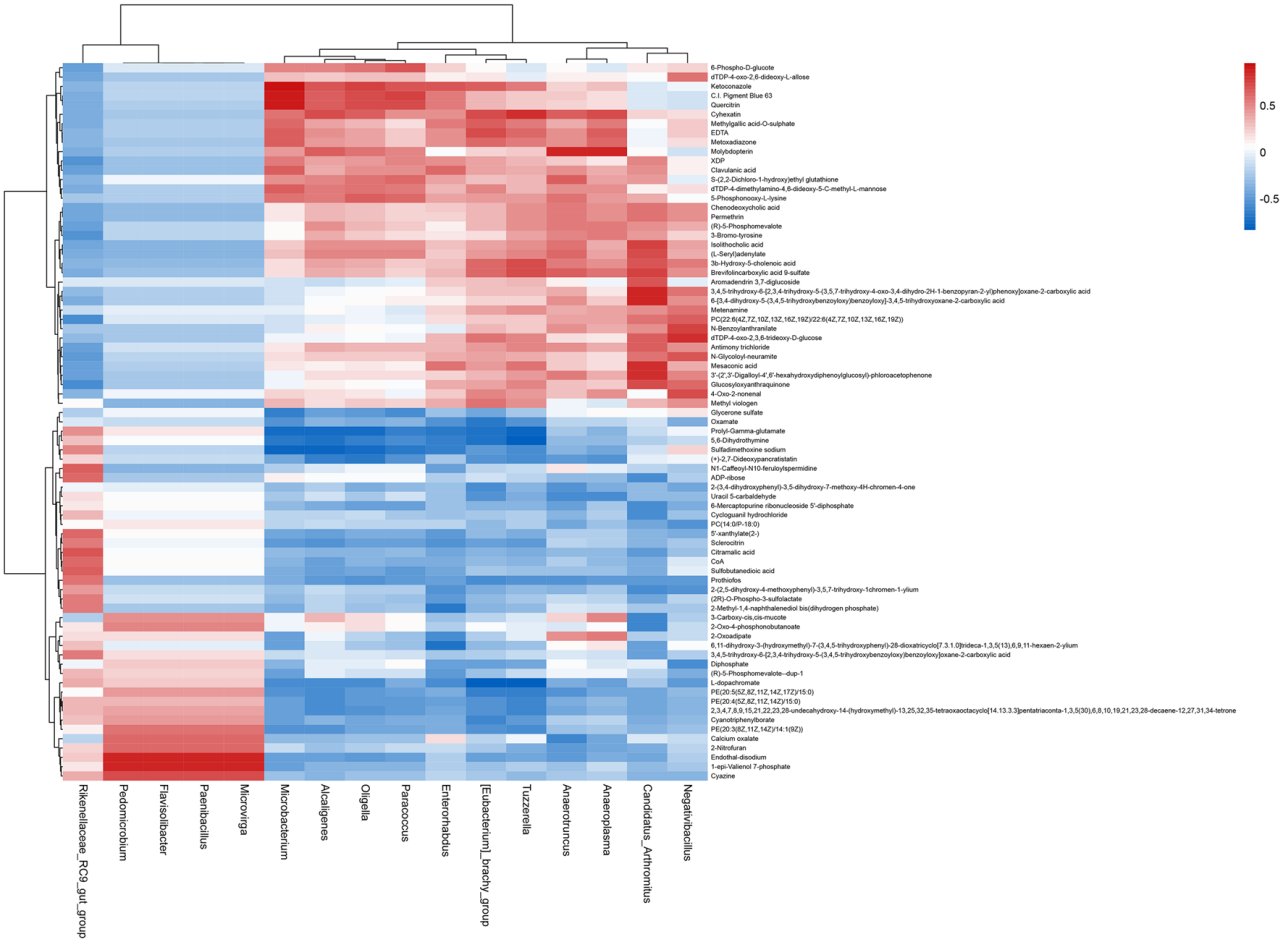
Heatmap of correlations between differential gut microbes and differential metabolites in the MXSGD/model group (red represents positive correlation, blue represents negative correlation)

**Fig. 19:**
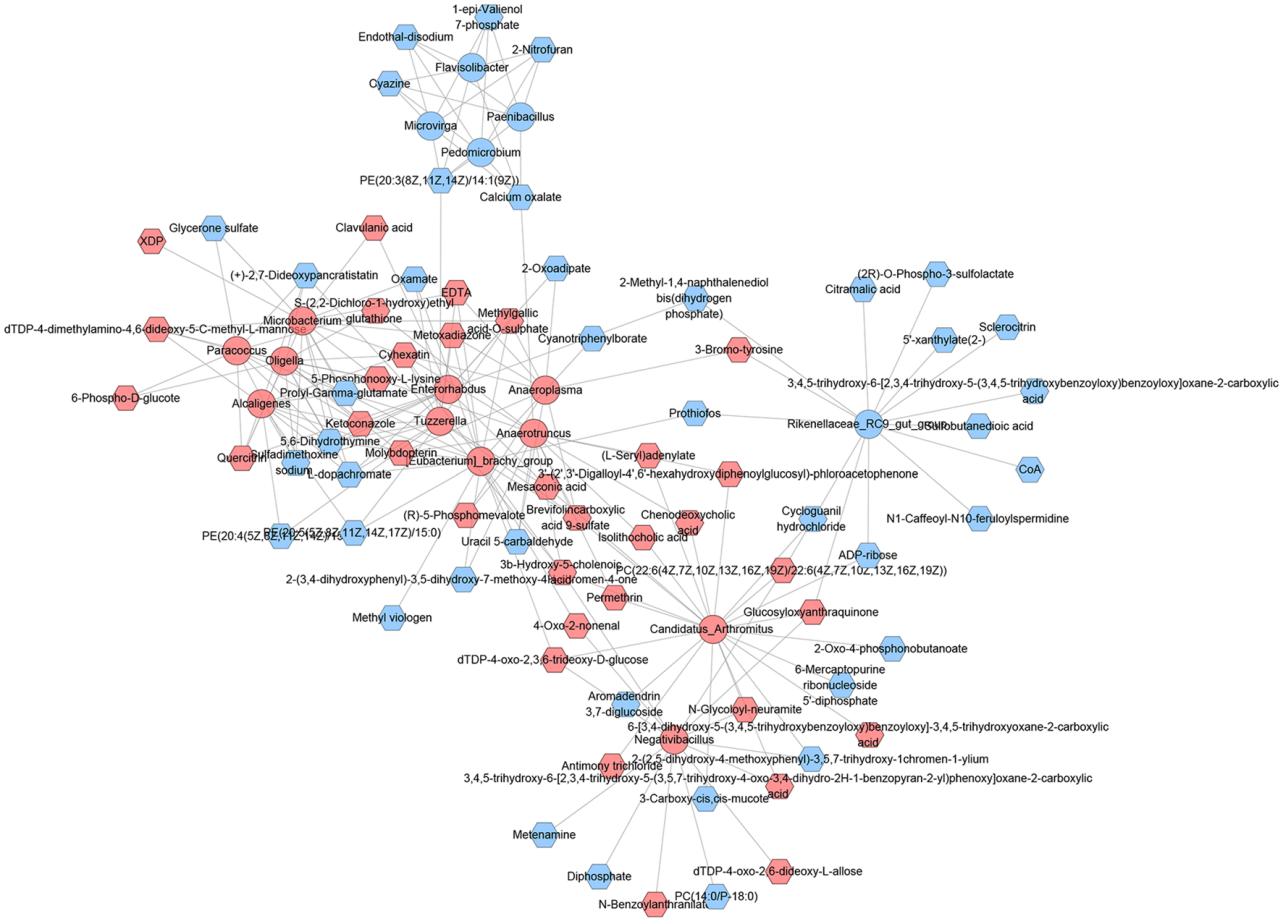
Interactions between differential gut microbes and differential metabolites (circles represent gut microbes and Hexagons represent metabolites. Red stands for up-regulated or increased, blue stands for down-regulated or decreased)

Correlation analysis showed that a series of differentially expressed bacterial groups in the MXSGD group had a certain relationship with the metabolome (Figs. 18 and 19).

#### For down-regulated intestinal microbiota

1. *Flavisolibacter*, *Microvirga*, *Paenibacillus* and *Pedomicrobium* were positively correlated with down-regulation of PE(20:3(8Z,11Z,14Z)/14:1(9Z)), 2-nitrofuran, calcium oxalate, cyazine, endothal-disodium, 1-epi-valienol 7-phosphate.
2. *Rikenellaceae_RC9_gut_group* was negatively correlated with up-regulation of glucosyloxyanthraquinone, PC(22:6(4Z,7Z,1–0Z,13Z,16Z,19Z)/22:6(4Z,7Z,10Z,13Z,16Z,19Z)), 3-bromo-tyrosine; and it was positively correlated with 3,4,5-trihydroxy-6-[2,3,4-trihydroxy-5-(3,4,5-trihydroxybenzoyloxy)benzoyloxy]oxane-2-carboxylic acid, (2*R*)-*O*-phospho-3-sulfolactate, 2-methyl-1,4-naphthalenediol bis(dihydrogen phosphate), sclerocitrin, prothiofos, 5′-xanthylate(2-), ADP-ribose, CoA, sulfobutanedioic acid, *N*1-caffeoyl-*N*10-feruloylspermidine, citramalic acid.

#### For up-regulated intestinal microbiota

1. *[Eubacterium]_brachy_group* was positively correlated with up-regulation of 4-oxo-2-nonenal, dTDP-4-oxo-2,3,6-trideoxy-d-glucose, 3b-hydroxy-5-cholenoic acid, metoxadiazone, brevifolincarboxylic acid 9-sulfate, ketoconazole, methylgallic acid-*O*-sulphate, EDTA, cyhexatin; it was negatively correlated with down-regulation of l-dopachromate, 5,6-dihydrothymine, prolyl-gamma-glutamate, oxamate, PE(20:5(5Z,8Z,11Z,14Z,17Z)/15:0), cyanotriphenylborate, prothiofos, 2-(3,4-dihydroxyphenyl)-3,5-dihydroxy-7-methoxy-4*H*-chromen-4-one, uracil 5-carbaldehyde. In addition, it was positively correlated with down-regulation of methyl viologen.
2. *Alcaligenes* was positively correlated with up-regulation of dTDP-4-dimethylamino-4,6-dideoxy-5-C-methyl-l-mannose, 5-phosphonooxy-l-lysine, molybdopterin, quercitrin, ketoconazole, cyhexatin; it was negatively correlated with down-regulation of sulfadimethoxine sodium, prolyl-gamma-glutamate, 5,6-dihydrothymine, (+)-2,7-dideoxypancratistatin, PE(20:5(5Z,8Z,11Z,14Z,17Z)/15:0), PE(20:4(5Z,8Z,11Z,14Z)/15:0), l-dopachromate.
3. *Anaeroplasma* was positively correlated with up-regulation of 3-bromo-tyrosine, (*R*)-5-phosphomevalote, methylgallic acid-*O*-sulphate, brevifolincarboxylic acid 9-sulfate, metoxadiazone, EDTA, cyhexatin, molybdopterin; it was negatively correlated with down-regulation of Uracil 5-carbaldehyde. In addition, it was positively correlated with down-regulation of 2-oxoadipate.
4. *Anaerotruncus* was positively correlated with up-regulation of 3′-(2′,3′-digalloyl-4′,6′-hexahydroxydiphenoylglucosyl)-phloroacetophenone, 3b-hydroxy-5-cholenoic acid, chenodeoxycholic acid, (*R*)-5-phosphomevalote, permethrin, 5-phosphonooxy-l-lysine, isolithocholic acid, (l-seryl)adenylate, brevifolincarboxylic acid 9-sulfate, *S*-(2,2-dichloro-1-hydroxy)ethyl glutathione, cyhexatin, molybdopterin; it was negatively correlated with down-regulation of calcium oxalate, uracil 5-carbaldehyde, 2-(3,4-dihydroxyphenyl)-3,5-dihydroxy-7-methoxy-4*H*-chromen-4-one.
5. *Candidatus_Arthromitus* was positively correlated with up-regulation of *N*-glycoloyl-neuramite, PC(22:6(4Z,7Z,10Z,13Z,16Z,19Z)/22:6(4Z,7Z,10Z,13Z,16Z,19Z)), permethrin, chenodeoxycholic acid, antimony trichloride, dTDP-4-oxo-2,3,6-trideoxy-d-glucose, 3b-hydroxy-5-cholenoic acid, glucosyloxyanthraquinone, (l-seryl)adenylate, brevifolincarboxylic acid 9-sulfate, isolithocholic acid, mesaconic acid, 3′-(2′,3′-digalloyl-4′,6′-hexahydroxydiphenoylglucosyl)-phloroacetophenone, 6-[3,4-dihydroxy-5-(3,4,5-trihydroxybenzoyloxy)benzoyloxy]-3,4,5-trihydroxyoxane-2-carboxylic acid, 3,4,5-trihydroxy-6-[2,3,4-trihydroxy-5-(3,5,7-trihydroxy-4-oxo-3,4-dihydro-2*H*-1-benzopyran-2-yl)phenoxy]oxane-2-carboxylic acid; it was negatively correlated with down-regulation of 3-carboxy-cis,cis-mucote, 2-oxo-4-phosphonobutanoate, cycloguanil hydrochloride, 2-(2,5-dihydroxy-4-methoxyphenyl)-3,5,7-trihydroxy-1chromen-1-ylium, ADP-ribose, 6-mercaptopurine ribonucleoside 5′-diphosphate. In addition, it was positively correlated with down-regulation of aromadendrin 3,7-diglucoside.
6. *Enterorhabdus* was positively correlated with up-regulation of methylgallic acid-*O*-sulphate, quercitrin, mesaconic acid, clavulanic acid, ketoconazole; it was negatively correlated with down-regulation of 2-methyl-1,4-naphthalenediol bis(dihydrogen phosphate), prolyl-gamma-glutamate, sulfadimethoxine sodium, 5,6-dihydrothymine, l-dopachromate, 2-oxoadipate.
7. *Microbacterium* was positively correlated with up-regulation of XDP, 5-phosphonooxy-l-lysine, cyhexatin, methylgallic acid-*O*-sulphate, EDTA, clavulanic acid, dTDP-4-dimethylamino-4,6-dideoxy-5-C-methyl-l-mannose, metoxadiazone, quercitrin, ketoconazole; it was negatively correlated with down-regulation of sulfadimethoxine sodium, prolyl-gamma-glutamate, 5,6-dihydrothymine, glycerone sulfate, l-dopachromate, oxamate.
8. *Negativibacillus* was positively correlated with up-regulation of 3b-hydroxy-5-cholenoic acid, 3,4,5-trihydroxy-6-[2,3,4-trihydroxy-5-(3,5,7-trihydroxy-4-oxo-3,4-dihydro-2*H*-1-benzopyran-2-yl)phenoxy]oxane-2-carboxylic acid, dTDP-4-oxo-2,6-dideoxy-l-allose, glucosyloxyanthraquinone, PC(22:6(4Z,7Z,10Z,13Z,16Z,19Z)/22:6(4Z,7Z,10Z,13Z,16Z,19Z)), *N*-glycoloyl-neuramite, 4-oxo-2-nonenal, *N*-benzoylanthranilate, dTDP-4-oxo-2,3,6-trideoxy-d-glucose; it was negatively correlated with down-regulation of diphosphate, PC(14:0/P-18:0), 2-(2,5-dihydroxy-4-methoxyphenyl)-3,5,7-trihydroxy-1chromen-1-ylium. In addition, it was positively correlated with down-regulation of metenamine.
9. *Oligella* was positively correlated with up-regulation of 6-phospho-d-glucote, molybdopterin, dTDP-4-dimethylamino-4,6-dideoxy-5-C-methyl-l-mannose, *S*-(2,2-dichloro-1-hydroxy)ethyl glutathione, 5-phosphonooxy-l-lysine, cyhexatin, quercitrin, ketoconazole; it was negatively correlated with down-regulation of prolyl-gamma-glutamate, sulfadimethoxine sodium, (+)-2,7-dideoxypancratistatin, 5,6-dihydrothymine, l-dopachromate.
10. *Paracoccus* was positively correlated with up-regulation of dTDP-4-dimethylamino-4,6-dideoxy-5-C-methyl-l-mannose, 5-phosphonooxy-l-lysine, *S*-(2,2-dichloro-1-hydroxy)ethyl glutathione, ketoconazole, 6-phospho-d-glucote, quercitrin; it was negatively correlated with down-regulation of sulfadimethoxine sodium, prolyl-gamma-glutamate, glycerone sulfate, 5,6-dihydrothymine, (+)-2,7-dideoxypancratistatin.
11. *Tuzzerella* was positively correlated with up-regulation of metoxadiazone, ketoconazole, methylgallic acid-*O*-sulphate, mesaconic acid, EDTA, 3b-hydroxy-5-cholenoic acid, brevifolincarboxylic acid 9-sulfate, cyhexatin; it was negatively correlated with down-regulation of l-dopachromate, 5,6-dihydrothymine, prolyl-gamma-glutamate, PE(20:5(5Z,8Z,11Z,14Z,17Z)/15:0), PE(20:4(5Z,8Z,11Z,14Z)/15:0), sulfadimethoxine sodium, PE(20:3(8Z,11Z,14Z)/14:1(9Z)), (+)-2,7-dideoxypancratistatin, oxamate.

Overall, the intestinal microbiome and metabolomics results show that MXSGD may improve intestinal microbial metabolites by regulating intestinal microorganisms, thereby improving influenza pneumonia through related signaling pathways such as primary bile acid biosynthesis and thiamine metabolism.

## Discussion

Current studies showed that after influenza virus infects the human body, it can induce a cytokine storm, leading to systemic inflammation. In severe cases, acute respiratory distress syndrome (ARDS), shock or multiple organ failure can occur [29]. Among them, the influenza virus-mediated cytokine storm is manifested as pro-inflammatory cytokines, including cytokines directly induced by virus infection and uncontrollable release of downstream cytokines [30]. Immune inflammatory cells are rapidly activated under the stimulation of pro-inflammatory cytokines [IL-1, tumor necrosis factor-α (TNF-α) or interferon-gamma (IFN-γ)], cell stress and bacterial lipopolysaccharide, chemotaxis, proliferate, and differentiate, enter the imbalance of the immune inflammatory cell network, and form a vicious circle with each other. This further promotes the release of inflammatory chemokines, and ultimately leads to the programmed death of respiratory endothelial cells and other structural cells, and at the same time forms an inflammatory blow to important organs throughout the body [31–33]. For example, autopsy of severe influenza patients showed severe diffuse alveolar damage in the lungs of influenza patients; and there is a large number of neutrophil infiltrations in the bronchiole lumen, and a large number of macrophage infiltration can be seen in the alveoli and lung interstitial [34]. Severe pathological changes in the lungs are closely related to the mortality of patients. Studies have shown that neutrophils and mononuclear macrophages are the main reasons for the increase in the number of total lung white blood cells after influenza virus infection, and are the main cell types that cause severe lung pathological damage due to influenza virus infection [35, 36]. Macrophages are involved in the synthesis of cytokines (IL-1β, IL-8, IL-18, CCL3 and IFN-α/β) to aggravate the inflammatory response at the site of inflammation [37]. Influenza virus infection induces the formation of a cytokine storm, under the action of various chemokines, neutrophils, monocytes–macrophages, etc. migrate to the site of inflammation to play a role [38]. In addition, the migration of leukocytes across the endothelium is also a key step for leukocytes to enter inflammation, injury and immune response sites [39].

Through transcriptomics and bioinformatics analysis, we found that the main biological modules effected by the treatment of MXSGD include: inflammatory factors and inflammation-related signal pathways mediated inflammation biological modules (GO:0002694, GO:0002376, GO:0002764); Immune cells (T, B cells) activate chemotaxis, proliferation and differentiation and other immune response modules (GO:0050864, GO:0051249, GO:0002696, GO:0050778, GO:0002250, GO:0002443, GO:0030217); Chemotactic biological module of immune cells mediated by chemokine-related signaling pathways (GO:0001819, GO:0050727); Neutrophils migration and chemotaxis, proliferation and neutrophil respiratory burst and other neutrophil biological modules (GO:0005746, GO:1990204, GO:0030593); Inflammation of the mononuclear macrophage system (GO:0032944, GO:0070469), etc. These biological network modules are involved in the pathological process of influenza virus pneumonia; such as, extensive necrosis of bronchial and bronchiolar cells, accompanied by ciliary epithelial cell shedding, fibrin exudation, inflammatory cell infiltration, hyaline membrane formation, alveolar and bronchial epithelial cell congestion, interstitial edema, mononuclear cell infiltration and other pathological changes [40, 41]. Of note, it is imperative to integrate the enrichment analysis results of the biological processes and signaling pathways associated with up-regulated and down-regulated genes to authentically depict the mechanisms underlying MXSGD intervention in influenza virus A-induced pneumonia.

With the extensive development of intestinal microecology-related research, current research has found that the intestinal microbiota plays an important role in the pathogenesis and prevention of various pulmonary diseases such as asthma, fibrosis and bacterial infection. Axis’ key pivot [42–44]. There is also a close relationship between influenza virus pneumonia and intestinal microecology. By improving the intestinal flora, it has a good preventive effect on respiratory virus infection [45]. It may be to enhance the intestinal mucosal barrier function to reduce secondary bacterial infection; improve the antiviral immune function of innate immune cells, through bacterial components or metabolites such as short-chain fatty acids, tryptophan metabolites, etc. and regulate the balance of Th17/Treg to suppress excessive inflammatory responses [46–48]. In addition, in terms of the mutual regulation of the lung–gut axis, current studies have shown that the gut-lung communication includes at least five pathways [49–53]: (1) Short-chain fatty acids such as butyric acid, acetic acid, and propionic acid produced by the fermentation of dietary fiber and starch by intestinal microorganisms can enter the lung tissue through the blood circulation; (2) Unmetabolized short-chain fatty acids can enter the peripheral blood circulatory system and bone marrow to further affect the development of their immune cells; (3) Bone marrow-derived immune cells elicit immune responses in distant body sites such as lung tissue; (4) Intestinal immune cells such as IC2s, ILC3 and TH17L can also directly migrate from the intestinal tract to the respiratory tract through the blood circulation to affect the immune activity of the respiratory system; (5) The microbial metabolite deaminotyrosine (DAT) protects the host from influenza virus infection by enhancing the type I interferon (IFN) response. In addition to short-chain fatty acids and deaminotyrosine, gut microbial metabolites known to have immunomodulatory effects include products of indole derivatives, dietary tryptophan metabolites, nicotine, polyamines, uroflavin and pyruvate, etc. Therefore, after exploring the molecular biological network regulation of MXSGD intervening in mice with influenza virus through transcriptomics, this study further explored whether MXSGD regulated the intestinal microbial disturbance in mice with influenza virus by means of the 16s technology of gut microbiota. Our research found that the richness and diversity of the intestinal microbiome of the model group mice were significantly reduced. The results of Beta diversity analysis showed that the composition of the intestinal microbiome of the model group was not significantly different from that of the control group and the MXSGD group. At the phylum level, in high abundance flora, compared with the control group, *Actinobacteriota*, *unidentified_Bacteria Cyanobacteria*, *Gemmatimonadota*, *RCP2-54*, *Zixibacteria*, *Kapabacteria* in model group decreased in model was decreased. At Genus level, compared with control group, *Desulfovibrio*, *Enterorhabdus*, *Bifidobacterium*, *Monoglobus*, *Gemella*, *Gaiella*, *Anaerostipes*, *RB41*, *Ellin6055*, *Bryobacter*, *[Eubacterium]_ventriosum_group*, *Ellin6067*, *Skermanella*, *Subgroup_10*, *Lysobacter*, *[Eubacterium]_brachy_group*, *Pseudomonas*, *Streptomyces*, *Paracoccus*, etc. in model group decreased, while *unidentified_Chloroplas and Rikenellaceae_RC9_gut_group* increased (*p* < 0.05).

After MXSGD treatment, both the Alpha diversity analysis and the Beta diversity analysis showed that there was no significant difference between the MXSGD group and the model group. This may be due to the short administration time and the intestinal microbiome has not changed significantly. However, the composition of the intestinal microbiome changes after MXSGD intervention. Compared with model group, the intestinal microbiome *Actinobacteriota* level and *Desulfobacterota* level increased in MXSGD group, while *Planctomycetota* in MXSGD group decreased. Compared with model group, *Paenibacillus*, *Rikenellaceae_RC9_gut_group*, *Microvirga*, *Flavisolibacter*, *Pedomicrobium* in MXSGD group decreased, while *Enterorhabdus*, *Candidatus_Arthromitus*, *Anaerotruncus*, *Anaeroplasma*, *Alcaligenes*, *[Eubacterium]_brachy_group*, *Microbacterium*, *Negativibacillus*, *Oligella*, *Paracoccus*, *Tuzzerella* in MXSGD group increased. Based on our results, it can be speculated that influenza A virus infection may cause intestinal flora structure disorder and immune function imbalance in mice, and MXSGD has a certain protective effect on intestinal immune damage caused by influenza virus by regulating the intestinal microbiota structure.

To further explore the mechanism of MXSGD, we performed a fecal metabolomic analysis of the same cohort of intervention mice with 16s sequencing. We found that cyhexatin, diphosphate, 1-(2-hydroxyphenylamino)-1-deoxy-beta-d-gentiobioside 1,2-carbamate, 3-carboxy-cis,cis-mucote, dihydrokaempferol, 5-amino-4-imidazolecarboxyamide, 6-({13,14-dihydroxy-9-oxo-8,17-dioxatetracyclo[8.7.0.0,0.0,]heptadeca-1(10),2(7),3,5,11(16),12,14-heptaen-5-yl}oxy)-3,4,5-trihydroxyoxane-2-carboxylic acid, caffeoyl aspartic acid, clavulanic acid, isolithocholic acid, chenodeoxycholic acid, citramalic acid, 3b-hydroxy-5-cholenoic acid, mesaconic acid, 2-(3,4-dihydroxyphenyl)-3,5-dihydroxy-7-methoxy-4*H*-chromen-4-one, l-cysteine, etc. was related to MXSGD’s intervention, which is also related to several metabolic pathways such as Thiamine metabolism, Taurine and hypotaurine metabolism, Pantothenate and CoA biosynthesis, Glutathione metabolism, Cysteine and methionine metabolism. Correlation analysis of gut microbiota results with gut metabolomic results also showed strong associations of these metabolites with microbiota. For example, *Flavisolibacter*, *Microvirga*, *Paenibacillus* and *Pedomicrobium* were positively correlated with down-regulation of PE(20:3(8Z,11Z,14Z)/14:1(9Z)), 2-nitrofuran, calcium oxalate, cyazine, endothal-disodium, 1-epi-Valienol 7-phosphate, which suggests that the reduction of *Flavisolibacter*, *Microvirga*, *Paenibacillus* and *Pedomicrobium* is closely related to those chemical components. In summary, correlation evidence from gut microbiome and metabolomics in this study suggests that MXSGD may play an anti-inflammatory and immunoregulatory role by regulating intestinal microbiome (such as decrease *Flavisolibacter*, *Microvirga*, *Paenibacillus* and *Pedomicrobium*) and intestinal metabolic small molecules (such as decrease 2-nitrofuran, calcium oxalate, cyazine, endothal-disodium, 1-epi-valienol 7-phosphate), and ultimately play a potential role in the treatment of influenza A virus pneumonia. However, because this study only detected intestinal flora and intestinal metabolites, it only revealed the correlation between these bacteria and metabolites. Although we refer to the literature reports on the causal relationship between other intestinal flora and metabolites, the results of this study do not constitute a complete causal relationship. Therefore, in the future, we will further explore the causal relationship between intestinal flora and intestinal metabolites through more in-depth research and experiments.

The limitation of this research is that the interaction of MXSGD components before and after influenza A virus infection has not been studied, and no specific examination of the regulation of transcription and post-translational modifications was performed. We hope to conduct more in-depth experiments in the future, such as tracking changes in gene expression over time or response to therapeutic intervention, to further explore the pharmacokinetics, serum pharmacology, component interactions, etc. of MXSGD components.

## Conclusion

In summary, those results showed that mice with influenza A virus pneumonia had a metabolic disorder of metabolites on the “lung–gut” axis, while MXSGD may play an anti-inflammatory and immunoregulatory role by regulating intestinal microbiome (such as decrease *Flavisolibacter*, *Microvirga*, *Paenibacillus* and *Pedomicrobium*) and intestinal metabolic small molecules (such as decrease 2-nitrofuran, calcium oxalate, cyazine, endothal-disodium, 1-epi-valienol 7-phosphate), and ultimately play a potential role in the treatment of influenza A virus pneumonia.

### Supplementary Information

**Additional file 1: Figure S1.** The result of HPLC (A: reference substance solution; B: MXSGD sample solution).

**Additional file 2: Table S1.** Enrichment analysis of up-regulated Gene.

**Additional file 3: Table S2.** Enrichment analysis of down-regulated gene.

**Additional file 4: Table S3.** Enrichment analysis of all gene.

**Additional file 5: Table S4.** Top 20 different metabolites of model/blank comparison.

**Additional file 6: Table S5.** Top 20 different metabolites of MXSGD/model comparison.

**Additional file 7.** Intestinal metabolite.

## Data Availability

The data used to support the findings of this study are included within the article and GEO database (GSE211125).
