# Supplementary figures and images for: Exploring the immune-inflammatory mechanism of Maxing Shigan Decoction in treating influenza virus A-induced pneumonia based on an integrated strategy of single-cell transcriptomics and systems biology
Source: Eur J Med Res. 2024 Apr 15;29:234. doi: 10.1186/s40001-024-01777-9 (PMC11017673; doi:10.1186/s40001-024-01777-9)

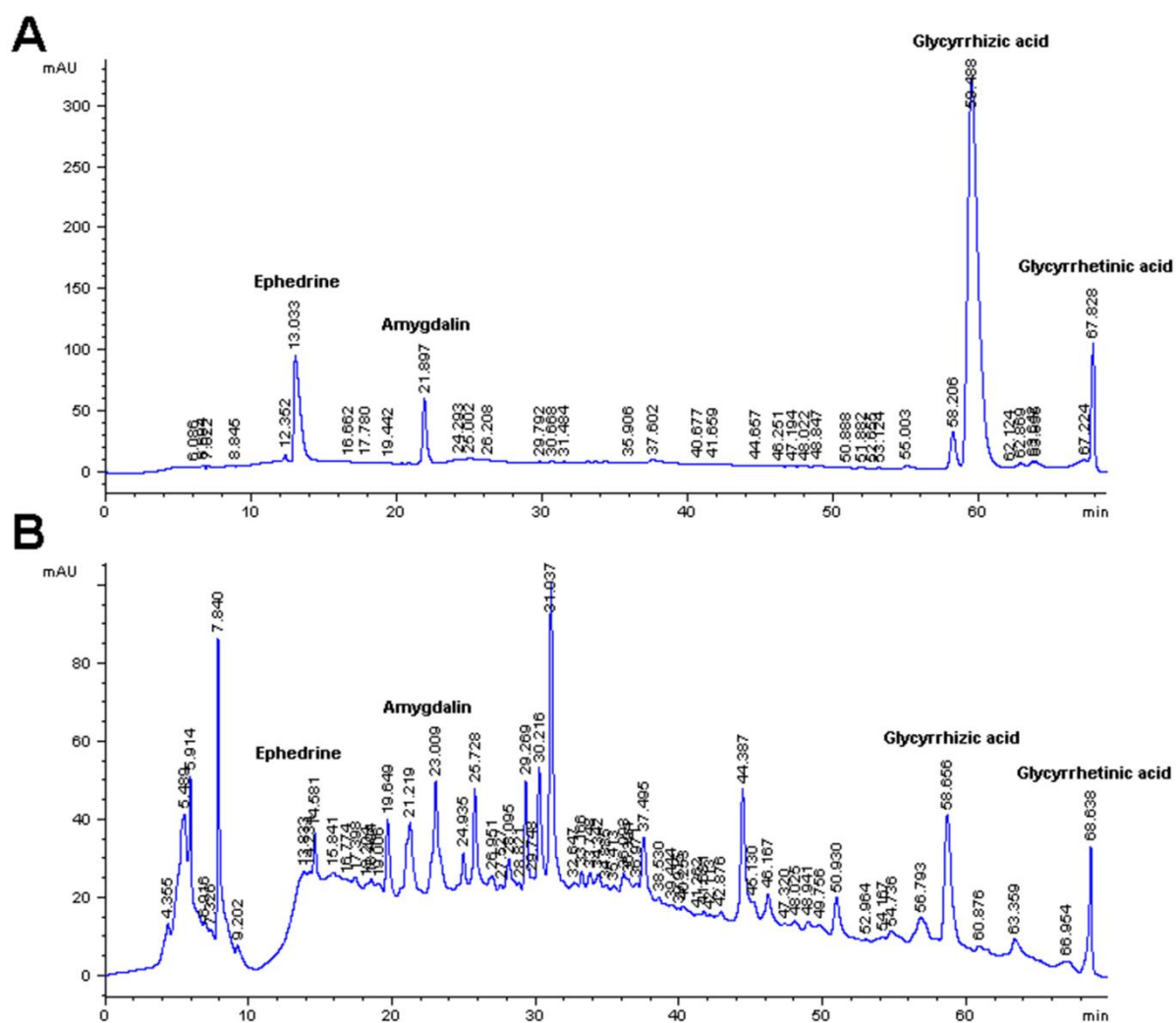

Figure 1 The result of HPLC (A: Reference substance solution; B: MXSGD sample solution)

Supplement: Supplementary file 1 — Additional file 1: Figure S1. The result of HPLC (A: reference substance solution; B: MXSGD sample solution). [file 40001_2024_1777_MOESM1_ESM.pdf]
