# Supplementary material for: Exploring the immune-inflammatory mechanism of Maxing Shigan Decoction in treating influenza virus A-induced pneumonia based on an integrated strategy of single-cell transcriptomics and systems biology
Source: Eur J Med Res. 2024 Apr 15;29:234. doi: 10.1186/s40001-024-01777-9 (PMC11017673; doi:10.1186/s40001-024-01777-9)
Supplement: Supplementary file 2 — Additional file 2: Table S1. Enrichment analysis of up-regulated Gene. [file 40001_2024_1777_MOESM2_ESM.docx]

Table S1 Enrichment analysis of Up-regulated Gene

| **Category** | **GO** | **Description** | **LogP** | **Enrichment** | **Counts** | **Genes** |
| --- | --- | --- | --- | --- | --- | --- |
| GO Biological Processes | GO:0006119 | oxidative phosphorylation | -15 | 8.7 | 24 | Atp5k\|Cox6a2\|Cox7a1\|ATP6\|ATP8\|COX1\|COX2\|COX3\|CYTB\|ND1\|ND2\|ND3\|ND4\|ND5\|ND6\|Uqcrq\|Nupr1\|Uqcr10\|Uqcrh\|Atp5e\|Slc25a33\|Chchd10\|Ndufb6\|Ndufs6 |
| GO Biological Processes | GO:0046034 | ATP metabolic process | -14 | 5.2 | 33 | Ak1\|Aldoa\|Atp5g1\|Atp5k\|Bcl2l1\|Cox6a2\|Cox7a1\|Gpd1\|ATP6\|ATP8\|COX1\|COX2\|COX3\|CYTB\|ND1\|ND2\|ND3\|ND4\|ND5\|ND6\|Uqcrq\|Nupr1\|Uqcr10\|Uqcrh\|Atp5e\|Slc25a33\|Atp1a2\|Chchd10\|Gck\|Atp6v1b1\|Atp5g3\|Ndufb6\|Ndufs6 |
| GO Biological Processes | GO:0022900 | electron transport chain | -13 | 8.6 | 20 | Cox6a2\|Gpd1\|COX1\|COX2\|COX3\|CYTB\|ND1\|ND2\|ND3\|ND4\|ND5\|ND6\|Uqcrq\|Uqcr10\|Ndufb3\|Uqcrh\|Ndufa5\|Ndufb6\|Ndufs6\|Wdr93 |
| GO Biological Processes | GO:0045333 | cellular respiration | -12 | 5.3 | 28 | Atp5k\|Cox6a2\|Cox7a1\|Gpd1\|ATP6\|ATP8\|COX1\|COX2\|COX3\|CYTB\|ND1\|ND2\|ND3\|ND4\|ND5\|ND6\|Uqcrq\|Nupr1\|Uqcr10\|Uqcrh\|Atp5e\|Mtln\|Ndufa5\|Slc25a33\|Chchd10\|Coq10a\|Ndufb6\|Ndufs6 |
| GO Biological Processes | GO:0033108 | mitochondrial respiratory chain complex assembly | -12 | 8.5 | 19 | ND1\|ND2\|ND4\|ND5\|ND6\|Ndufa2\|Ndufa1\|Ndufa3\|Ndufc1\|Cox14\|Ndufb3\|Ndufa5\|Sdhaf1\|Ndufb10\|Slc25a33\|Ndufaf8\|Ndufb6\|Ndufs6\|Pet100 |
| GO Biological Processes | GO:0015980 | energy derivation by oxidation of organic compounds | -12 | 4.4 | 33 | Atp5k\|Cox6a2\|Cox7a1\|Gpd1\|Gys1\|ATP6\|ATP8\|COX1\|COX2\|COX3\|CYTB\|ND1\|ND2\|ND3\|ND4\|ND5\|ND6\|Mt3\|Phkg1\|Uqcrq\|Nupr1\|Ppp1r1a\|Uqcr10\|Uqcrh\|Atp5e\|Mtln\|Ndufa5\|Slc25a33\|Chchd10\|Gck\|Coq10a\|Ndufb6\|Ndufs6 |
| GO Biological Processes | GO:0009060 | aerobic respiration | -12 | 6.1 | 24 | Atp5k\|Cox6a2\|Cox7a1\|ATP6\|ATP8\|COX1\|COX2\|COX3\|CYTB\|ND1\|ND2\|ND3\|ND4\|ND5\|ND6\|Uqcrq\|Nupr1\|Uqcr10\|Uqcrh\|Atp5e\|Slc25a33\|Chchd10\|Ndufb6\|Ndufs6 |
| GO Biological Processes | GO:0019646 | aerobic electron transport chain | -12 | 12 | 15 | Cox6a2\|COX1\|COX3\|CYTB\|ND1\|ND2\|ND3\|ND4\|ND5\|ND6\|Uqcrq\|Uqcr10\|Uqcrh\|Ndufb6\|Ndufs6 |
| GO Biological Processes | GO:0022904 | respiratory electron transport chain | -12 | 8.5 | 18 | Cox6a2\|Gpd1\|COX1\|COX2\|COX3\|CYTB\|ND1\|ND2\|ND3\|ND4\|ND5\|ND6\|Uqcrq\|Uqcr10\|Uqcrh\|Ndufa5\|Ndufb6\|Ndufs6 |
| GO Biological Processes | GO:0042773 | ATP synthesis coupled electron transport | -11 | 9.8 | 16 | Cox6a2\|COX1\|COX2\|COX3\|CYTB\|ND1\|ND2\|ND3\|ND4\|ND5\|ND6\|Uqcrq\|Uqcr10\|Uqcrh\|Ndufb6\|Ndufs6 |
| GO Biological Processes | GO:0032981 | mitochondrial respiratory chain complex I assembly | -11 | 11 | 15 | ND1\|ND2\|ND4\|ND5\|ND6\|Ndufa2\|Ndufa1\|Ndufa3\|Ndufc1\|Ndufb3\|Ndufa5\|Ndufb10\|Ndufaf8\|Ndufb6\|Ndufs6 |
| GO Biological Processes | GO:0010257 | NADH dehydrogenase complex assembly | -11 | 11 | 15 | ND1\|ND2\|ND4\|ND5\|ND6\|Ndufa2\|Ndufa1\|Ndufa3\|Ndufc1\|Ndufb3\|Ndufa5\|Ndufb10\|Ndufaf8\|Ndufb6\|Ndufs6 |
| GO Biological Processes | GO:0006091 | generation of precursor metabolites and energy | -11 | 3.5 | 37 | Aldoa\|Atp5k\|Cox6a2\|Cox7a1\|Gpd1\|Gys1\|ATP6\|ATP8\|COX1\|COX2\|COX3\|CYTB\|ND1\|ND2\|ND3\|ND4\|ND5\|ND6\|Mt3\|Phkg1\|Uqcrq\|Slc27a5\|Nupr1\|Ppp1r1a\|Uqcr10\|Ndufb3\|Uqcrh\|Atp5e\|Mtln\|Ndufa5\|Slc25a33\|Chchd10\|Gck\|Coq10a\|Ndufb6\|Ndufs6\|Wdr93 |
| GO Biological Processes | GO:0042775 | mitochondrial ATP synthesis coupled electron transport | -10 | 9.4 | 15 | Cox6a2\|COX1\|COX3\|CYTB\|ND1\|ND2\|ND3\|ND4\|ND5\|ND6\|Uqcrq\|Uqcr10\|Uqcrh\|Ndufb6\|Ndufs6 |
| GO Biological Processes | GO:0044782 | cilium organization | -9.6 | 3.6 | 33 | Foxj1\|Neurl1a\|Rab17\|Tekt1\|Rsph1\|Tekt2\|Cfap298\|Cep70\|Dnai1\|1700012B09Rik\|Daw1\|Tekt4\|Odad4\|Cfap126\|Rsph9\|Zmynd10\|Dcdc2a\|Ulk4\|Odad1\|Cfap157\|Fhdc1\|Cdk10\|Hoatz\|Catip\|Ccdc113\|Kif19a\|Spef2\|Mapk15\|Cfap58\|Lca5l\|Cibar2\|Dnah7a\|Ccdc13 |
| GO Biological Processes | GO:0035082 | axoneme assembly | -9.6 | 7.6 | 16 | Foxj1\|Neurl1a\|Rsph1\|Tekt2\|Dnai1\|Daw1\|Odad4\|Rsph9\|Zmynd10\|Ulk4\|Odad1\|Cfap157\|Hoatz\|Spef2\|Cfap58\|Dnah7a |
| GO Biological Processes | GO:0003341 | cilium movement | -8.2 | 4.4 | 22 | Neurl1a\|Tekt1\|Tekt2\|Dnah10\|Cfap298\|Dnai1\|Daw1\|Tekt4\|Odad4\|Ttll9\|Dnali1\|Rsph9\|Zmynd10\|Ulk4\|Odad1\|Cfap157\|Hoatz\|Ropn1l\|Wdr66\|Spef2\|Cfap58\|Dnah7a |
| GO Biological Processes | GO:0060271 | cilium assembly | -8.1 | 3.5 | 29 | Foxj1\|Neurl1a\|Rab17\|Tekt1\|Rsph1\|Tekt2\|Cfap298\|Cep70\|Dnai1\|1700012B09Rik\|Daw1\|Tekt4\|Odad4\|Rsph9\|Zmynd10\|Dcdc2a\|Ulk4\|Odad1\|Cfap157\|Fhdc1\|Cdk10\|Hoatz\|Ccdc113\|Spef2\|Mapk15\|Cfap58\|Cibar2\|Dnah7a\|Ccdc13 |
| GO Biological Processes | GO:0007005 | mitochondrion organization | -7.8 | 2.9 | 36 | Slc25a4\|Bcl2l1\|Bik\|Cox7a1\|ND1\|ND2\|ND4\|ND5\|ND6\|Ndufa2\|Dynlt1b\|Trp73\|Timm13\|Ndufa1\|Ndufa3\|Tomm7\|Ndufc1\|Cox14\|Ndufb3\|Romo1\|Ndufa5\|Sdhaf1\|Ndufb10\|Slc25a33\|Ggnbp1\|Tymp\|Cstad\|Stpg1\|Rcc1l\|Chchd10\|Ndufaf8\|Ndufb6\|Mief2\|Ndufs6\|Dynlt1c\|Pet100 |
| GO Biological Processes | GO:0030031 | cell projection assembly | -7.7 | 2.8 | 38 | Foxj1\|Hnf4a\|Neurl1a\|Rab17\|Tekt1\|Rsph1\|Tekt2\|Cfap298\|Cep70\|Dnai1\|1700012B09Rik\|Rhou\|Daw1\|Tekt4\|Odad4\|Rsph9\|Mien1\|Zmynd10\|Nlgn1\|Dcdc2a\|Ulk4\|Odad1\|Cfap157\|Rhov\|Fhdc1\|Ablim2\|Cdk10\|Hoatz\|Ccdc113\|Spef2\|Mapk15\|Cfap58\|Spef1l\|Myo1a\|Cibar2\|Dnah7a\|Ccl21b\|Ccdc13 |
| GO Cellular Components | GO:0070469 | respirasome | -20 | 12 | 26 | Cox6a2\|Cox7a1\|COX1\|COX2\|COX3\|CYTB\|ND1\|ND2\|ND3\|ND4\|ND5\|ND6\|Ndufa2\|Uqcrq\|Ndufa1\|Ndufa3\|Uqcr10\|Ndufc1\|Ndufb3\|Uqcrh\|Uqcr11\|Ndufa5\|Ndufb10\|Ndufb6\|Ndufs6\|Wdr93 |
| GO Cellular Components | GO:0098800 | inner mitochondrial membrane protein complex | -19 | 9.1 | 29 | Atp5g1\|Atp5k\|Cox6a2\|ATP6\|ATP8\|COX1\|CYTB\|ND1\|ND2\|ND3\|ND4\|ND5\|Ndufa2\|Uqcrq\|Ndufa1\|Ndufa3\|Uqcr10\|Ndufc1\|Ndufb3\|Uqcrh\|Romo1\|Atp5e\|Ndufa5\|Ndufb10\|Chchd10\|Atp5g3\|Ndufb6\|Ndufs6\|Wdr93 |
| GO Cellular Components | GO:0098803 | respiratory chain complex | -18 | 11 | 23 | Cox6a2\|COX1\|COX2\|COX3\|CYTB\|ND1\|ND2\|ND3\|ND4\|ND5\|Ndufa2\|Uqcrq\|Ndufa1\|Ndufa3\|Uqcr10\|Ndufc1\|Ndufb3\|Uqcrh\|Ndufa5\|Ndufb10\|Ndufb6\|Ndufs6\|Wdr93 |
| GO Cellular Components | GO:0005746 | mitochondrial respirasome | -17 | 12 | 22 | Cox6a2\|Cox7a1\|COX1\|CYTB\|ND1\|ND2\|ND3\|ND4\|ND5\|Ndufa2\|Uqcrq\|Ndufa1\|Ndufa3\|Uqcr10\|Ndufc1\|Ndufb3\|Uqcrh\|Ndufa5\|Ndufb10\|Ndufb6\|Ndufs6\|Wdr93 |
| GO Cellular Components | GO:0005743 | mitochondrial inner membrane | -17 | 4.3 | 48 | Slc25a4\|Atp5g1\|Atp5k\|Bcl2l1\|Cox6a2\|Cox7a1\|Cyp1a1\|Cyp2e1\|Endog\|ATP6\|ATP8\|COX1\|COX2\|COX3\|CYTB\|ND1\|ND2\|ND3\|ND4\|ND5\|ND6\|Ndufa2\|Ucp3\|Uqcrq\|Timm13\|Ndufa1\|Ndufa3\|Uqcr10\|Ndufc1\|Ndufb3\|Uqcrh\|Uqcr11\|Romo1\|Atp5e\|Mtln\|Ndufa5\|Ndufb10\|Tmem256\|Slc25a33\|Rcc1l\|Chchd10\|Atp5g3\|Ndufb6\|Slc25a48\|Slc25a34\|Ndufs6\|Wdr93\|Pet100 |
| GO Cellular Components | GO:1990204 | oxidoreductase complex | -17 | 9.1 | 25 | Gpd1\|COX1\|CYTB\|ND1\|ND2\|ND3\|ND4\|ND5\|Ndufa2\|Pdk2\|Uqcrq\|Ndufa1\|Ndufa3\|Uqcr10\|Gmpr\|Ndufc1\|Ndufb3\|Uqcrh\|Ndufa5\|Ndufb10\|Etfb\|Ndufb6\|Noxa1\|Ndufs6\|Wdr93 |
| GO Cellular Components | GO:0098798 | mitochondrial protein-containing complex | -16 | 5.6 | 36 | Slc25a4\|Atp5g1\|Atp5k\|Cox6a2\|ATP6\|ATP8\|COX1\|CYTB\|ND1\|ND2\|ND3\|ND4\|ND5\|mt-Rnr1\|Ndufa2\|Pdk2\|Uqcrq\|Timm13\|Ndufa1\|Ndufa3\|Uqcr10\|Tomm7\|Ndufc1\|Ndufb3\|Uqcrh\|Romo1\|Atp5e\|Ndufa5\|Ndufb10\|Mrpl14\|Chchd10\|Etfb\|Atp5g3\|Ndufb6\|Ndufs6\|Wdr93 |
| GO Cellular Components | GO:0019866 | organelle inner membrane | -15 | 3.9 | 48 | Slc25a4\|Atp5g1\|Atp5k\|Bcl2l1\|Cox6a2\|Cox7a1\|Cyp1a1\|Cyp2e1\|Endog\|ATP6\|ATP8\|COX1\|COX2\|COX3\|CYTB\|ND1\|ND2\|ND3\|ND4\|ND5\|ND6\|Ndufa2\|Ucp3\|Uqcrq\|Timm13\|Ndufa1\|Ndufa3\|Uqcr10\|Ndufc1\|Ndufb3\|Uqcrh\|Uqcr11\|Romo1\|Atp5e\|Mtln\|Ndufa5\|Ndufb10\|Tmem256\|Slc25a33\|Rcc1l\|Chchd10\|Atp5g3\|Ndufb6\|Slc25a48\|Slc25a34\|Ndufs6\|Wdr93\|Pet100 |
| GO Cellular Components | GO:0005740 | mitochondrial envelope | -14 | 3.3 | 55 | Slc25a4\|Atp5g1\|Atp5k\|Bcl2l1\|Bik\|Cidea\|Cox6a2\|Cox7a1\|Cyp1a1\|Cyp2e1\|Endog\|ATP6\|ATP8\|COX1\|COX2\|COX3\|CYTB\|ND1\|ND2\|ND3\|ND4\|ND5\|ND6\|Mt3\|Ndufa2\|Ucp3\|Uqcrq\|Timm13\|Ndufa1\|Ndufa3\|Uqcr10\|Tomm7\|Ndufc1\|Ndufb3\|Uqcrh\|Uqcr11\|Romo1\|Atp5e\|Mtln\|Ndufa5\|Ndufb10\|Tmem256\|Slc25a33\|Ggnbp1\|Cstad\|Rcc1l\|Chchd10\|Atp5g3\|Ndufb6\|Mief2\|Slc25a48\|Slc25a34\|Ndufs6\|Wdr93\|Pet100 |
| GO Cellular Components | GO:0031966 | mitochondrial membrane | -14 | 3.3 | 52 | Slc25a4\|Atp5g1\|Atp5k\|Bcl2l1\|Cox6a2\|Cox7a1\|Cyp1a1\|Cyp2e1\|Endog\|ATP6\|ATP8\|COX1\|COX2\|COX3\|CYTB\|ND1\|ND2\|ND3\|ND4\|ND5\|ND6\|Mt3\|Ndufa2\|Ucp3\|Uqcrq\|Timm13\|Ndufa1\|Ndufa3\|Uqcr10\|Tomm7\|Ndufc1\|Ndufb3\|Uqcrh\|Uqcr11\|Romo1\|Atp5e\|Mtln\|Ndufa5\|Ndufb10\|Tmem256\|Slc25a33\|Cstad\|Rcc1l\|Chchd10\|Atp5g3\|Ndufb6\|Mief2\|Slc25a48\|Slc25a34\|Ndufs6\|Wdr93\|Pet100 |
| GO Cellular Components | GO:0005929 | cilium | -13 | 3.1 | 54 | Ak1\|Aldoa\|Slc26a3\|Mlf1\|Prom1\|Spa17\|Tcp11\|Rsph1\|Tekt2\|Mok\|Dnah10\|Tbata\|Fank1\|Cfap298\|Dnai1\|Pacrg\|1700001L19Rik\|1700012B09Rik\|Spaca9\|Tekt4\|Odad4\|Ttll9\|Fam183b\|Cfap126\|Dnali1\|Rsph9\|Iqcd\|Enkd1\|Dcdc2a\|Mchr1\|Odad1\|Sntn\|Cfap157\|Fhdc1\|Cdk10\|Hoatz\|Dynlt4\|Cfap100\|Ccdc113\|Ropn1l\|Wdr66\|Kif19a\|Togaram2\|Spef2\|Casc1\|Dnah2\|Ttll10\|Mapk15\|Cngb1\|Cfap58\|Spef1l\|Lca5l\|Cibar2\|Dnah7a |
| GO Cellular Components | GO:0005747 | mitochondrial respiratory chain complex I | -12 | 13 | 15 | ND1\|ND2\|ND3\|ND4\|ND5\|Ndufa2\|Ndufa1\|Ndufa3\|Ndufc1\|Ndufb3\|Ndufa5\|Ndufb10\|Ndufb6\|Ndufs6\|Wdr93 |
| GO Cellular Components | GO:0030964 | NADH dehydrogenase complex | -12 | 13 | 15 | ND1\|ND2\|ND3\|ND4\|ND5\|Ndufa2\|Ndufa1\|Ndufa3\|Ndufc1\|Ndufb3\|Ndufa5\|Ndufb10\|Ndufb6\|Ndufs6\|Wdr93 |
| GO Cellular Components | GO:0045271 | respiratory chain complex I | -12 | 13 | 15 | ND1\|ND2\|ND3\|ND4\|ND5\|Ndufa2\|Ndufa1\|Ndufa3\|Ndufc1\|Ndufb3\|Ndufa5\|Ndufb10\|Ndufb6\|Ndufs6\|Wdr93 |
| GO Cellular Components | GO:1990351 | transporter complex | -9.6 | 3.6 | 33 | Atp5g1\|Cacna1a\|Cacnb1\|Cldn4\|Grik5\|COX1\|CYTB\|ND1\|ND2\|ND3\|ND4\|ND5\|Ndufa2\|Scn1b\|Uqcrq\|Timm13\|Ndufa1\|Trpv6\|Ndufa3\|Uqcr10\|Ndufc1\|Ndufb3\|Uqcrh\|Ndufa5\|Ndufb10\|Kcnip4\|Atp1a2\|Lrrc26\|Atp5g3\|Ndufb6\|Cngb1\|Ndufs6\|Wdr93 |
| GO Cellular Components | GO:1902495 | transmembrane transporter complex | -9.3 | 3.6 | 32 | Atp5g1\|Cacna1a\|Cacnb1\|Cldn4\|Grik5\|COX1\|CYTB\|ND1\|ND2\|ND3\|ND4\|ND5\|Ndufa2\|Scn1b\|Uqcrq\|Ndufa1\|Trpv6\|Ndufa3\|Uqcr10\|Ndufc1\|Ndufb3\|Uqcrh\|Ndufa5\|Ndufb10\|Kcnip4\|Atp1a2\|Lrrc26\|Atp5g3\|Ndufb6\|Cngb1\|Ndufs6\|Wdr93 |
| GO Cellular Components | GO:0031514 | motile cilium | -6.7 | 3.7 | 22 | Ak1\|Aldoa\|Slc26a3\|Mlf1\|Spa17\|Tcp11\|Rsph1\|Tekt2\|Dnai1\|Pacrg\|Spaca9\|Tekt4\|Odad4\|Dnali1\|Rsph9\|Iqcd\|Dynlt4\|Ropn1l\|Wdr66\|Spef2\|Dnah2\|Cfap58 |
| GO Cellular Components | GO:0097729 | 9+2 motile cilium | -6.6 | 4.3 | 18 | Ak1\|Aldoa\|Slc26a3\|Spa17\|Tcp11\|Rsph1\|Dnai1\|Pacrg\|Spaca9\|Tekt4\|Odad4\|Dnali1\|Rsph9\|Dynlt4\|Wdr66\|Spef2\|Dnah2\|Cfap58 |
| GO Cellular Components | GO:0070069 | cytochrome complex | -6 | 9.9 | 8 | Cox6a2\|COX1\|COX2\|COX3\|CYTB\|Uqcrq\|Uqcr10\|Uqcrh |
| GO Cellular Components | GO:0030286 | dynein complex | -5.9 | 7.1 | 10 | Dynlt1b\|Dnah10\|Dnai1\|Dnali1\|Odad1\|Dynlt4\|Casc1\|Dnah2\|Dnah7a\|Dynlt1c |
| GO Molecular Functions | GO:0015453 | oxidoreduction-driven active transmembrane transporter activity | -14 | 13 | 16 | Cox6a2\|Cox7a1\|COX1\|COX2\|COX3\|CYTB\|ND1\|ND2\|ND3\|ND4\|ND5\|ND6\|Ndufa2\|Uqcrq\|Uqcrh\|Uqcr11 |
| GO Molecular Functions | GO:0009055 | electron transfer activity | -12 | 9.9 | 17 | Cox6a2\|Cox7a1\|COX1\|COX2\|COX3\|CYTB\|ND1\|ND2\|ND3\|ND4\|ND5\|ND6\|Ndufa2\|Uqcrq\|Uqcrh\|Uqcr11\|Etfb |
| GO Molecular Functions | GO:0015078 | proton transmembrane transporter activity | -11 | 7 | 19 | Slc25a4\|Atp5g1\|Atp5k\|Cox6a2\|Cox7a1\|ATP6\|ATP8\|COX1\|COX2\|COX3\|CYTB\|Uqcrq\|Uqcrh\|Uqcr11\|Atp5e\|Mfsd3\|Atp6v1b1\|Slc9a2\|Atp5g3 |
| GO Molecular Functions | GO:0015399 | primary active transmembrane transporter activity | -8.2 | 4.8 | 20 | Cox6a2\|Cox7a1\|Slc26a3\|COX1\|COX2\|COX3\|CYTB\|ND1\|ND2\|ND3\|ND4\|ND5\|ND6\|Ndufa2\|Uqcrq\|Uqcrh\|Uqcr11\|Atp5e\|Atp2c2\|Atp1a2 |
| GO Molecular Functions | GO:0022890 | inorganic cation transmembrane transporter activity | -6.7 | 2.6 | 36 | Slc25a4\|Atp5g1\|Atp5k\|Cacna1a\|Cacnb1\|Cox6a2\|Cox7a1\|Grik5\|Hrh1\|Htr1b\|Kcnh3\|ATP6\|ATP8\|COX1\|COX2\|COX3\|CYTB\|Scn1b\|Uqcrq\|Rhbg\|Trpv6\|Uqcrh\|Uqcr11\|Atp5e\|Atp2c2\|Mfsd3\|Slc39a5\|Kcnip4\|Atp1a2\|Atp6v1b1\|Tspoap1\|Kcnj14\|Slc9a2\|Lrrc26\|Atp5g3\|Cngb1 |
| GO Molecular Functions | GO:0030533 | triplet codon-amino acid adaptor activity | -6.5 | 11 | 8 | TrnE\|TrnI\|TrnL1\|TrnL2\|TrnN\|TrnP\|TrnS2\|TrnY |
| GO Molecular Functions | GO:0016679 | oxidoreductase activity, acting on diphenols and related substances as donors | -6.3 | 26 | 5 | Cyp1a1\|CYTB\|Uqcrq\|Uqcrh\|Uqcr11 |
| GO Molecular Functions | GO:0008324 | cation transmembrane transporter activity | -6.2 | 2.5 | 37 | Slc25a4\|Atp5g1\|Atp5k\|Cacna1a\|Cacnb1\|Cox6a2\|Cox7a1\|Grik5\|Hrh1\|Htr1b\|Kcnh3\|ATP6\|ATP8\|COX1\|COX2\|COX3\|CYTB\|Scn1b\|Uqcrq\|Rhbg\|Trpv6\|Uqcrh\|Uqcr11\|Atp5e\|Atp2c2\|Mfsd3\|Slc39a5\|Kcnip4\|Atp1a2\|Atp6v1b1\|Tspoap1\|Kcnj14\|Slc9a2\|Lrrc26\|Atp5g3\|Slc25a48\|Cngb1 |
| GO Molecular Functions | GO:0008137 | NADH dehydrogenase (ubiquinone) activity | -6.2 | 13 | 7 | ND1\|ND2\|ND3\|ND4\|ND5\|ND6\|Ndufa2 |
| GO Molecular Functions | GO:0050136 | NADH dehydrogenase (quinone) activity | -6.2 | 13 | 7 | ND1\|ND2\|ND3\|ND4\|ND5\|ND6\|Ndufa2 |
| GO Molecular Functions | GO:0015318 | inorganic molecular entity transmembrane transporter activity | -6.1 | 2.3 | 40 | Slc25a4\|Aqp7\|Aqp8\|Atp5g1\|Atp5k\|Cacna1a\|Cacnb1\|Cldn4\|Cox6a2\|Cox7a1\|Slc26a3\|Grik5\|Hrh1\|Htr1b\|Kcnh3\|ATP6\|ATP8\|COX1\|COX2\|COX3\|CYTB\|Scn1b\|Uqcrq\|Rhbg\|Trpv6\|Uqcrh\|Uqcr11\|Atp5e\|Atp2c2\|Mfsd3\|Slc39a5\|Kcnip4\|Atp1a2\|Atp6v1b1\|Tspoap1\|Kcnj14\|Slc9a2\|Lrrc26\|Atp5g3\|Cngb1 |
| GO Molecular Functions | GO:0016491 | oxidoreductase activity | -5.9 | 2.2 | 43 | Aldh3a1\|Cox6a2\|Cox7a1\|Cyp1a1\|Cyp2b19\|Cyp2e1\|Cyp46a1\|Pcbd1\|Dio1\|Gpd1\|Gsta2\|COX1\|COX2\|COX3\|CYTB\|ND1\|ND2\|ND3\|ND4\|ND5\|ND6\|Ndufa2\|Pipox\|Uqcrq\|Hao2\|Gmpr\|Prxl2b\|Uqcrh\|Uqcr11\|Ogfod2\|Iyd\|Cyp2c55\|Glrx5\|Lpo\|Akr1c14\|Cyp4f15\|Me3\|Etfb\|Mgst2\|Fmo4\|Acaa1b\|Wdr93\|Hbb-bt |
| GO Molecular Functions | GO:0003955 | NAD(P)H dehydrogenase (quinone) activity | -5.8 | 11 | 7 | ND1\|ND2\|ND3\|ND4\|ND5\|ND6\|Ndufa2 |
| GO Molecular Functions | GO:0003954 | NADH dehydrogenase activity | -5.8 | 11 | 7 | ND1\|ND2\|ND3\|ND4\|ND5\|ND6\|Ndufa2 |
| GO Molecular Functions | GO:0016655 | oxidoreductase activity, acting on NAD(P)H, quinone or similar compound as acceptor | -5.3 | 8.2 | 8 | ND1\|ND2\|ND3\|ND4\|ND5\|ND6\|Ndufa2\|Akr1c14 |
| GO Molecular Functions | GO:0008121 | ubiquinol-cytochrome-c reductase activity | -5.3 | 27 | 4 | CYTB\|Uqcrq\|Uqcrh\|Uqcr11 |
| GO Molecular Functions | GO:0016651 | oxidoreductase activity, acting on NAD(P)H | -4.9 | 5.5 | 10 | ND1\|ND2\|ND3\|ND4\|ND5\|ND6\|Ndufa2\|Gmpr\|Akr1c14\|Wdr93 |
| GO Molecular Functions | GO:0022804 | active transmembrane transporter activity | -4.3 | 2.5 | 24 | Slc25a4\|Cox6a2\|Cox7a1\|Slc26a3\|COX1\|COX2\|COX3\|CYTB\|ND1\|ND2\|ND3\|ND4\|ND5\|ND6\|Ndufa2\|Uqcrq\|Uqcrh\|Uqcr11\|Atp5e\|Atp2c2\|Mfsd3\|Atp1a2\|Slc16a11\|Slc9a2 |
| GO Molecular Functions | GO:0004129 | cytochrome-c oxidase activity | -4.3 | 11 | 5 | Cox6a2\|Cox7a1\|COX1\|COX2\|COX3 |
| GO Molecular Functions | GO:0016675 | oxidoreductase activity, acting on a heme group of donors | -4.1 | 11 | 5 | Cox6a2\|Cox7a1\|COX1\|COX2\|COX3 |
| KEGG Pathway | ko00190 | Oxidative phosphorylation | -22 | 9.8 | 32 | Atp5g1\|Atp5k\|Cox6a2\|Cox7a1\|ATP6\|ATP8\|COX1\|COX2\|COX3\|CYTB\|ND1\|ND2\|ND3\|ND4\|ND5\|ND6\|Ndufa2\|Uqcrq\|Ndufa1\|Ndufa3\|Uqcr10\|Ndufc1\|Ndufb3\|Uqcrh\|Uqcr11\|Atp5e\|Ndufa5\|Ndufb10\|Atp6v1b1\|Atp5g3\|Ndufb6\|Ndufs6 |
| KEGG Pathway | mmu05012 | Parkinson's disease | -20 | 8.8 | 31 | Slc25a4\|Atp5g1\|Cox6a2\|Cox7a1\|ATP6\|ATP8\|COX1\|COX2\|COX3\|CYTB\|ND1\|ND2\|ND3\|ND4\|ND5\|ND6\|Ndufa2\|Uqcrq\|Ndufa1\|Ndufa3\|Uqcr10\|Ndufc1\|Ndufb3\|Uqcrh\|Uqcr11\|Atp5e\|Ndufa5\|Ndufb10\|Atp5g3\|Ndufb6\|Ndufs6 |
| KEGG Pathway | mmu00190 | Oxidative phosphorylation | -17 | 6.9 | 32 | Atp5g1\|Atp5k\|Cox6a2\|Cox7a1\|ATP6\|ATP8\|COX1\|COX2\|COX3\|CYTB\|ND1\|ND2\|ND3\|ND4\|ND5\|ND6\|Ndufa2\|Uqcrq\|Ndufa1\|Ndufa3\|Uqcr10\|Ndufc1\|Ndufb3\|Uqcrh\|Uqcr11\|Atp5e\|Ndufa5\|Ndufb10\|Atp6v1b1\|Atp5g3\|Ndufb6\|Ndufs6 |
| KEGG Pathway | ko05016 | Huntington's disease | -16 | 6.6 | 31 | Slc25a4\|Atp5g1\|Cox6a2\|Cox7a1\|ATP6\|ATP8\|COX1\|COX2\|COX3\|CYTB\|Ndufa2\|Uqcrq\|Ndufa1\|Dnah10\|Ndufa3\|Uqcr10\|Ndufc1\|Polr2l\|Ndufb3\|Uqcrh\|Uqcr11\|Atp5e\|Ndufa5\|Ndufb10\|Dnai1\|Dnali1\|Atp5g3\|Ndufb6\|Dnah2\|Ndufs6\|Dnah7a |
| KEGG Pathway | mmu05016 | Huntington's disease | -16 | 6.6 | 31 | Slc25a4\|Atp5g1\|Cox6a2\|Cox7a1\|ATP6\|ATP8\|COX1\|COX2\|COX3\|CYTB\|Ndufa2\|Uqcrq\|Ndufa1\|Dnah10\|Ndufa3\|Uqcr10\|Ndufc1\|Polr2l\|Ndufb3\|Uqcrh\|Uqcr11\|Atp5e\|Ndufa5\|Ndufb10\|Dnai1\|Dnali1\|Atp5g3\|Ndufb6\|Dnah2\|Ndufs6\|Dnah7a |
| KEGG Pathway | ko05010 | Alzheimer's disease | -11 | 5.6 | 24 | Atp5g1\|Cox6a2\|Cox7a1\|ATP6\|ATP8\|COX1\|COX2\|COX3\|CYTB\|Ndufa2\|Uqcrq\|Ndufa1\|Ndufa3\|Uqcr10\|Ndufc1\|Ndufb3\|Uqcrh\|Uqcr11\|Atp5e\|Ndufa5\|Ndufb10\|Atp5g3\|Ndufb6\|Ndufs6 |
| KEGG Pathway | mmu05010 | Alzheimer's disease | -11 | 5.6 | 24 | Atp5g1\|Cox6a2\|Cox7a1\|ATP6\|ATP8\|COX1\|COX2\|COX3\|CYTB\|Ndufa2\|Uqcrq\|Ndufa1\|Ndufa3\|Uqcr10\|Ndufc1\|Ndufb3\|Uqcrh\|Uqcr11\|Atp5e\|Ndufa5\|Ndufb10\|Atp5g3\|Ndufb6\|Ndufs6 |
| KEGG Pathway | ko04932 | Non-alcoholic fatty liver disease (NAFLD) | -9.9 | 5.7 | 21 | Adipoq\|Cox6a2\|Cox7a1\|Cyp2e1\|COX1\|COX2\|COX3\|CYTB\|Ndufa2\|Uqcrq\|Ndufa1\|Ndufa3\|Uqcr10\|Ndufc1\|Ndufb3\|Uqcrh\|Uqcr11\|Ndufa5\|Ndufb10\|Ndufb6\|Ndufs6 |
| KEGG Pathway | mmu04932 | Non-alcoholic fatty liver disease (NAFLD) | -8.8 | 5 | 21 | Adipoq\|Cox6a2\|Cox7a1\|Cyp2e1\|COX1\|COX2\|COX3\|CYTB\|Ndufa2\|Uqcrq\|Ndufa1\|Ndufa3\|Uqcr10\|Ndufc1\|Ndufb3\|Uqcrh\|Uqcr11\|Ndufa5\|Ndufb10\|Ndufb6\|Ndufs6 |
| KEGG Pathway | ko04260 | Cardiac muscle contraction | -7.3 | 6.8 | 13 | Cacnb1\|Cox6a2\|Cox7a1\|COX1\|COX2\|COX3\|CYTB\|Tnnc1\|Uqcrq\|Uqcr10\|Uqcrh\|Uqcr11\|Atp1a2 |
| KEGG Pathway | mmu04260 | Cardiac muscle contraction | -6.2 | 5.5 | 13 | Cacnb1\|Cox6a2\|Cox7a1\|COX1\|COX2\|COX3\|CYTB\|Tnnc1\|Uqcrq\|Uqcr10\|Uqcrh\|Uqcr11\|Atp1a2 |
| KEGG Pathway | ko00970 | Aminoacyl-tRNA biosynthesis | -4.5 | 5.6 | 9 | TrnE\|TrnI\|TrnL1\|TrnL2\|TrnN\|TrnP\|TrnS2\|TrnY\|Lars2 |
| KEGG Pathway | mmu00970 | Aminoacyl-tRNA biosynthesis | -4.5 | 5.5 | 9 | TrnE\|TrnI\|TrnL1\|TrnL2\|TrnN\|TrnP\|TrnS2\|TrnY\|Lars2 |
| KEGG Pathway | ko03320 | PPAR signaling pathway | -3.6 | 4.3 | 9 | Adipoq\|Apoa1\|Aqp7\|Fabp3\|Slc27a5\|Plin4\|Plin5\|Plin1\|Acaa1b |
| KEGG Pathway | mmu03320 | PPAR signaling pathway | -3.5 | 4.2 | 9 | Adipoq\|Apoa1\|Aqp7\|Fabp3\|Slc27a5\|Plin4\|Plin5\|Plin1\|Acaa1b |
| KEGG Pathway | ko00590 | Arachidonic acid metabolism | -2.2 | 3.2 | 7 | Cyp2b19\|Cyp2e1\|Ephx2\|Pla2g2c\|Prxl2b\|Cyp2c55\|Pla2g4f |
| KEGG Pathway | mmu04146 | Peroxisome | -2.2 | 3.2 | 7 | Ephx2\|Pipox\|Pxmp2\|Hao2\|Mlycd\|Trpv6\|Acaa1b |
| KEGG Pathway | mmu00590 | Arachidonic acid metabolism | -2.2 | 3.2 | 7 | Cyp2b19\|Cyp2e1\|Ephx2\|Pla2g2c\|Prxl2b\|Cyp2c55\|Pla2g4f |
| KEGG Pathway | ko05204 | Chemical carcinogenesis | -2.1 | 3.1 | 7 | Aldh3a1\|Cyp1a1\|Cyp2b19\|Cyp2e1\|Gsta2\|Cyp2c55\|Mgst2 |
| KEGG Pathway | mmu05204 | Chemical carcinogenesis | -2.1 | 3 | 7 | Aldh3a1\|Cyp1a1\|Cyp2b19\|Cyp2e1\|Gsta2\|Cyp2c55\|Mgst2 |
