# Supplementary material for: Exploring the immune-inflammatory mechanism of Maxing Shigan Decoction in treating influenza virus A-induced pneumonia based on an integrated strategy of single-cell transcriptomics and systems biology
Source: Eur J Med Res. 2024 Apr 15;29:234. doi: 10.1186/s40001-024-01777-9 (PMC11017673; doi:10.1186/s40001-024-01777-9)
Supplement: Supplementary file 3 — Additional file 3: Table S2. Enrichment analysis of down-regulated gene. [file 40001_2024_1777_MOESM3_ESM.docx]

Table S2 Enrichment analysis of Down-regulated Gene

| **Category** | **GO** | **Description** | **LogP** | **Enrichment** | **Counts** | **Genes** |
| --- | --- | --- | --- | --- | --- | --- |
| GO Biological Processes | GO:0002694 | regulation of leukocyte activation | -46 | 5 | 113 | Atm\|B2m\|Blm\|Zfp36l2\|Runx3\|Ccr6\|Cd28\|Cd33\|Cd3e\|Cd4\|Cd5\|Cd6\|Cd84\|Ccr2\|Cst7\|Ctsc\|Egr3\|Fanca\|Fcer1g\|Fcgr2b\|Fgl2\|Lilrb4a\|Ifnb1\|Ifng\|Ighg2b\|Ighg1\|Cd74\|Il12b\|Il12rb1\|Il13ra2\|Il1a\|Il1b\|Il2\|Il2ra\|Il2rg\|Il4\|Il6\|Itgal\|Itgb2\|Klrc1\|Klrc2\|Lck\|Mmp8\|Myd88\|Nr4a3\|Ccl21a\|Ptafr\|Ptpn22\|Ptprc\|Rac2\|Ccl2\|Ccl5\|Foxp3\|Sfrp1\|Sox4\|Thy1\|Tnfaip3\|Traf6\|Ikzf3\|Klrk1\|Icos\|Crtam\|Ripk3\|Clec7a\|Stap1\|Pglyrp2\|Klrc3\|Il21\|Cd274\|Tespa1\|Tnfrsf13c\|Slamf7\|Tlr9\|Nckap1l\|Card11\|Havcr2\|Ighv9-1\|Ighv1-78\|Cgas\|Nlrp3\|Nr1d1\|Cd226\|Tnfrsf14\|Zc3h12d\|Ighv14-3\|Cd300lf\|Rasal3\|Ighg3\|Ighv10-3\|Ighv1-64\|Ighv1-66\|H2-Eb2\|Ighv1-72\|Ighv9-2\|Ighv14-4\|Ighv1-12\|Ighv1-26\|Ighv5-16\|Ighv9-4\|Ighv14-2\|Ighv3-3\|Ighv1-7\|Ighv1-85\|Ighv2-2\|Ighv5-17\|Ighv9-3\|Ighv1-39\|Ighv1-53\|Ighv8-8\|Ighv1-58\|Ighv1-63\|Ighv8-12\|Tigit |
| GO Biological Processes | GO:0050865 | regulation of cell activation | -45 | 4.8 | 115 | Apoe\|Atm\|B2m\|Blm\|Zfp36l2\|Runx3\|Ccr6\|Cd28\|Cd33\|Cd3e\|Cd4\|Cd5\|Cd6\|Cd84\|Ccr2\|Cst7\|Ctsc\|Egr3\|Fanca\|Fcer1g\|Fcgr2b\|Fgl2\|Lilrb4a\|Ifnb1\|Ifng\|Ighg2b\|Ighg1\|Cd74\|Il12b\|Il12rb1\|Il13ra2\|Il1a\|Il1b\|Il2\|Il2ra\|Il2rg\|Il4\|Il6\|Itgal\|Itgb2\|Klrc1\|Klrc2\|Lck\|Mmp8\|Myd88\|Nr4a3\|Ccl21a\|Ptafr\|Ptpn22\|Ptprc\|Rac2\|Ccl2\|Ccl5\|Foxp3\|Sfrp1\|Sox4\|Thy1\|Tnfaip3\|Traf6\|Ikzf3\|Klrk1\|Icos\|Crtam\|Plek\|Ripk3\|Clec7a\|Stap1\|Pglyrp2\|Klrc3\|Il21\|Cd274\|Tespa1\|Tnfrsf13c\|Slamf7\|Tlr9\|Nckap1l\|Card11\|Havcr2\|Ighv9-1\|Ighv1-78\|Cgas\|Nlrp3\|Nr1d1\|Cd226\|Tnfrsf14\|Zc3h12d\|Ighv14-3\|Cd300lf\|Rasal3\|Ighg3\|Ighv10-3\|Ighv1-64\|Ighv1-66\|H2-Eb2\|Ighv1-72\|Ighv9-2\|Ighv14-4\|Ighv1-12\|Ighv1-26\|Ighv5-16\|Ighv9-4\|Ighv14-2\|Ighv3-3\|Ighv1-7\|Ighv1-85\|Ighv2-2\|Ighv5-17\|Ighv9-3\|Ighv1-39\|Ighv1-53\|Ighv8-8\|Ighv1-58\|Ighv1-63\|Ighv8-12\|Tigit |
| GO Biological Processes | GO:0050778 | positive regulation of immune response | -41 | 4.8 | 105 | B2m\|C3ar1\|C5ar1\|Ms4a1\|Cd28\|Cd3e\|Cd4\|Ccr2\|Coch\|Fcer1g\|Fcer2a\|Fcgr3\|Fcnb\|Lilrb4a\|H2-K1\|H2-T24\|Ifi203\|Ifi204\|Ifng\|Ighg2b\|Ighg1\|Cd74\|Il12b\|Il12rb1\|Il1b\|Il2\|Il4\|Il6\|Itgb2\|Itk\|Klrc1\|Klrc2\|Lck\|Nr4a3\|Slc11a1\|Pde4b\|Ptafr\|Ptpn22\|Ptprc\|Foxp3\|Thy1\|Traf6\|Usp9x\|Fyb\|Sh2b2\|Tlr2\|Klrk1\|Elane\|Irf7\|Crtam\|Stap1\|Klrc3\|Zbp1\|Il21\|Cd274\|Tespa1\|Tasl\|Tnfrsf13c\|Sting1\|Skap1\|Tlr9\|Nckap1l\|Lpxn\|Card11\|Tlr8\|Havcr2\|Ighv9-1\|Trim30d\|Themis\|Ighv1-78\|Cgas\|Nlrp3\|Cd226\|Gbp5\|A2m\|Ifi209\|Ighv14-3\|C5ar2\|Ubash3a\|Fcrl5\|Ighg3\|Ighv10-3\|Ighv1-64\|Ighv1-66\|Ifi214\|Ighv1-72\|Ighv9-2\|Ighv14-4\|Ighv1-12\|Ighv1-26\|Ighv5-16\|Ighv9-4\|Ighv14-2\|Ighv3-3\|Ighv1-7\|Ighv1-85\|Ighv2-2\|Ighv5-17\|Ighv9-3\|Ighv1-39\|Ighv1-53\|Ighv8-8\|Ighv1-58\|Ighv1-63\|Ighv8-12 |
| GO Biological Processes | GO:0051249 | regulation of lymphocyte activation | -41 | 5.1 | 98 | Atm\|B2m\|Blm\|Zfp36l2\|Runx3\|Ccr6\|Cd28\|Cd3e\|Cd4\|Cd5\|Cd6\|Ccr2\|Egr3\|Fanca\|Fcgr2b\|Fgl2\|Lilrb4a\|Ifnb1\|Ifng\|Ighg2b\|Ighg1\|Cd74\|Il12b\|Il12rb1\|Il1a\|Il1b\|Il2\|Il2ra\|Il2rg\|Il4\|Il6\|Itgal\|Klrc1\|Klrc2\|Lck\|Myd88\|Ccl21a\|Ptpn22\|Ptprc\|Rac2\|Ccl2\|Ccl5\|Foxp3\|Sfrp1\|Sox4\|Thy1\|Tnfaip3\|Traf6\|Ikzf3\|Icos\|Crtam\|Ripk3\|Clec7a\|Pglyrp2\|Klrc3\|Il21\|Cd274\|Tespa1\|Tnfrsf13c\|Slamf7\|Tlr9\|Nckap1l\|Card11\|Havcr2\|Ighv9-1\|Ighv1-78\|Cgas\|Nlrp3\|Tnfrsf14\|Zc3h12d\|Ighv14-3\|Rasal3\|Ighg3\|Ighv10-3\|Ighv1-64\|Ighv1-66\|H2-Eb2\|Ighv1-72\|Ighv9-2\|Ighv14-4\|Ighv1-12\|Ighv1-26\|Ighv5-16\|Ighv9-4\|Ighv14-2\|Ighv3-3\|Ighv1-7\|Ighv1-85\|Ighv2-2\|Ighv5-17\|Ighv9-3\|Ighv1-39\|Ighv1-53\|Ighv8-8\|Ighv1-58\|Ighv1-63\|Ighv8-12\|Tigit |
| GO Biological Processes | GO:0002696 | positive regulation of leukocyte activation | -41 | 5.7 | 89 | B2m\|Blm\|Runx3\|Cd28\|Cd3e\|Cd4\|Cd5\|Cd6\|Ccr2\|Ctsc\|Egr3\|Fcer1g\|Lilrb4a\|Ifng\|Ighg2b\|Ighg1\|Cd74\|Il12b\|Il12rb1\|Il1a\|Il1b\|Il2\|Il2ra\|Il2rg\|Il4\|Il6\|Itgal\|Itgb2\|Klrc1\|Klrc2\|Lck\|Mmp8\|Myd88\|Nr4a3\|Ccl21a\|Ptafr\|Ptpn22\|Ptprc\|Ccl2\|Ccl5\|Foxp3\|Sox4\|Thy1\|Traf6\|Klrk1\|Icos\|Clec7a\|Stap1\|Klrc3\|Il21\|Cd274\|Tespa1\|Tnfrsf13c\|Tlr9\|Nckap1l\|Card11\|Havcr2\|Ighv9-1\|Ighv1-78\|Nlrp3\|Cd226\|Tnfrsf14\|Ighv14-3\|Rasal3\|Ighg3\|Ighv10-3\|Ighv1-64\|Ighv1-66\|H2-Eb2\|Ighv1-72\|Ighv9-2\|Ighv14-4\|Ighv1-12\|Ighv1-26\|Ighv5-16\|Ighv9-4\|Ighv14-2\|Ighv3-3\|Ighv1-7\|Ighv1-85\|Ighv2-2\|Ighv5-17\|Ighv9-3\|Ighv1-39\|Ighv1-53\|Ighv8-8\|Ighv1-58\|Ighv1-63\|Ighv8-12 |
| GO Biological Processes | GO:0050867 | positive regulation of cell activation | -41 | 5.6 | 90 | B2m\|Blm\|Runx3\|Cd28\|Cd3e\|Cd4\|Cd5\|Cd6\|Ccr2\|Ctsc\|Egr3\|Fcer1g\|Lilrb4a\|Ifng\|Ighg2b\|Ighg1\|Cd74\|Il12b\|Il12rb1\|Il1a\|Il1b\|Il2\|Il2ra\|Il2rg\|Il4\|Il6\|Itgal\|Itgb2\|Klrc1\|Klrc2\|Lck\|Mmp8\|Myd88\|Nr4a3\|Ccl21a\|Ptafr\|Ptpn22\|Ptprc\|Ccl2\|Ccl5\|Foxp3\|Sox4\|Thy1\|Traf6\|Klrk1\|Icos\|Plek\|Clec7a\|Stap1\|Klrc3\|Il21\|Cd274\|Tespa1\|Tnfrsf13c\|Tlr9\|Nckap1l\|Card11\|Havcr2\|Ighv9-1\|Ighv1-78\|Nlrp3\|Cd226\|Tnfrsf14\|Ighv14-3\|Rasal3\|Ighg3\|Ighv10-3\|Ighv1-64\|Ighv1-66\|H2-Eb2\|Ighv1-72\|Ighv9-2\|Ighv14-4\|Ighv1-12\|Ighv1-26\|Ighv5-16\|Ighv9-4\|Ighv14-2\|Ighv3-3\|Ighv1-7\|Ighv1-85\|Ighv2-2\|Ighv5-17\|Ighv9-3\|Ighv1-39\|Ighv1-53\|Ighv8-8\|Ighv1-58\|Ighv1-63\|Ighv8-12 |
| GO Biological Processes | GO:0002250 | adaptive immune response | -39 | 4.9 | 96 | B2m\|C3ar1\|Camk4\|Ccr6\|Cd28\|Cd3e\|Cd3g\|Cd4\|Cd48\|Cd84\|Cd8a\|Cd8b1\|Ccr2\|Ctsc\|Ctsh\|Eomes\|Fcer1g\|Fcer2a\|Fcgr2b\|Fcgr3\|Fgl2\|Fut7\|Lilrb4a\|Gzmb\|H2-K1\|H2-T24\|Icam1\|Ifnb1\|Ifng\|Ighg2b\|Ighg1\|Cd74\|Il12b\|Il12rb1\|Il13ra2\|Il1b\|Il2\|Il4\|Il6\|Itk\|Ly9\|Myd88\|Slc11a1\|Prf1\|Ptprc\|Foxp3\|Serpinb9b\|Serpina3g\|Tnfaip3\|Traf6\|Was\|Klrk1\|Irf7\|Crtam\|Ripk3\|Cd274\|Rnf125\|Mcoln2\|Tnfrsf13c\|Slamf7\|Skap1\|Nckap1l\|Iglv2\|Havcr2\|Ighv9-1\|Themis\|Ighv1-78\|Nlrp3\|Cd226\|Tnfrsf14\|Ighv14-3\|Ighg3\|Ighv10-3\|Ighv1-64\|Ighv1-66\|H2-Eb2\|Ighv1-72\|Ighv9-2\|Ighv14-4\|Ighv1-12\|Ighv1-26\|Ighv5-16\|Ighv9-4\|Ighv14-2\|Ighv3-3\|Ighv1-7\|Ighv1-85\|Ighv2-2\|Ighv5-17\|Ighv9-3\|Ighv1-39\|Ighv1-53\|Ighv8-8\|Ighv1-58\|Ighv1-63\|Ighv8-12 |
| GO Biological Processes | GO:0002443 | leukocyte mediated immunity | -38 | 5 | 93 | B2m\|Ccr6\|Cd28\|Cd84\|Cd8a\|Ccr2\|Ctsc\|Ctsh\|Dnase1l3\|Fcer1g\|Fcer2a\|Fcgr2b\|Fcgr3\|Fgl2\|Fut7\|Lilrb4a\|Gzmb\|H2-K1\|H2-T24\|Icam1\|Ifnb1\|Ifng\|Ighg2b\|Ighg1\|Cd74\|Il12b\|Il13ra2\|Il1b\|Il2\|Il4\|Il6\|Itgb2\|Klrc1\|Klrc2\|Myd88\|Myo1f\|Nr4a3\|Slc11a1\|Prf1\|Ptafr\|Ptprc\|Rac2\|Ccl2\|Foxp3\|Serpinb9b\|Traf6\|Was\|Tlr2\|Klrk1\|Elane\|Irf7\|Crtam\|Ripk3\|Stap1\|Klrc3\|Il21\|Tlr9\|Cd96\|Spon2\|Nckap1l\|Havcr2\|Ighv9-1\|Ighv1-78\|Nlrp3\|Cd226\|Ighv14-3\|Scimp\|Ighg3\|Ighv10-3\|Ighv1-64\|Ighv1-66\|H2-Eb2\|Ighv1-72\|Ighv9-2\|Ighv14-4\|Ighv1-12\|Ighv1-26\|Ighv5-16\|Ighv9-4\|Ighv14-2\|Ighv3-3\|Ighv1-7\|Ighv1-85\|H60c\|Ighv2-2\|Ighv5-17\|Ighv9-3\|Ighv1-39\|Ighv1-53\|Ighv8-8\|Ighv1-58\|Ighv1-63\|Ighv8-12 |
| GO Biological Processes | GO:0051251 | positive regulation of lymphocyte activation | -38 | 5.8 | 80 | B2m\|Blm\|Runx3\|Cd28\|Cd3e\|Cd4\|Cd5\|Cd6\|Ccr2\|Egr3\|Lilrb4a\|Ifng\|Ighg2b\|Ighg1\|Cd74\|Il12b\|Il12rb1\|Il1a\|Il1b\|Il2\|Il2ra\|Il2rg\|Il4\|Il6\|Itgal\|Klrc1\|Klrc2\|Lck\|Myd88\|Ccl21a\|Ptpn22\|Ptprc\|Ccl2\|Ccl5\|Foxp3\|Sox4\|Thy1\|Traf6\|Icos\|Clec7a\|Klrc3\|Il21\|Cd274\|Tespa1\|Tnfrsf13c\|Tlr9\|Nckap1l\|Card11\|Havcr2\|Ighv9-1\|Ighv1-78\|Nlrp3\|Tnfrsf14\|Ighv14-3\|Rasal3\|Ighg3\|Ighv10-3\|Ighv1-64\|Ighv1-66\|H2-Eb2\|Ighv1-72\|Ighv9-2\|Ighv14-4\|Ighv1-12\|Ighv1-26\|Ighv5-16\|Ighv9-4\|Ighv14-2\|Ighv3-3\|Ighv1-7\|Ighv1-85\|Ighv2-2\|Ighv5-17\|Ighv9-3\|Ighv1-39\|Ighv1-53\|Ighv8-8\|Ighv1-58\|Ighv1-63\|Ighv8-12 |
| GO Biological Processes | GO:0002764 | immune response-regulating signaling pathway | -37 | 5.3 | 85 | C3ar1\|C5ar1\|Ms4a1\|Cd28\|Cd33\|Cd3e\|Ctsh\|Fcer1g\|Lilrb4a\|Ifng\|Ighg2b\|Ighg1\|Itk\|Klrc1\|Klrc2\|Lck\|Cd180\|Myd88\|Nr4a3\|Pde4b\|Ptpn22\|Ptprc\|Foxp3\|Thy1\|Tnfaip3\|Traf6\|Usp9x\|Fyb\|Sh2b2\|Tlr2\|Klrk1\|Irf7\|Clec4e\|Stap1\|Klrc3\|Slc15a3\|Tespa1\|Rnf125\|Tasl\|Skap1\|Tlr9\|Pik3ap1\|Nckap1l\|Lpxn\|Card11\|Tlr7\|Tlr8\|Havcr2\|Ighv9-1\|Themis\|Lacc1\|Ighv1-78\|Nr1d1\|Cd226\|Ighv14-3\|Oas3\|Cd300lf\|C5ar2\|Scimp\|Ubash3a\|Fcrl5\|Ighg3\|Ighv10-3\|Ighv1-64\|Ighv1-66\|Ighv1-72\|Ighv9-2\|Ighv14-4\|Ighv1-12\|Ighv1-26\|Ighv5-16\|Ighv9-4\|Ighv14-2\|Ighv3-3\|Ighv1-7\|Ighv1-85\|Ighv2-2\|Ighv5-17\|Ighv9-3\|Ighv1-39\|Ighv1-53\|Ighv8-8\|Ighv1-58\|Ighv1-63\|Ighv8-12 |
| GO Biological Processes | GO:0002253 | activation of immune response | -35 | 5.6 | 77 | C3ar1\|C5ar1\|Ms4a1\|Cd28\|Cd3e\|Fcer1g\|Fcnb\|Lilrb4a\|Ifi203\|Ifi204\|Ifng\|Ighg2b\|Ighg1\|Il1b\|Itk\|Klrc1\|Klrc2\|Lck\|Nr4a3\|Pde4b\|Ptpn22\|Ptprc\|Foxp3\|Thy1\|Traf6\|Usp9x\|Fyb\|Sh2b2\|Tlr2\|Klrk1\|Stap1\|Klrc3\|Zbp1\|Tespa1\|Sting1\|Skap1\|Tlr9\|Nckap1l\|Lpxn\|Card11\|Ighv9-1\|Trim30d\|Themis\|Ighv1-78\|Cgas\|Cd226\|A2m\|Ifi209\|Ighv14-3\|C5ar2\|Ubash3a\|Fcrl5\|Ighg3\|Ighv10-3\|Ighv1-64\|Ighv1-66\|Ifi214\|Ighv1-72\|Ighv9-2\|Ighv14-4\|Ighv1-12\|Ighv1-26\|Ighv5-16\|Ighv9-4\|Ighv14-2\|Ighv3-3\|Ighv1-7\|Ighv1-85\|Ighv2-2\|Ighv5-17\|Ighv9-3\|Ighv1-39\|Ighv1-53\|Ighv8-8\|Ighv1-58\|Ighv1-63\|Ighv8-12 |
| GO Biological Processes | GO:0002449 | lymphocyte mediated immunity | -34 | 5.3 | 79 | B2m\|Ccr6\|Cd28\|Cd8a\|Ccr2\|Ctsc\|Ctsh\|Fcer1g\|Fcer2a\|Fcgr2b\|Fcgr3\|Fgl2\|Fut7\|Lilrb4a\|Gzmb\|H2-K1\|H2-T24\|Icam1\|Ifnb1\|Ifng\|Ighg2b\|Ighg1\|Cd74\|Il12b\|Il13ra2\|Il1b\|Il2\|Il4\|Il6\|Klrc1\|Klrc2\|Myd88\|Slc11a1\|Prf1\|Ptprc\|Foxp3\|Serpinb9b\|Traf6\|Was\|Klrk1\|Irf7\|Crtam\|Ripk3\|Klrc3\|Il21\|Cd96\|Nckap1l\|Havcr2\|Ighv9-1\|Ighv1-78\|Nlrp3\|Cd226\|Ighv14-3\|Ighg3\|Ighv10-3\|Ighv1-64\|Ighv1-66\|H2-Eb2\|Ighv1-72\|Ighv9-2\|Ighv14-4\|Ighv1-12\|Ighv1-26\|Ighv5-16\|Ighv9-4\|Ighv14-2\|Ighv3-3\|Ighv1-7\|Ighv1-85\|H60c\|Ighv2-2\|Ighv5-17\|Ighv9-3\|Ighv1-39\|Ighv1-53\|Ighv8-8\|Ighv1-58\|Ighv1-63\|Ighv8-12 |
| GO Biological Processes | GO:0002460 | adaptive immune response based on somatic recombination of immune receptors built from immunoglobulin superfamily domains | -33 | 5.1 | 78 | B2m\|C3ar1\|Ccr6\|Cd28\|Cd4\|Cd8a\|Ccr2\|Ctsc\|Ctsh\|Fcer1g\|Fcer2a\|Fcgr2b\|Fcgr3\|Fgl2\|Fut7\|Lilrb4a\|Gzmb\|H2-K1\|H2-T24\|Icam1\|Ifnb1\|Ifng\|Ighg2b\|Ighg1\|Cd74\|Il12b\|Il12rb1\|Il13ra2\|Il1b\|Il2\|Il4\|Il6\|Ly9\|Myd88\|Slc11a1\|Prf1\|Ptprc\|Foxp3\|Serpinb9b\|Tnfaip3\|Traf6\|Was\|Irf7\|Ripk3\|Cd274\|Tnfrsf13c\|Nckap1l\|Havcr2\|Ighv9-1\|Ighv1-78\|Nlrp3\|Cd226\|Ighv14-3\|Ighg3\|Ighv10-3\|Ighv1-64\|Ighv1-66\|H2-Eb2\|Ighv1-72\|Ighv9-2\|Ighv14-4\|Ighv1-12\|Ighv1-26\|Ighv5-16\|Ighv9-4\|Ighv14-2\|Ighv3-3\|Ighv1-7\|Ighv1-85\|Ighv2-2\|Ighv5-17\|Ighv9-3\|Ighv1-39\|Ighv1-53\|Ighv8-8\|Ighv1-58\|Ighv1-63\|Ighv8-12 |
| GO Biological Processes | GO:0042110 | T cell activation | -33 | 4.8 | 83 | Chrna7\|Atp7a\|B2m\|Blm\|Zfp36l2\|Runx2\|Runx3\|Ccr6\|Cd28\|Cd3e\|Cd3g\|Cd4\|Cd48\|Cd5\|Cd6\|Cd84\|Cd8a\|Ccr9\|Ccr2\|Egr3\|Eomes\|Fanca\|Fcer1g\|Fgl2\|Fut7\|Lilrb4a\|Icam1\|Ifnb1\|Ifng\|Cd74\|Il12b\|Il12rb1\|Il1a\|Il1b\|Il2\|Il2ra\|Il2rg\|Il4\|Il6\|Itgal\|Itgb2\|Itk\|Lck\|Ly9\|Myh9\|Slc11a1\|Lcp1\|Ccl21a\|Ptpn22\|Ptprc\|Rac2\|Ccl2\|Ccl5\|Foxp3\|Sox4\|Thy1\|Traf6\|Was\|Icos\|Crtam\|Elf4\|Ripk3\|Clec4e\|Il21\|Cd274\|Tespa1\|Tnfrsf13c\|Slamf7\|Dock2\|Nckap1l\|Card11\|Havcr2\|Themis\|Cgas\|Nlrp3\|Tnfrsf14\|Zc3h12d\|Nlrc3\|Jaml\|Rasal3\|H2-Eb2\|Itgad\|Tigit |
| GO Biological Processes | GO:0002768 | immune response-regulating cell surface receptor signaling pathway | -32 | 6 | 66 | C3ar1\|C5ar1\|Ms4a1\|Cd28\|Cd3e\|Fcer1g\|Lilrb4a\|Ifng\|Ighg2b\|Ighg1\|Itk\|Klrc1\|Klrc2\|Lck\|Nr4a3\|Pde4b\|Ptpn22\|Ptprc\|Foxp3\|Thy1\|Traf6\|Usp9x\|Fyb\|Sh2b2\|Tlr2\|Klrk1\|Clec4e\|Stap1\|Klrc3\|Tespa1\|Skap1\|Nckap1l\|Lpxn\|Card11\|Ighv9-1\|Themis\|Ighv1-78\|Cd226\|Ighv14-3\|C5ar2\|Ubash3a\|Fcrl5\|Ighg3\|Ighv10-3\|Ighv1-64\|Ighv1-66\|Ighv1-72\|Ighv9-2\|Ighv14-4\|Ighv1-12\|Ighv1-26\|Ighv5-16\|Ighv9-4\|Ighv14-2\|Ighv3-3\|Ighv1-7\|Ighv1-85\|Ighv2-2\|Ighv5-17\|Ighv9-3\|Ighv1-39\|Ighv1-53\|Ighv8-8\|Ighv1-58\|Ighv1-63\|Ighv8-12 |
| GO Biological Processes | GO:0002429 | immune response-activating cell surface receptor signaling pathway | -32 | 6.1 | 65 | C3ar1\|C5ar1\|Ms4a1\|Cd28\|Cd3e\|Fcer1g\|Lilrb4a\|Ifng\|Ighg2b\|Ighg1\|Itk\|Klrc1\|Klrc2\|Lck\|Nr4a3\|Pde4b\|Ptpn22\|Ptprc\|Foxp3\|Thy1\|Traf6\|Usp9x\|Fyb\|Sh2b2\|Tlr2\|Klrk1\|Stap1\|Klrc3\|Tespa1\|Skap1\|Nckap1l\|Lpxn\|Card11\|Ighv9-1\|Themis\|Ighv1-78\|Cd226\|Ighv14-3\|C5ar2\|Ubash3a\|Fcrl5\|Ighg3\|Ighv10-3\|Ighv1-64\|Ighv1-66\|Ighv1-72\|Ighv9-2\|Ighv14-4\|Ighv1-12\|Ighv1-26\|Ighv5-16\|Ighv9-4\|Ighv14-2\|Ighv3-3\|Ighv1-7\|Ighv1-85\|Ighv2-2\|Ighv5-17\|Ighv9-3\|Ighv1-39\|Ighv1-53\|Ighv8-8\|Ighv1-58\|Ighv1-63\|Ighv8-12 |
| GO Biological Processes | GO:0002757 | immune response-activating signal transduction | -32 | 6.1 | 65 | C3ar1\|C5ar1\|Ms4a1\|Cd28\|Cd3e\|Fcer1g\|Lilrb4a\|Ifng\|Ighg2b\|Ighg1\|Itk\|Klrc1\|Klrc2\|Lck\|Nr4a3\|Pde4b\|Ptpn22\|Ptprc\|Foxp3\|Thy1\|Traf6\|Usp9x\|Fyb\|Sh2b2\|Tlr2\|Klrk1\|Stap1\|Klrc3\|Tespa1\|Skap1\|Nckap1l\|Lpxn\|Card11\|Ighv9-1\|Themis\|Ighv1-78\|Cd226\|Ighv14-3\|C5ar2\|Ubash3a\|Fcrl5\|Ighg3\|Ighv10-3\|Ighv1-64\|Ighv1-66\|Ighv1-72\|Ighv9-2\|Ighv14-4\|Ighv1-12\|Ighv1-26\|Ighv5-16\|Ighv9-4\|Ighv14-2\|Ighv3-3\|Ighv1-7\|Ighv1-85\|Ighv2-2\|Ighv5-17\|Ighv9-3\|Ighv1-39\|Ighv1-53\|Ighv8-8\|Ighv1-58\|Ighv1-63\|Ighv8-12 |
| GO Biological Processes | GO:0001819 | positive regulation of cytokine production | -31 | 4.9 | 77 | Agtr2\|B2m\|C3ar1\|C5ar1\|Cd28\|Cd3e\|Cd6\|Cd84\|Clu\|Ccr2\|Camp\|F2r\|Fcer1g\|Fcgr3\|Hgf\|Hpse\|Ifi204\|Ifng\|Cd74\|Il12b\|Il12rb1\|Il12rb2\|Il1a\|Il1b\|Il2\|Il4\|Il6\|Itk\|Ly9\|Mmp8\|Myd88\|Nr4a3\|Slc11a1\|Pde4b\|Serpine1\|Pou2af1\|Ptafr\|Ptpn22\|Ptprc\|Ccl2\|Ccl3\|Ccl4\|Ccl5\|Foxp3\|Tnfrsf8\|Traf6\|Clec5a\|Tlr2\|Klrk1\|Elane\|Irf7\|Crtam\|Clec4e\|Clec7a\|Il21\|Cd274\|Mcoln2\|Tnfrsf13c\|Sting1\|Tlr9\|Spon2\|Card11\|Tlr7\|Tlr8\|Havcr2\|Lacc1\|Cgas\|Nlrp3\|Tmem106a\|Cd226\|Afap1l2\|Gbp5\|Tnfrsf14\|Oas3\|Tnfsf15\|Scimp\|Tigit |
| GO Biological Processes | GO:1903039 | positive regulation of leukocyte cell-cell adhesion | -30 | 7.2 | 54 | B2m\|Blm\|Runx3\|Cd28\|Cd3e\|Cd4\|Cd5\|Cd6\|Ccr2\|Egr3\|Fut7\|Lilrb4a\|Has2\|Icam1\|Ifng\|Cd74\|Il12b\|Il12rb1\|Il1a\|Il1b\|Il2\|Il2ra\|Il2rg\|Il4\|Il6\|Itga4\|Itgal\|Itgb2\|Lck\|Nr4a3\|Ccl21a\|Ptafr\|Ptpn22\|Ptprc\|Ccl2\|Ccl5\|Foxp3\|Sox4\|Thy1\|Traf6\|Elane\|Icos\|Il21\|Cd274\|Tespa1\|Tnfrsf13c\|Skap1\|Nckap1l\|Card11\|Havcr2\|Nlrp3\|Tnfrsf14\|Rasal3\|H2-Eb2 |
| GO Biological Processes | GO:0001817 | regulation of cytokine production | -29 | 3.9 | 94 | Chrna7\|Agtr2\|B2m\|C3ar1\|C5ar1\|Cd28\|Cd33\|Cd3e\|Cd6\|Cd84\|Clu\|Ccr2\|Camp\|F2r\|Fcer1g\|Fcgr2b\|Fcgr3\|Lilrb4a\|Hgf\|Hpse\|Ifi204\|Ifnb1\|Ifng\|Cd74\|Il12b\|Il12rb1\|Il12rb2\|Il1a\|Il1b\|Il2\|Il4\|Il6\|Itk\|Ly9\|Mmp8\|Myd88\|Nr4a3\|Slc11a1\|Pde4b\|Serpine1\|Pou2af1\|Ptafr\|Ptpn22\|Ptprc\|Ccl2\|Ccl3\|Ccl4\|Ccl5\|Foxp3\|Tnfaip3\|Tnfrsf8\|Traf6\|Traip\|Clec5a\|Tlr2\|Klrk1\|Elane\|Irf7\|Mefv\|Crtam\|Ripk3\|Clec4e\|Clec7a\|Pglyrp2\|Il21\|Cd274\|Rnf125\|Mcoln2\|Tnfrsf13c\|Sting1\|Muc16\|Tlr9\|Cd96\|Spon2\|Nckap1l\|Card11\|Tlr7\|Tlr8\|Havcr2\|Lacc1\|Cgas\|Nlrp3\|Tmem106a\|Cd226\|Afap1l2\|Gbp5\|Tnfrsf14\|Oas3\|Nlrc3\|C5ar2\|Tnfsf15\|Scimp\|Ubash3a\|Tigit |
| GO Cellular Components | GO:0009897 | external side of plasma membrane | -42 | 5.1 | 100 | Chrna7\|Adam19\|Apoe\|B2m\|Cxcr5\|Ccr6\|Ms4a1\|Cd28\|Cd33\|Cd3e\|Cd3g\|Cd4\|Cd48\|Cd5\|Cd6\|Cd84\|Cd8a\|Cd8b1\|Cxcr2\|Cxcr3\|Ccr1\|Ccr9\|Ccr2\|Ccr8\|Csf3r\|Fasl\|Fcer1g\|Fcer2a\|Fcgr2b\|Fcgr3\|Fcnb\|Lilrb4a\|H2-K1\|H2-T24\|Icam1\|Ifng\|Ighg2b\|Ighg1\|Cd74\|Il12b\|Il12rb1\|Il12rb2\|Il13ra2\|Il2ra\|Il2rb\|Il2rg\|Il4\|Il6\|Itga4\|Itgal\|Itgb2\|Klrc1\|Klrc2\|Lhcgr\|Ly9\|Ptprc\|Trgv2\|Thy1\|Tlr2\|Klrk1\|Icos\|Ccrl2\|Klrc3\|Cd274\|Tnfrsf13c\|Muc16\|Slamf7\|Trgc2\|Tlr8\|Ighv9-1\|Ighv1-78\|Cd226\|Tnfrsf14\|Ighv14-3\|Ighg3\|Ighv10-3\|Ighv1-64\|Ighv1-66\|H2-Eb2\|Ighv1-72\|Ighv9-2\|Ighv14-4\|Ighv1-12\|Ighv1-26\|Ighv5-16\|Ighv9-4\|Ighv14-2\|Ighv3-3\|Ighv1-7\|Ighv1-85\|H60c\|Ighv2-2\|Ighv5-17\|Ighv9-3\|Ighv1-39\|Ighv1-53\|Ighv8-8\|Ighv1-58\|Ighv1-63\|Ighv8-12 |
| GO Cellular Components | GO:0042571 | immunoglobulin complex, circulating | -15 | 6.6 | 29 | Ighg2b\|Ighg1\|Ighv9-1\|Ighv1-78\|Ighv14-3\|Ighg3\|Ighv10-3\|Ighv1-64\|Ighv1-66\|Ighv1-72\|Ighv9-2\|Ighv14-4\|Ighv1-12\|Ighv1-26\|Ighv5-16\|Ighv9-4\|Ighv14-2\|Ighv3-3\|Ighv1-7\|Ighv1-85\|Ighv2-2\|Ighv5-17\|Ighv9-3\|Ighv1-39\|Ighv1-53\|Ighv8-8\|Ighv1-58\|Ighv1-63\|Ighv8-12 |
| GO Cellular Components | GO:0019814 | immunoglobulin complex | -15 | 6.4 | 30 | Ighg2b\|Ighg1\|Iglv2\|Ighv9-1\|Ighv1-78\|Ighv14-3\|Ighg3\|Ighv10-3\|Ighv1-64\|Ighv1-66\|Ighv1-72\|Ighv9-2\|Ighv14-4\|Ighv1-12\|Ighv1-26\|Ighv5-16\|Ighv9-4\|Ighv14-2\|Ighv3-3\|Ighv1-7\|Ighv1-85\|Ighv2-2\|Ighv5-17\|Ighv9-3\|Ighv1-39\|Ighv1-53\|Ighv8-8\|Ighv1-58\|Ighv1-63\|Ighv8-12 |
| GO Cellular Components | GO:0001772 | immunological synapse | -10 | 9.7 | 14 | Cd28\|Cd3e\|Cd53\|Cd6\|Icam1\|Itgal\|Lck\|Myh9\|Crtam\|Skap1\|Card11\|Havcr2\|Scimp\|H2-Eb2 |
| GO Cellular Components | GO:0043235 | receptor complex | -6.6 | 2.6 | 34 | Chrna7\|B2m\|Cd3e\|Cd3g\|Cd6\|Csf3r\|Gabra3\|Cd74\|Il12b\|Il12rb1\|Il12rb2\|Il13ra2\|Il2rg\|Il6\|Irs1\|Itga4\|Itgal\|Itgb2\|Klrc1\|Klrc2\|Lhcgr\|Lrp8\|Traf6\|Tlr2\|Klrc3\|Loxl4\|Skap1\|Gpr63\|Sacm1l\|Tlr7\|Scimp\|Fcrl5\|Shisa6\|Itgad |
| GO Cellular Components | GO:0030496 | midbody | -5.3 | 3.3 | 19 | Anxa11\|Aspm\|Capg\|Hspa5\|Kif4\|Kif20a\|Cdca8\|Septin1\|Anln\|Kif23\|Cep55\|Cenpf\|Agap2\|Cenpe\|Prc1\|Kif20b\|Vps37b\|Kif14\|Urb2 |
| GO Cellular Components | GO:0005819 | spindle | -5.2 | 2.6 | 27 | Anxa11\|Atm\|Bub1b\|Aspm\|Capg\|Cxcr2\|Kif11\|Myh9\|Kif20a\|Tubgcp4\|Cdca8\|Septin1\|Haus2\|Kif18b\|Ckap2l\|Kif23\|Tpx2\|Espl1\|Cenpf\|Nusap1\|Dlgap5\|Cenpe\|Prc1\|Kif20b\|Ccnb1\|Ptpn7\|Kif14 |
| GO Cellular Components | GO:0045121 | membrane raft | -5.1 | 2.4 | 30 | Chrna7\|Atp7a\|Ms4a1\|Cd28\|Cd4\|Cd48\|Cd8a\|F2r\|Fasl\|Fcer1g\|Fcgr2b\|Has2\|Hpse\|Hspa1b\|Icam1\|Irs1\|Itgb2\|Lck\|Lrp8\|Ptprc\|Rgs16\|Selplg\|Thy1\|Vcl\|Tlr2\|Ms4a4b\|Nos1ap\|Skap1\|Card11\|Hspa1a |
| GO Cellular Components | GO:0098857 | membrane microdomain | -5.1 | 2.4 | 30 | Chrna7\|Atp7a\|Ms4a1\|Cd28\|Cd4\|Cd48\|Cd8a\|F2r\|Fasl\|Fcer1g\|Fcgr2b\|Has2\|Hpse\|Hspa1b\|Icam1\|Irs1\|Itgb2\|Lck\|Lrp8\|Ptprc\|Rgs16\|Selplg\|Thy1\|Vcl\|Tlr2\|Ms4a4b\|Nos1ap\|Skap1\|Card11\|Hspa1a |
| GO Cellular Components | GO:0020005 | symbiont-containing vacuole membrane | -4.5 | 18 | 4 | Gbp2\|Iigp1\|Gbp6\|Gbp9 |
| GO Cellular Components | GO:0098802 | plasma membrane signaling receptor complex | -4.3 | 3 | 17 | Chrna7\|B2m\|Cd3e\|Cd3g\|Cd6\|Il12rb1\|Il6\|Irs1\|Itga4\|Itgal\|Itgb2\|Traf6\|Tlr2\|Skap1\|Sacm1l\|Shisa6\|Itgad |
| GO Cellular Components | GO:0045335 | phagocytic vesicle | -4.3 | 3.7 | 13 | Anxa11\|B2m\|Capg\|Cybb\|H2-K1\|Slc11a1\|Was\|Sec61a1\|Clec4e\|Fmnl1\|Tlr9\|Tlr7\|Scimp |
| GO Cellular Components | GO:0000796 | condensin complex | -4.2 | 16 | 4 | Smc2\|Ncapg\|Ncapd2\|Ncapg2 |
| GO Cellular Components | GO:0033646 | host intracellular part | -4 | 14 | 4 | Gbp2\|Iigp1\|Gbp6\|Gbp9 |
| GO Cellular Components | GO:0033655 | host cell cytoplasm part | -4 | 14 | 4 | Gbp2\|Iigp1\|Gbp6\|Gbp9 |
| GO Cellular Components | GO:0030430 | host cell cytoplasm | -4 | 14 | 4 | Gbp2\|Iigp1\|Gbp6\|Gbp9 |
| GO Cellular Components | GO:0020003 | symbiont-containing vacuole | -4 | 14 | 4 | Gbp2\|Iigp1\|Gbp6\|Gbp9 |
| GO Cellular Components | GO:0043656 | host intracellular region | -4 | 14 | 4 | Gbp2\|Iigp1\|Gbp6\|Gbp9 |
| GO Cellular Components | GO:0000323 | lytic vacuole | -3.8 | 2 | 33 | Anxa11\|Apoe\|Arsb\|Cd68\|Cxcr2\|Cst7\|Ctsc\|Ctsh\|Ctsw\|Fasl\|Gzmb\|H2-K1\|Hexb\|Hpse\|Cd74\|Il1b\|Lhcgr\|Mpo\|Slc11a1\|Prf1\|Tnfaip3\|Ncoa4\|Trpm2\|Slc15a3\|Mcoln2\|Slc29a3\|Tasl\|Tlr9\|Tm6sf1\|Tlr7\|Dtx3l\|Cyb561a3\|H2-Eb2 |
| GO Cellular Components | GO:0005764 | lysosome | -3.8 | 2 | 33 | Anxa11\|Apoe\|Arsb\|Cd68\|Cxcr2\|Cst7\|Ctsc\|Ctsh\|Ctsw\|Fasl\|Gzmb\|H2-K1\|Hexb\|Hpse\|Cd74\|Il1b\|Lhcgr\|Mpo\|Slc11a1\|Prf1\|Tnfaip3\|Ncoa4\|Trpm2\|Slc15a3\|Mcoln2\|Slc29a3\|Tasl\|Tlr9\|Tm6sf1\|Tlr7\|Dtx3l\|Cyb561a3\|H2-Eb2 |
| GO Molecular Functions | GO:0140375 | immune receptor activity | -23 | 8.4 | 36 | Cxcr5\|C3ar1\|C5ar1\|Ccr6\|Cd4\|Cxcr2\|Cxcr3\|Ccr1\|Ccr9\|Ccr2\|Ccr8\|Csf2rb\|Csf2rb2\|Csf3r\|Ctsh\|Fcer1g\|Fcgr2b\|Fcgr3\|Ifnar2\|Cd74\|Il12b\|Il12rb1\|Il12rb2\|Il13ra2\|Il2ra\|Il2rb\|Il2rg\|Klrc1\|Klrc2\|Klrk1\|Ccrl2\|Klrc3\|Il21r\|Il22ra2\|C5ar2\|H2-Eb2 |
| GO Molecular Functions | GO:0003823 | antigen binding | -19 | 5.7 | 41 | Cd48\|Fcnb\|H2-K1\|H2-T24\|Ighg2b\|Ighg1\|Itga4\|Klrc1\|Klrc2\|Lck\|Klrc3\|Dhcr24\|Spon2\|Ighv9-1\|Ighv1-78\|Ighv14-3\|Ighg3\|Ighv10-3\|Ighv1-64\|Ighv1-66\|H2-Eb2\|Ighv1-72\|Ighv9-2\|Ighv14-4\|Ighv1-12\|Ighv1-26\|Ighv5-16\|Ighv9-4\|Ighv14-2\|Ighv3-3\|Ighv1-7\|Ighv1-85\|Ighv2-2\|Ighv5-17\|Ighv9-3\|Ighv1-39\|Ighv1-53\|Ighv8-8\|Ighv1-58\|Ighv1-63\|Ighv8-12 |
| GO Molecular Functions | GO:0034987 | immunoglobulin receptor binding | -15 | 6.5 | 29 | Ighg2b\|Ighg1\|Ighv9-1\|Ighv1-78\|Ighv14-3\|Ighg3\|Ighv10-3\|Ighv1-64\|Ighv1-66\|Ighv1-72\|Ighv9-2\|Ighv14-4\|Ighv1-12\|Ighv1-26\|Ighv5-16\|Ighv9-4\|Ighv14-2\|Ighv3-3\|Ighv1-7\|Ighv1-85\|Ighv2-2\|Ighv5-17\|Ighv9-3\|Ighv1-39\|Ighv1-53\|Ighv8-8\|Ighv1-58\|Ighv1-63\|Ighv8-12 |
| GO Molecular Functions | GO:0004896 | cytokine receptor activity | -15 | 7.9 | 24 | Cxcr5\|Ccr6\|Cd4\|Cxcr2\|Cxcr3\|Ccr1\|Ccr9\|Ccr2\|Ccr8\|Csf2rb\|Csf2rb2\|Csf3r\|Ifnar2\|Cd74\|Il12b\|Il12rb1\|Il12rb2\|Il13ra2\|Il2ra\|Il2rb\|Il2rg\|Ccrl2\|Il21r\|Il22ra2 |
| GO Molecular Functions | GO:0019955 | cytokine binding | -14 | 6.4 | 28 | Cxcr5\|Ccr6\|Cd4\|Cxcr2\|Cxcr3\|Ccr1\|Ccr9\|Ccr2\|Ccr8\|Csf3r\|Gbp2\|Ifnar2\|Cd74\|Il12b\|Il12rb1\|Il12rb2\|Il13ra2\|Il1rn\|Il2ra\|Il2rb\|Il2rg\|Itga4\|Elane\|Ccrl2\|Tnfrsf14\|A2m\|Cd109\|Il22ra2 |
| GO Molecular Functions | GO:0019956 | chemokine binding | -8.4 | 10 | 11 | Cxcr5\|Ccr6\|Cxcr2\|Cxcr3\|Ccr1\|Ccr9\|Ccr2\|Ccr8\|Itga4\|Ccrl2\|A2m |
| GO Molecular Functions | GO:0038187 | pattern recognition receptor activity | -8 | 11 | 10 | Fcnb\|Ptafr\|Tlr2\|Clec4e\|Clec7a\|Pglyrp2\|Tlr9\|Tlr7\|Tlr8\|Trim30d |
| GO Molecular Functions | GO:0016493 | C-C chemokine receptor activity | -7.6 | 12 | 9 | Cxcr5\|Ccr6\|Cxcr2\|Cxcr3\|Ccr1\|Ccr9\|Ccr2\|Ccr8\|Ccrl2 |
| GO Molecular Functions | GO:0019957 | C-C chemokine binding | -7.4 | 11 | 9 | Cxcr5\|Ccr6\|Cxcr2\|Cxcr3\|Ccr1\|Ccr9\|Ccr2\|Ccr8\|Ccrl2 |
| GO Molecular Functions | GO:0004950 | chemokine receptor activity | -7.4 | 11 | 9 | Cxcr5\|Ccr6\|Cxcr2\|Cxcr3\|Ccr1\|Ccr9\|Ccr2\|Ccr8\|Ccrl2 |
| GO Molecular Functions | GO:0001637 | G protein-coupled chemoattractant receptor activity | -7.4 | 11 | 9 | Cxcr5\|Ccr6\|Cxcr2\|Cxcr3\|Ccr1\|Ccr9\|Ccr2\|Ccr8\|Ccrl2 |
| GO Molecular Functions | GO:0023023 | MHC protein complex binding | -6.5 | 11 | 8 | B2m\|Cd4\|Cd8a\|Cd74\|Klrc1\|Klrc2\|Klrc3\|H2-Eb2 |
| GO Molecular Functions | GO:0045028 | G protein-coupled purinergic nucleotide receptor activity | -6.1 | 16 | 6 | Ptafr\|P2ry13\|P2ry10\|Gpr87\|P2ry14\|Gpr171 |
| GO Molecular Functions | GO:0005125 | cytokine activity | -6 | 3.2 | 23 | Bmp5\|Fasl\|Gdf3\|Ifnb1\|Ifng\|Il12b\|Il1a\|Il1b\|Il1rn\|Il2\|Il4\|Il6\|Ccl21a\|Ccl2\|Ccl3\|Ccl4\|Ccl5\|Cxcl2\|Spp1\|Cxcl13\|Il21\|Wnt16\|Tnfsf15 |
| GO Molecular Functions | GO:0016818 | hydrolase activity, acting on acid anhydrides, in phosphorus-containing anhydrides | -5.8 | 2.2 | 41 | Arhgdib\|Blm\|Smc2\|Gbp2\|Gng2\|Hspa5\|Hspa1b\|Mx1\|Kif20a\|Rac2\|Rhof\|Srp54a\|Trpm2\|Septin1\|Rab37\|Iigp1\|Fignl1\|Ppa1\|Trip13\|Kif18b\|Kif23\|Rasd2\|Gbp6\|Hspa1a\|Kif15\|Agap2\|Gbp5\|Rerg\|Gbp9\|Gm4951\|F830016B08Rik\|Kif20b\|Prune2\|Kif14\|Tubb1\|Gm12185\|Gbp10\|Gbp11\|Srp54b\|Rnf213\|Srcap |
| GO Molecular Functions | GO:0016817 | hydrolase activity, acting on acid anhydrides | -5.8 | 2.2 | 41 | Arhgdib\|Blm\|Smc2\|Gbp2\|Gng2\|Hspa5\|Hspa1b\|Mx1\|Kif20a\|Rac2\|Rhof\|Srp54a\|Trpm2\|Septin1\|Rab37\|Iigp1\|Fignl1\|Ppa1\|Trip13\|Kif18b\|Kif23\|Rasd2\|Gbp6\|Hspa1a\|Kif15\|Agap2\|Gbp5\|Rerg\|Gbp9\|Gm4951\|F830016B08Rik\|Kif20b\|Prune2\|Kif14\|Tubb1\|Gm12185\|Gbp10\|Gbp11\|Srp54b\|Rnf213\|Srcap |
| GO Molecular Functions | GO:0016462 | pyrophosphatase activity | -5.6 | 2.2 | 40 | Arhgdib\|Smc2\|Gbp2\|Gng2\|Hspa5\|Hspa1b\|Mx1\|Kif20a\|Rac2\|Rhof\|Srp54a\|Trpm2\|Septin1\|Rab37\|Iigp1\|Fignl1\|Ppa1\|Trip13\|Kif18b\|Kif23\|Rasd2\|Gbp6\|Hspa1a\|Kif15\|Agap2\|Gbp5\|Rerg\|Gbp9\|Gm4951\|F830016B08Rik\|Kif20b\|Prune2\|Kif14\|Tubb1\|Gm12185\|Gbp10\|Gbp11\|Srp54b\|Rnf213\|Srcap |
| GO Molecular Functions | GO:0017111 | nucleoside-triphosphatase activity | -5.4 | 2.3 | 37 | Arhgdib\|Smc2\|Gbp2\|Gng2\|Hspa5\|Hspa1b\|Mx1\|Kif20a\|Rac2\|Rhof\|Srp54a\|Septin1\|Rab37\|Iigp1\|Fignl1\|Trip13\|Kif18b\|Kif23\|Rasd2\|Gbp6\|Hspa1a\|Kif15\|Agap2\|Gbp5\|Rerg\|Gbp9\|Gm4951\|F830016B08Rik\|Kif20b\|Kif14\|Tubb1\|Gm12185\|Gbp10\|Gbp11\|Srp54b\|Rnf213\|Srcap |
| GO Molecular Functions | GO:0005126 | cytokine receptor binding | -5.1 | 2.6 | 26 | Fasl\|Ifnb1\|Ifng\|Il12b\|Il12rb1\|Il1a\|Il1b\|Il1rn\|Il2\|Il4\|Il6\|Myd88\|Ccl21a\|Ccl2\|Ccl3\|Ccl4\|Ccl5\|Cxcl2\|Traf6\|Ccrl2\|Cxcl13\|Stap1\|Il21\|Tlr9\|Cd300lf\|Tnfsf15 |
| GO Molecular Functions | GO:0016502 | nucleotide receptor activity | -4.7 | 10 | 6 | Ptafr\|P2ry13\|P2ry10\|Gpr87\|P2ry14\|Gpr171 |
| KEGG Pathway | mmu04060 | Cytokine-cytokine receptor interaction | -17 | 4.9 | 41 | Cxcr5\|Ccr6\|Cxcr2\|Cxcr3\|Ccr1\|Ccr9\|Ccr2\|Ccr8\|Csf2rb\|Csf2rb2\|Csf3r\|Fasl\|Hgf\|Ifnar2\|Ifnb1\|Ifng\|Il12b\|Il12rb1\|Il12rb2\|Il1a\|Il1b\|Il2\|Il2ra\|Il2rb\|Il2rg\|Il4\|Il6\|Ccl21a\|Ccl2\|Ccl3\|Ccl4\|Ccl5\|Cxcl2\|Tnfrsf8\|Cxcl13\|Il21r\|Il21\|Tnfrsf13c\|Tnfrsf14\|Il22ra2\|Tnfsf15 |
| KEGG Pathway | mmu05162 | Measles | -14 | 6.2 | 27 | Cd28\|Cd3e\|Cd3g\|Fasl\|Fcgr2b\|Hspa1b\|Ifnar2\|Ifnb1\|Ifng\|Il12b\|Il1a\|Il1b\|Il2\|Il2ra\|Il2rb\|Il2rg\|Il4\|Il6\|Myd88\|Tnfaip3\|Traf6\|Tlr2\|Irf7\|Tlr9\|Tlr7\|Hspa1a\|Oas3 |
| KEGG Pathway | mmu04640 | Hematopoietic cell lineage | -8.9 | 5.9 | 18 | Ms4a1\|Cd33\|Cd3e\|Cd3g\|Cd4\|Cd5\|Cd8a\|Cd8b1\|Csf3r\|Fcer2a\|Il1a\|Il1b\|Il2ra\|Il4\|Il6\|Itga4\|Dntt\|Siglech |
| KEGG Pathway | mmu05321 | Inflammatory bowel disease (IBD) | -8.5 | 7.6 | 14 | Ifng\|Il12b\|Il12rb1\|Il12rb2\|Il1a\|Il1b\|Il2\|Il2rg\|Il4\|Il6\|Foxp3\|Tlr2\|Il21r\|Il21 |
| KEGG Pathway | mmu04621 | NOD-like receptor signaling pathway | -8.4 | 4.3 | 23 | Camp\|Cybb\|Gbp2\|Ifi204\|Ifnar2\|Ifnb1\|Il1b\|Il6\|Myd88\|Naip2\|Ccl2\|Ccl5\|Cxcl2\|Tnfaip3\|Traf6\|Trpm2\|Irf7\|Mefv\|Ripk3\|Sting1\|Nlrp3\|Gbp5\|Oas3 |
| KEGG Pathway | mmu04660 | T cell receptor signaling pathway | -8.4 | 5.5 | 18 | Cd28\|Cd3e\|Cd3g\|Cd4\|Cd8a\|Cd8b1\|Ifng\|Il2\|Il4\|Itk\|Lck\|Grap2\|Nfkbie\|Nr4a3\|Ptprc\|Icos\|Card11\|Raf1 |
| KEGG Pathway | mmu04659 | Th17 cell differentiation | -7.7 | 5.3 | 17 | Cd3e\|Cd3g\|Cd4\|Ifng\|Il12rb1\|Il1b\|Il2\|Il2ra\|Il2rb\|Il2rg\|Il4\|Il6\|Lck\|Nfkbie\|Foxp3\|Il21r\|Il21 |
| KEGG Pathway | mmu05142 | Chagas disease (American trypanosomiasis) | -7.7 | 5.3 | 17 | Cd3e\|Cd3g\|Fasl\|Ifnb1\|Ifng\|Il12b\|Il1b\|Il2\|Il6\|Myd88\|Serpine1\|Ccl2\|Ccl3\|Ccl5\|Traf6\|Tlr2\|Tlr9 |
| KEGG Pathway | mmu04658 | Th1 and Th2 cell differentiation | -7.1 | 5.5 | 15 | Runx3\|Cd3e\|Cd3g\|Cd4\|Ifng\|Il12b\|Il12rb1\|Il12rb2\|Il2\|Il2ra\|Il2rb\|Il2rg\|Il4\|Lck\|Nfkbie |
| KEGG Pathway | mmu04620 | Toll-like receptor signaling pathway | -7 | 5 | 16 | Ifnar2\|Ifnb1\|Il12b\|Il1b\|Il6\|Myd88\|Ccl3\|Ccl4\|Ccl5\|Spp1\|Traf6\|Tlr2\|Irf7\|Tlr9\|Tlr7\|Tlr8 |
| KEGG Pathway | mmu05164 | Influenza A | -7 | 3.9 | 21 | Ciita\|Fasl\|Hspa1b\|Icam1\|Ifnar2\|Ifnb1\|Ifng\|Il12b\|Il1a\|Il1b\|Il6\|Kpna2\|Myd88\|Ccl2\|Ccl5\|Irf7\|Raf1\|Tlr7\|Hspa1a\|Nlrp3\|Oas3 |
| KEGG Pathway | mmu05340 | Primary immunodeficiency | -7 | 8.8 | 10 | Ciita\|Cd3e\|Cd4\|Cd8a\|Cd8b1\|Il2rg\|Lck\|Ptprc\|Icos\|Tnfrsf13c |
| KEGG Pathway | mmu04650 | Natural killer cell mediated cytotoxicity | -6.9 | 4.4 | 18 | Cd48\|Fasl\|Fcer1g\|Gzmb\|H2-K1\|Icam1\|Ifnar2\|Ifnb1\|Ifng\|Itgal\|Itgb2\|Klrc1\|Klrc2\|Lck\|Prf1\|Rac2\|Klrk1\|Raf1 |
| KEGG Pathway | mmu04062 | Chemokine signaling pathway | -6.9 | 3.7 | 22 | Adcy9\|Cxcr5\|Ccr6\|Cxcr2\|Cxcr3\|Ccr1\|Ccr9\|Ccr2\|Ccr8\|Gng2\|Itk\|Ccl21a\|Rac2\|Ccl2\|Ccl3\|Ccl4\|Ccl5\|Cxcl2\|Was\|Cxcl13\|Dock2\|Raf1 |
| KEGG Pathway | mmu04630 | Jak-STAT signaling pathway | -6.8 | 4 | 20 | Csf2rb\|Csf2rb2\|Csf3r\|Ifnar2\|Ifnb1\|Ifng\|Il12b\|Il12rb1\|Il12rb2\|Il13ra2\|Il2\|Il2ra\|Il2rb\|Il2rg\|Il4\|Il6\|Il21r\|Il21\|Raf1\|Il22ra2 |
| KEGG Pathway | mmu04612 | Antigen processing and presentation | -6.8 | 5.2 | 15 | B2m\|Ciita\|Cd4\|Cd8a\|Cd8b1\|Hspa5\|H2-K1\|H2-T24\|Hspa1b\|Ifng\|Cd74\|Klrc1\|Klrc2\|Klrc3\|Hspa1a |
| KEGG Pathway | mmu05144 | Malaria | -6.5 | 7 | 11 | Hgf\|Icam1\|Ifng\|Il1b\|Il6\|Itgal\|Itgb2\|Myd88\|Ccl2\|Tlr2\|Tlr9 |
| KEGG Pathway | mmu05332 | Graft-versus-host disease | -6 | 5.7 | 12 | Cd28\|Fasl\|Gzmb\|H2-K1\|H2-T24\|Ifng\|Il1a\|Il1b\|Il2\|Il6\|Klrc1\|Prf1 |
| KEGG Pathway | mmu05152 | Tuberculosis | -5.9 | 3.5 | 20 | Ciita\|Camp\|Fcer1g\|Fcgr2b\|Fcgr3\|Ifnb1\|Ifng\|Cd74\|Il12b\|Il1a\|Il1b\|Il6\|Itgb2\|Myd88\|Traf6\|Tlr2\|Clec4e\|Clec7a\|Tlr9\|Raf1 |
| KEGG Pathway | mmu04514 | Cell adhesion molecules (CAMs) | -5.7 | 3.5 | 19 | Cd28\|Cd4\|Cd6\|Cd8a\|Cd8b1\|H2-K1\|H2-T24\|Icam1\|Itga4\|Itgal\|Itgb2\|Ptprc\|Selplg\|Siglec1\|Icos\|Cd274\|Cd226\|Nrcam\|Tigit |
