# Supplementary material for: Exploring the immune-inflammatory mechanism of Maxing Shigan Decoction in treating influenza virus A-induced pneumonia based on an integrated strategy of single-cell transcriptomics and systems biology
Source: Eur J Med Res. 2024 Apr 15;29:234. doi: 10.1186/s40001-024-01777-9 (PMC11017673; doi:10.1186/s40001-024-01777-9)
Supplement: Supplementary file 4 — Additional file 4: Table S3. Enrichment analysis of all gene. [file 40001_2024_1777_MOESM4_ESM.docx]

Table S3 Enrichment analysis of All Gene

| **Category** | **GO** | **Description** | **LogP** | **Enrichment** | **Counts** | **Genes** |
| --- | --- | --- | --- | --- | --- | --- |
| GO Biological Processes | GO:0002694 | regulation of leukocyte activation | -26 | 2.9 | 119 | Atm\|B2m\|Blm\|Zfp36l2\|Runx3\|Ccr6\|Cd28\|Cd33\|Cd3e\|Cd4\|Cd5\|Cd6\|Cd84\|Ccr2\|Cst7\|Ctsc\|Egr3\|Fanca\|Fcer1g\|Fcgr2b\|Fgl2\|Lilrb4a\|Foxj1\|Ifnb1\|Ifng\|Ighg2b\|Ighg1\|Ihh\|Cd74\|Il12b\|Il12rb1\|Il13ra2\|Il1a\|Il1b\|Il2\|Il2ra\|Il2rg\|Il4\|Il6\|Itgal\|Itgb2\|Klrc1\|Klrc2\|Lck\|Mmp8\|Myd88\|Nr4a3\|Ccl21a\|Ptafr\|Ptpn22\|Ptprc\|Rac2\|Rorc\|Ccl2\|Ccl5\|Foxp3\|Sfrp1\|Sox4\|Tff2\|Thy1\|Tnfaip3\|Traf6\|Scgb1a1\|Ikzf3\|Zp3\|Klrk1\|Icos\|Crtam\|Ripk3\|Clec7a\|Stap1\|Pglyrp2\|Klrc3\|Il21\|Cd274\|Tespa1\|Tnfrsf13c\|Slamf7\|Tlr9\|Nckap1l\|Card11\|Havcr2\|Ighv9-1\|Ighv1-78\|Cgas\|Nlrp3\|Nr1d1\|Cd226\|Tnfrsf14\|Zc3h12d\|Ighv14-3\|Cd300lf\|Rasal3\|Ighg3\|Ighv10-3\|Ighv1-64\|Ighv1-66\|H2-Eb2\|Ighv1-72\|Ighv9-2\|Ighv14-4\|Ighv1-12\|Ighv1-26\|Ighv5-16\|Ighv9-4\|Ighv14-2\|Ighv3-3\|Ighv1-7\|Ighv1-85\|Ighv2-2\|Ighv5-17\|Ighv9-3\|Ighv1-39\|Ighv1-53\|Ighv8-8\|Ighv1-58\|Ighv1-63\|Ighv8-12\|Tigit |
| GO Biological Processes | GO:0050865 | regulation of cell activation | -25 | 2.8 | 122 | Apoe\|Atm\|B2m\|Blm\|Zfp36l2\|Capn3\|Runx3\|Ccr6\|Cd28\|Cd33\|Cd3e\|Cd4\|Cd5\|Cd6\|Cd84\|Ccr2\|Cst7\|Ctsc\|Egr3\|Fanca\|Fcer1g\|Fcgr2b\|Fgl2\|Lilrb4a\|Foxj1\|Ifnb1\|Ifng\|Ighg2b\|Ighg1\|Ihh\|Cd74\|Il12b\|Il12rb1\|Il13ra2\|Il1a\|Il1b\|Il2\|Il2ra\|Il2rg\|Il4\|Il6\|Itgal\|Itgb2\|Klrc1\|Klrc2\|Lck\|Mmp8\|Myd88\|Nr4a3\|Ccl21a\|Ptafr\|Ptpn22\|Ptprc\|Rac2\|Rorc\|Ccl2\|Ccl5\|Foxp3\|Sfrp1\|Sox4\|Tff2\|Thy1\|Tnfaip3\|Traf6\|Scgb1a1\|Ikzf3\|Zp3\|Klrk1\|Icos\|Crtam\|Plek\|Ripk3\|Clec7a\|Stap1\|Pglyrp2\|Klrc3\|Il21\|Cd274\|Tespa1\|Tnfrsf13c\|Slamf7\|Tlr9\|Nckap1l\|Card11\|Havcr2\|Ighv9-1\|Ighv1-78\|Cgas\|Nlrp3\|Nr1d1\|Cd226\|Tnfrsf14\|Zc3h12d\|Ighv14-3\|Cd300lf\|Rasal3\|Ighg3\|Ighv10-3\|Ighv1-64\|Ighv1-66\|H2-Eb2\|Ighv1-72\|Ighv9-2\|Ighv14-4\|Ighv1-12\|Ighv1-26\|Ighv5-16\|Ighv9-4\|Ighv14-2\|Ighv3-3\|Ighv1-7\|Ighv1-85\|Ighv2-2\|Ighv5-17\|Ighv9-3\|Ighv1-39\|Ighv1-53\|Ighv8-8\|Ighv1-58\|Ighv1-63\|Ighv8-12\|Tigit |
| GO Biological Processes | GO:0051249 | regulation of lymphocyte activation | -24 | 3 | 103 | Atm\|B2m\|Blm\|Zfp36l2\|Runx3\|Ccr6\|Cd28\|Cd3e\|Cd4\|Cd5\|Cd6\|Ccr2\|Egr3\|Fanca\|Fcgr2b\|Fgl2\|Lilrb4a\|Foxj1\|Ifnb1\|Ifng\|Ighg2b\|Ighg1\|Ihh\|Cd74\|Il12b\|Il12rb1\|Il1a\|Il1b\|Il2\|Il2ra\|Il2rg\|Il4\|Il6\|Itgal\|Klrc1\|Klrc2\|Lck\|Myd88\|Ccl21a\|Ptpn22\|Ptprc\|Rac2\|Rorc\|Ccl2\|Ccl5\|Foxp3\|Sfrp1\|Sox4\|Thy1\|Tnfaip3\|Traf6\|Scgb1a1\|Ikzf3\|Zp3\|Icos\|Crtam\|Ripk3\|Clec7a\|Pglyrp2\|Klrc3\|Il21\|Cd274\|Tespa1\|Tnfrsf13c\|Slamf7\|Tlr9\|Nckap1l\|Card11\|Havcr2\|Ighv9-1\|Ighv1-78\|Cgas\|Nlrp3\|Tnfrsf14\|Zc3h12d\|Ighv14-3\|Rasal3\|Ighg3\|Ighv10-3\|Ighv1-64\|Ighv1-66\|H2-Eb2\|Ighv1-72\|Ighv9-2\|Ighv14-4\|Ighv1-12\|Ighv1-26\|Ighv5-16\|Ighv9-4\|Ighv14-2\|Ighv3-3\|Ighv1-7\|Ighv1-85\|Ighv2-2\|Ighv5-17\|Ighv9-3\|Ighv1-39\|Ighv1-53\|Ighv8-8\|Ighv1-58\|Ighv1-63\|Ighv8-12\|Tigit |
| GO Biological Processes | GO:0050867 | positive regulation of cell activation | -23 | 3.2 | 93 | B2m\|Blm\|Capn3\|Runx3\|Cd28\|Cd3e\|Cd4\|Cd5\|Cd6\|Ccr2\|Ctsc\|Egr3\|Fcer1g\|Lilrb4a\|Ifng\|Ighg2b\|Ighg1\|Ihh\|Cd74\|Il12b\|Il12rb1\|Il1a\|Il1b\|Il2\|Il2ra\|Il2rg\|Il4\|Il6\|Itgal\|Itgb2\|Klrc1\|Klrc2\|Lck\|Mmp8\|Myd88\|Nr4a3\|Ccl21a\|Ptafr\|Ptpn22\|Ptprc\|Ccl2\|Ccl5\|Foxp3\|Sox4\|Thy1\|Traf6\|Zp3\|Klrk1\|Icos\|Plek\|Clec7a\|Stap1\|Klrc3\|Il21\|Cd274\|Tespa1\|Tnfrsf13c\|Tlr9\|Nckap1l\|Card11\|Havcr2\|Ighv9-1\|Ighv1-78\|Nlrp3\|Cd226\|Tnfrsf14\|Ighv14-3\|Rasal3\|Ighg3\|Ighv10-3\|Ighv1-64\|Ighv1-66\|H2-Eb2\|Ighv1-72\|Ighv9-2\|Ighv14-4\|Ighv1-12\|Ighv1-26\|Ighv5-16\|Ighv9-4\|Ighv14-2\|Ighv3-3\|Ighv1-7\|Ighv1-85\|Ighv2-2\|Ighv5-17\|Ighv9-3\|Ighv1-39\|Ighv1-53\|Ighv8-8\|Ighv1-58\|Ighv1-63\|Ighv8-12 |
| GO Biological Processes | GO:0002696 | positive regulation of leukocyte activation | -23 | 3.3 | 91 | B2m\|Blm\|Runx3\|Cd28\|Cd3e\|Cd4\|Cd5\|Cd6\|Ccr2\|Ctsc\|Egr3\|Fcer1g\|Lilrb4a\|Ifng\|Ighg2b\|Ighg1\|Ihh\|Cd74\|Il12b\|Il12rb1\|Il1a\|Il1b\|Il2\|Il2ra\|Il2rg\|Il4\|Il6\|Itgal\|Itgb2\|Klrc1\|Klrc2\|Lck\|Mmp8\|Myd88\|Nr4a3\|Ccl21a\|Ptafr\|Ptpn22\|Ptprc\|Ccl2\|Ccl5\|Foxp3\|Sox4\|Thy1\|Traf6\|Zp3\|Klrk1\|Icos\|Clec7a\|Stap1\|Klrc3\|Il21\|Cd274\|Tespa1\|Tnfrsf13c\|Tlr9\|Nckap1l\|Card11\|Havcr2\|Ighv9-1\|Ighv1-78\|Nlrp3\|Cd226\|Tnfrsf14\|Ighv14-3\|Rasal3\|Ighg3\|Ighv10-3\|Ighv1-64\|Ighv1-66\|H2-Eb2\|Ighv1-72\|Ighv9-2\|Ighv14-4\|Ighv1-12\|Ighv1-26\|Ighv5-16\|Ighv9-4\|Ighv14-2\|Ighv3-3\|Ighv1-7\|Ighv1-85\|Ighv2-2\|Ighv5-17\|Ighv9-3\|Ighv1-39\|Ighv1-53\|Ighv8-8\|Ighv1-58\|Ighv1-63\|Ighv8-12 |
| GO Biological Processes | GO:0050778 | positive regulation of immune response | -23 | 2.8 | 111 | Cfd\|B2m\|Bmx\|C3ar1\|C5ar1\|Ms4a1\|Cd28\|Cd3e\|Cd4\|Ccr2\|Coch\|Fcer1g\|Fcer2a\|Fcgr3\|Fcnb\|Lilrb4a\|H2-Bl\|H2-K1\|H2-Q1\|H2-T24\|Ifi203\|Ifi204\|Ifng\|Ighg2b\|Ighg1\|Cd74\|Il12b\|Il12rb1\|Il1b\|Il2\|Il4\|Il6\|Itgb2\|Itk\|Klrc1\|Klrc2\|Krt1\|Lck\|Nr4a3\|Slc11a1\|Pde4b\|Ptafr\|Ptpn22\|Ptprc\|Foxp3\|Thy1\|Traf6\|Usp9x\|Zp3\|Fyb\|Sh2b2\|Tlr2\|Klrk1\|Elane\|Irf7\|Crtam\|Stap1\|Klrc3\|Zbp1\|Il21\|Cd274\|Tespa1\|Tasl\|Tnfrsf13c\|Sting1\|Skap1\|Tlr9\|Nckap1l\|Lpxn\|Card11\|Tlr8\|Havcr2\|Ighv9-1\|Trim30d\|Themis\|Ighv1-78\|Cgas\|Nlrp3\|Cd226\|Gbp5\|A2m\|Ifi209\|Ighv14-3\|C5ar2\|Ubash3a\|Fcrl5\|Ighg3\|Ighv10-3\|Ighv1-64\|Ighv1-66\|Ifi214\|Ighv1-72\|Ighv9-2\|Ighv14-4\|Ighv1-12\|Ighv1-26\|Ighv5-16\|Ighv9-4\|Ighv14-2\|Ighv3-3\|Ighv1-7\|Ighv1-85\|Ighv2-2\|Ighv5-17\|Ighv9-3\|Ighv1-39\|Ighv1-53\|Ighv8-8\|Ighv1-58\|Ighv1-63\|Ighv8-12 |
| GO Biological Processes | GO:0002250 | adaptive immune response | -23 | 3 | 103 | B2m\|Bmx\|C3ar1\|Camk4\|Ccr6\|Cd28\|Cd3e\|Cd3g\|Cd4\|Cd48\|Cd84\|Cd8a\|Cd8b1\|Ccr2\|Ctsc\|Ctsh\|Eomes\|Fcer1g\|Fcer2a\|Fcgr2b\|Fcgr3\|Fgl2\|Fut7\|Lilrb4a\|Gzmb\|H2-Bl\|H2-K1\|H2-Q1\|H2-T24\|Foxj1\|Icam1\|Ifnb1\|Ifng\|Ighg2b\|Ighg1\|Cd74\|Il12b\|Il12rb1\|Il13ra2\|Il1b\|Il2\|Il4\|Il6\|Itk\|Ly9\|Myd88\|Slc11a1\|Prf1\|Ptprc\|Rorc\|Foxp3\|Serpinb9b\|Serpina3g\|Tnfaip3\|Traf6\|Was\|Zp3\|Klrk1\|Irf7\|Crtam\|Ripk3\|Rnf8\|Cd274\|Rnf125\|Mcoln2\|Tnfrsf13c\|Slamf7\|Skap1\|Nckap1l\|Iglv2\|Havcr2\|Ighv9-1\|Themis\|Ighv1-78\|Nlrp3\|Cd226\|Tnfrsf14\|Ighv14-3\|Ighg3\|Ighv10-3\|Ighv1-64\|Ighv1-66\|H2-Eb2\|Ighv1-72\|Ighv9-2\|Ighv14-4\|Ighv1-12\|Ighv1-26\|Ighv5-16\|Ighv9-4\|Ighv14-2\|Ighv3-3\|Ighv1-7\|Ighv1-85\|Ighv2-2\|Ighv5-17\|Ighv9-3\|Ighv1-39\|Ighv1-53\|Ighv8-8\|Ighv1-58\|Ighv1-63\|Ighv8-12 |
| GO Biological Processes | GO:0002443 | leukocyte mediated immunity | -22 | 3 | 99 | B2m\|Ccr6\|Cd28\|Cd84\|Cd8a\|Ccr2\|Ctsc\|Ctsh\|Dnase1l3\|Fcer1g\|Fcer2a\|Fcgr2b\|Fcgr3\|Fgl2\|Fut7\|Lilrb4a\|Gzmb\|H2-Bl\|H2-K1\|H2-Q1\|H2-T24\|Foxj1\|Icam1\|Ifnb1\|Ifng\|Ighg2b\|Ighg1\|Cd74\|Il12b\|Il13ra2\|Il1b\|Il2\|Il4\|Il6\|Itgb2\|Klrc1\|Klrc2\|Myd88\|Myo1f\|Nr4a3\|Slc11a1\|Prf1\|Ptafr\|Ptprc\|Rac2\|Ccl2\|Foxp3\|Serpinb9b\|Traf6\|Was\|Zp3\|Tlr2\|Klrk1\|Elane\|Irf7\|Crtam\|Ripk3\|Stap1\|Klrc3\|Rnf8\|Il21\|Klrb1b\|Tlr9\|Cd96\|Spon2\|Nckap1l\|Havcr2\|Ighv9-1\|Ighv1-78\|Nlrp3\|Cd226\|Ighv14-3\|Scimp\|Ighg3\|Ighv10-3\|Ighv1-64\|Ighv1-66\|H2-Eb2\|Ighv1-72\|Ighv9-2\|Ighv14-4\|Ighv1-12\|Ighv1-26\|Ighv5-16\|Ighv9-4\|Ighv14-2\|Ighv3-3\|Ighv1-7\|Ighv1-85\|H60c\|Ighv2-2\|Ighv5-17\|Ighv9-3\|Ighv1-39\|Ighv1-53\|Ighv8-8\|Ighv1-58\|Ighv1-63\|Ighv8-12 |
| GO Biological Processes | GO:0051251 | positive regulation of lymphocyte activation | -22 | 3.4 | 82 | B2m\|Blm\|Runx3\|Cd28\|Cd3e\|Cd4\|Cd5\|Cd6\|Ccr2\|Egr3\|Lilrb4a\|Ifng\|Ighg2b\|Ighg1\|Ihh\|Cd74\|Il12b\|Il12rb1\|Il1a\|Il1b\|Il2\|Il2ra\|Il2rg\|Il4\|Il6\|Itgal\|Klrc1\|Klrc2\|Lck\|Myd88\|Ccl21a\|Ptpn22\|Ptprc\|Ccl2\|Ccl5\|Foxp3\|Sox4\|Thy1\|Traf6\|Zp3\|Icos\|Clec7a\|Klrc3\|Il21\|Cd274\|Tespa1\|Tnfrsf13c\|Tlr9\|Nckap1l\|Card11\|Havcr2\|Ighv9-1\|Ighv1-78\|Nlrp3\|Tnfrsf14\|Ighv14-3\|Rasal3\|Ighg3\|Ighv10-3\|Ighv1-64\|Ighv1-66\|H2-Eb2\|Ighv1-72\|Ighv9-2\|Ighv14-4\|Ighv1-12\|Ighv1-26\|Ighv5-16\|Ighv9-4\|Ighv14-2\|Ighv3-3\|Ighv1-7\|Ighv1-85\|Ighv2-2\|Ighv5-17\|Ighv9-3\|Ighv1-39\|Ighv1-53\|Ighv8-8\|Ighv1-58\|Ighv1-63\|Ighv8-12 |
| GO Biological Processes | GO:0002449 | lymphocyte mediated immunity | -21 | 3.2 | 85 | B2m\|Ccr6\|Cd28\|Cd8a\|Ccr2\|Ctsc\|Ctsh\|Fcer1g\|Fcer2a\|Fcgr2b\|Fcgr3\|Fgl2\|Fut7\|Lilrb4a\|Gzmb\|H2-Bl\|H2-K1\|H2-Q1\|H2-T24\|Foxj1\|Icam1\|Ifnb1\|Ifng\|Ighg2b\|Ighg1\|Cd74\|Il12b\|Il13ra2\|Il1b\|Il2\|Il4\|Il6\|Klrc1\|Klrc2\|Myd88\|Slc11a1\|Prf1\|Ptprc\|Foxp3\|Serpinb9b\|Traf6\|Was\|Zp3\|Klrk1\|Irf7\|Crtam\|Ripk3\|Klrc3\|Rnf8\|Il21\|Klrb1b\|Cd96\|Nckap1l\|Havcr2\|Ighv9-1\|Ighv1-78\|Nlrp3\|Cd226\|Ighv14-3\|Ighg3\|Ighv10-3\|Ighv1-64\|Ighv1-66\|H2-Eb2\|Ighv1-72\|Ighv9-2\|Ighv14-4\|Ighv1-12\|Ighv1-26\|Ighv5-16\|Ighv9-4\|Ighv14-2\|Ighv3-3\|Ighv1-7\|Ighv1-85\|H60c\|Ighv2-2\|Ighv5-17\|Ighv9-3\|Ighv1-39\|Ighv1-53\|Ighv8-8\|Ighv1-58\|Ighv1-63\|Ighv8-12 |
| GO Biological Processes | GO:0002253 | activation of immune response | -20 | 3.3 | 80 | Cfd\|Bmx\|C3ar1\|C5ar1\|Ms4a1\|Cd28\|Cd3e\|Fcer1g\|Fcnb\|Lilrb4a\|Ifi203\|Ifi204\|Ifng\|Ighg2b\|Ighg1\|Il1b\|Itk\|Klrc1\|Klrc2\|Krt1\|Lck\|Nr4a3\|Pde4b\|Ptpn22\|Ptprc\|Foxp3\|Thy1\|Traf6\|Usp9x\|Fyb\|Sh2b2\|Tlr2\|Klrk1\|Stap1\|Klrc3\|Zbp1\|Tespa1\|Sting1\|Skap1\|Tlr9\|Nckap1l\|Lpxn\|Card11\|Ighv9-1\|Trim30d\|Themis\|Ighv1-78\|Cgas\|Cd226\|A2m\|Ifi209\|Ighv14-3\|C5ar2\|Ubash3a\|Fcrl5\|Ighg3\|Ighv10-3\|Ighv1-64\|Ighv1-66\|Ifi214\|Ighv1-72\|Ighv9-2\|Ighv14-4\|Ighv1-12\|Ighv1-26\|Ighv5-16\|Ighv9-4\|Ighv14-2\|Ighv3-3\|Ighv1-7\|Ighv1-85\|Ighv2-2\|Ighv5-17\|Ighv9-3\|Ighv1-39\|Ighv1-53\|Ighv8-8\|Ighv1-58\|Ighv1-63\|Ighv8-12 |
| GO Biological Processes | GO:0002764 | immune response-regulating signaling pathway | -20 | 3.1 | 87 | Bmx\|C3ar1\|C5ar1\|Ms4a1\|Cd28\|Cd33\|Cd3e\|Ctsh\|Fcer1g\|Lilrb4a\|Ifng\|Ighg2b\|Ighg1\|Itk\|Klrc1\|Klrc2\|Lck\|Cd180\|Myd88\|Nr4a3\|Pde4b\|Ptpn22\|Ptprc\|Foxp3\|Thy1\|Tnfaip3\|Traf6\|Usp9x\|Fyb\|Sh2b2\|Tlr2\|Klrk1\|Irf7\|Clec4e\|Stap1\|Klrc3\|Slc15a3\|Tespa1\|Rnf125\|Tasl\|Skap1\|Tlr9\|Pik3ap1\|Nckap1l\|Lpxn\|Card11\|Tlr7\|Tlr8\|Havcr2\|Ighv9-1\|Themis\|Lacc1\|Ighv1-78\|Nr1d1\|Cd226\|Ighv14-3\|Oas3\|Cd300lf\|C5ar2\|Scimp\|Ubash3a\|Fcrl5\|Ighg3\|Ighv10-3\|Ighv1-64\|Ighv1-66\|Tlr12\|Ighv1-72\|Ighv9-2\|Ighv14-4\|Ighv1-12\|Ighv1-26\|Ighv5-16\|Ighv9-4\|Ighv14-2\|Ighv3-3\|Ighv1-7\|Ighv1-85\|Ighv2-2\|Ighv5-17\|Ighv9-3\|Ighv1-39\|Ighv1-53\|Ighv8-8\|Ighv1-58\|Ighv1-63\|Ighv8-12 |
| GO Biological Processes | GO:0002460 | adaptive immune response based on somatic recombination of immune receptors built from immunoglobulin superfamily domains | -20 | 3.1 | 84 | B2m\|C3ar1\|Ccr6\|Cd28\|Cd4\|Cd8a\|Ccr2\|Ctsc\|Ctsh\|Fcer1g\|Fcer2a\|Fcgr2b\|Fcgr3\|Fgl2\|Fut7\|Lilrb4a\|Gzmb\|H2-Bl\|H2-K1\|H2-Q1\|H2-T24\|Foxj1\|Icam1\|Ifnb1\|Ifng\|Ighg2b\|Ighg1\|Cd74\|Il12b\|Il12rb1\|Il13ra2\|Il1b\|Il2\|Il4\|Il6\|Ly9\|Myd88\|Slc11a1\|Prf1\|Ptprc\|Rorc\|Foxp3\|Serpinb9b\|Tnfaip3\|Traf6\|Was\|Zp3\|Irf7\|Ripk3\|Rnf8\|Cd274\|Tnfrsf13c\|Nckap1l\|Havcr2\|Ighv9-1\|Ighv1-78\|Nlrp3\|Cd226\|Ighv14-3\|Ighg3\|Ighv10-3\|Ighv1-64\|Ighv1-66\|H2-Eb2\|Ighv1-72\|Ighv9-2\|Ighv14-4\|Ighv1-12\|Ighv1-26\|Ighv5-16\|Ighv9-4\|Ighv14-2\|Ighv3-3\|Ighv1-7\|Ighv1-85\|Ighv2-2\|Ighv5-17\|Ighv9-3\|Ighv1-39\|Ighv1-53\|Ighv8-8\|Ighv1-58\|Ighv1-63\|Ighv8-12 |
| GO Biological Processes | GO:1903039 | positive regulation of leukocyte cell-cell adhesion | -20 | 4.2 | 56 | B2m\|Blm\|Runx3\|Cd28\|Cd3e\|Cd4\|Cd5\|Cd6\|Ccr2\|Egr3\|Fut7\|Lilrb4a\|Has2\|Icam1\|Ifng\|Ihh\|Cd74\|Il12b\|Il12rb1\|Il1a\|Il1b\|Il2\|Il2ra\|Il2rg\|Il4\|Il6\|Itga4\|Itgal\|Itgb2\|Lck\|Nr4a3\|Ccl21a\|Ptafr\|Ptpn22\|Ptprc\|Ccl2\|Ccl5\|Foxp3\|Sox4\|Thy1\|Traf6\|Zp3\|Elane\|Icos\|Il21\|Cd274\|Tespa1\|Tnfrsf13c\|Skap1\|Nckap1l\|Card11\|Havcr2\|Nlrp3\|Tnfrsf14\|Rasal3\|H2-Eb2 |
| GO Biological Processes | GO:0042110 | T cell activation | -19 | 2.9 | 89 | Chrna7\|Atp7a\|B2m\|Blm\|Zfp36l2\|Runx2\|Runx3\|Ccr6\|Cd28\|Cd3e\|Cd3g\|Cd4\|Cd48\|Cd5\|Cd6\|Cd84\|Cd8a\|Ccr9\|Ccr2\|Egr3\|Eomes\|Fanca\|Fcer1g\|Fgl2\|Fut7\|Lilrb4a\|Foxj1\|Icam1\|Ifnb1\|Ifng\|Ihh\|Cd74\|Il12b\|Il12rb1\|Il1a\|Il1b\|Il2\|Il2ra\|Il2rg\|Il4\|Il6\|Itgal\|Itgb2\|Itk\|Lck\|Ly9\|Myh9\|Slc11a1\|Lcp1\|Ccl21a\|Ptpn22\|Ptprc\|Rac2\|Rorc\|Ccl2\|Ccl5\|Foxp3\|Sox4\|Thy1\|Traf6\|Scgb1a1\|Was\|Zp3\|Icos\|Crtam\|Elf4\|Ripk3\|Clec4e\|Il21\|Cd274\|Tespa1\|Tnfrsf13c\|Slamf7\|Dock2\|Nckap1l\|Card11\|Havcr2\|Themis\|Cgas\|Nlrp3\|Tnfrsf14\|Zc3h12d\|Nlrc3\|Jaml\|Rasal3\|H2-Eb2\|Itgad\|Ccl21b\|Tigit |
| GO Biological Processes | GO:0007159 | leukocyte cell-cell adhesion | -19 | 3.4 | 70 | B2m\|Blm\|Runx3\|Cd28\|Cd3e\|Cd4\|Cd5\|Cd6\|Ccr2\|Egr3\|Fgl2\|Fut7\|Lilrb4a\|Has2\|Foxj1\|Icam1\|Ifnb1\|Ifng\|Ihh\|Cd74\|Il12b\|Il12rb1\|Il1a\|Il1b\|Il2\|Il2ra\|Il2rg\|Il4\|Il6\|Itga4\|Itgal\|Itgb2\|Lck\|Nr4a3\|Ccl21a\|Ptafr\|Ptpn22\|Ptprc\|Rac2\|S100a8\|S100a9\|Ccl2\|Ccl5\|Selplg\|Sema4d\|Foxp3\|Sox4\|Thy1\|Traf6\|Scgb1a1\|Zp3\|Elane\|Icos\|Crtam\|Il21\|Cd274\|Tespa1\|Tnfrsf13c\|Skap1\|Nckap1l\|Fermt3\|Card11\|Havcr2\|Nlrp3\|Tnfrsf14\|Zc3h12d\|Rasal3\|H2-Eb2\|Ccl21b\|Tigit |
| GO Biological Processes | GO:0002429 | immune response-activating cell surface receptor signaling pathway | -18 | 3.5 | 66 | Bmx\|C3ar1\|C5ar1\|Ms4a1\|Cd28\|Cd3e\|Fcer1g\|Lilrb4a\|Ifng\|Ighg2b\|Ighg1\|Itk\|Klrc1\|Klrc2\|Lck\|Nr4a3\|Pde4b\|Ptpn22\|Ptprc\|Foxp3\|Thy1\|Traf6\|Usp9x\|Fyb\|Sh2b2\|Tlr2\|Klrk1\|Stap1\|Klrc3\|Tespa1\|Skap1\|Nckap1l\|Lpxn\|Card11\|Ighv9-1\|Themis\|Ighv1-78\|Cd226\|Ighv14-3\|C5ar2\|Ubash3a\|Fcrl5\|Ighg3\|Ighv10-3\|Ighv1-64\|Ighv1-66\|Ighv1-72\|Ighv9-2\|Ighv14-4\|Ighv1-12\|Ighv1-26\|Ighv5-16\|Ighv9-4\|Ighv14-2\|Ighv3-3\|Ighv1-7\|Ighv1-85\|Ighv2-2\|Ighv5-17\|Ighv9-3\|Ighv1-39\|Ighv1-53\|Ighv8-8\|Ighv1-58\|Ighv1-63\|Ighv8-12 |
| GO Biological Processes | GO:0002757 | immune response-activating signal transduction | -18 | 3.5 | 66 | Bmx\|C3ar1\|C5ar1\|Ms4a1\|Cd28\|Cd3e\|Fcer1g\|Lilrb4a\|Ifng\|Ighg2b\|Ighg1\|Itk\|Klrc1\|Klrc2\|Lck\|Nr4a3\|Pde4b\|Ptpn22\|Ptprc\|Foxp3\|Thy1\|Traf6\|Usp9x\|Fyb\|Sh2b2\|Tlr2\|Klrk1\|Stap1\|Klrc3\|Tespa1\|Skap1\|Nckap1l\|Lpxn\|Card11\|Ighv9-1\|Themis\|Ighv1-78\|Cd226\|Ighv14-3\|C5ar2\|Ubash3a\|Fcrl5\|Ighg3\|Ighv10-3\|Ighv1-64\|Ighv1-66\|Ighv1-72\|Ighv9-2\|Ighv14-4\|Ighv1-12\|Ighv1-26\|Ighv5-16\|Ighv9-4\|Ighv14-2\|Ighv3-3\|Ighv1-7\|Ighv1-85\|Ighv2-2\|Ighv5-17\|Ighv9-3\|Ighv1-39\|Ighv1-53\|Ighv8-8\|Ighv1-58\|Ighv1-63\|Ighv8-12 |
| GO Biological Processes | GO:0002768 | immune response-regulating cell surface receptor signaling pathway | -18 | 3.4 | 67 | Bmx\|C3ar1\|C5ar1\|Ms4a1\|Cd28\|Cd3e\|Fcer1g\|Lilrb4a\|Ifng\|Ighg2b\|Ighg1\|Itk\|Klrc1\|Klrc2\|Lck\|Nr4a3\|Pde4b\|Ptpn22\|Ptprc\|Foxp3\|Thy1\|Traf6\|Usp9x\|Fyb\|Sh2b2\|Tlr2\|Klrk1\|Clec4e\|Stap1\|Klrc3\|Tespa1\|Skap1\|Nckap1l\|Lpxn\|Card11\|Ighv9-1\|Themis\|Ighv1-78\|Cd226\|Ighv14-3\|C5ar2\|Ubash3a\|Fcrl5\|Ighg3\|Ighv10-3\|Ighv1-64\|Ighv1-66\|Ighv1-72\|Ighv9-2\|Ighv14-4\|Ighv1-12\|Ighv1-26\|Ighv5-16\|Ighv9-4\|Ighv14-2\|Ighv3-3\|Ighv1-7\|Ighv1-85\|Ighv2-2\|Ighv5-17\|Ighv9-3\|Ighv1-39\|Ighv1-53\|Ighv8-8\|Ighv1-58\|Ighv1-63\|Ighv8-12 |
| GO Biological Processes | GO:0006954 | inflammatory response | -18 | 2.5 | 105 | Chrna7\|Adipoq\|Agtr2\|Ahcyl\|Apoa1\|Apoe\|Atm\|Ciita\|C3ar1\|C5ar1\|Camk4\|Ccr6\|Cd28\|Cd6\|Cd68\|Cxcr2\|Cxcr3\|Ccr1\|Ccr2\|Camp\|Cst7\|Ctsc\|Cybb\|Dnase1l3\|Ephx2\|F2r\|Fanca\|Fcer1g\|Fcgr2b\|Fcgr3\|Fut7\|Lilrb4a\|Hgf\|Ndst1\|Icam1\|Ifng\|Ighg2b\|Ighg1\|Il12b\|Il1a\|Il1b\|Il1rn\|Il2\|Il2ra\|Il4\|Il6\|Itgb2\|Klkb1\|Krt1\|Cd180\|Mmp8\|Myd88\|Naip2\|Slc11a1\|Serpine1\|Ccl21a\|Ptafr\|S100a8\|S100a9\|Ccl2\|Ccl3\|Ccl4\|Ccl5\|Cxcl2\|Foxp3\|Serpina3n\|Tff2\|Tnfaip3\|Trp73\|Scgb1a1\|Zp3\|Tlr2\|Pla2g7\|Pik3cg\|Elane\|Stk39\|Ccrl2\|Mefv\|Cxcl13\|Nupr1\|Clec7a\|Stap1\|Pglyrp2\|Zbp1\|Cysltr1\|Sting1\|C2cd4b\|Tlr9\|Pik3ap1\|Cd96\|Tlr7\|Tlr8\|Havcr2\|Ulk4\|Lacc1\|Nlrp3\|Nr1d1\|Afap1l2\|Gbp5\|Il22ra2\|Adamts12\|Nlrc3\|C5ar2\|Tlr12\|Ccl21b |
| GO Cellular Components | GO:0009897 | external side of plasma membrane | -27 | 3.2 | 111 | Chrna7\|Adam19\|Apoe\|B2m\|Cxcr5\|Ccr6\|Ms4a1\|Cd28\|Cd33\|Cd3e\|Cd3g\|Cd4\|Cd48\|Cd5\|Cd6\|Cd84\|Cd8a\|Cd8b1\|Cxcr2\|Cxcr3\|Ccr1\|Ccr9\|Ccr2\|Ccr8\|Csf3r\|Fasl\|Fcer1g\|Fcer2a\|Fcgr2b\|Fcgr3\|Fcnb\|Lilrb4a\|H2-Bl\|H2-K1\|H2-Q1\|H2-T24\|Icam1\|Ifng\|Ighg2b\|Ighg1\|Cd74\|Il12b\|Il12rb1\|Il12rb2\|Il13ra2\|Il2ra\|Il2rb\|Il2rg\|Il3ra\|Il4\|Il6\|Itga4\|Itgal\|Itgb2\|Klrc1\|Klrc2\|Lhcgr\|Ly9\|Ptprc\|Rorc\|Spa17\|Trgv2\|Thy1\|Tlr2\|Klrk1\|Icos\|Ccrl2\|Klrc3\|Cd274\|Scara5\|Tnfrsf13c\|Muc16\|Slamf7\|Klrb1b\|Clec2h\|Trgc2\|Tlr8\|Nlgn1\|Ighv9-1\|Ighv1-78\|Cd226\|Tnfrsf14\|Clec2e\|Ighv14-3\|Ighg3\|Ighv10-3\|Ighv1-64\|Ighv1-66\|H2-Eb2\|Ighv1-72\|Ighv9-2\|Ighv14-4\|Ighv1-12\|Ighv1-26\|Ighv5-16\|Ighv9-4\|Ighv14-2\|Ighv3-3\|Ighv1-7\|Ighv1-85\|H60c\|Ighv2-2\|Ighv5-17\|Ighv9-3\|Ighv1-39\|Ighv1-53\|Ighv8-8\|Ighv1-58\|Ighv1-63\|Ighv8-12\|Ccl21b |
| GO Cellular Components | GO:0070469 | respirasome | -11 | 5.1 | 26 | Cox6a2\|Cox7a1\|COX1\|COX2\|COX3\|CYTB\|ND1\|ND2\|ND3\|ND4\|ND5\|ND6\|Ndufa2\|Uqcrq\|Ndufa1\|Ndufa3\|Uqcr10\|Ndufc1\|Ndufb3\|Uqcrh\|Uqcr11\|Ndufa5\|Ndufb10\|Ndufb6\|Ndufs6\|Wdr93 |
| GO Cellular Components | GO:0098803 | respiratory chain complex | -10 | 5 | 23 | Cox6a2\|COX1\|COX2\|COX3\|CYTB\|ND1\|ND2\|ND3\|ND4\|ND5\|Ndufa2\|Uqcrq\|Ndufa1\|Ndufa3\|Uqcr10\|Ndufc1\|Ndufb3\|Uqcrh\|Ndufa5\|Ndufb10\|Ndufb6\|Ndufs6\|Wdr93 |
| GO Cellular Components | GO:1990204 | oxidoreductase complex | -10 | 4.3 | 27 | Cybb\|Gpd1\|COX1\|CYTB\|ND1\|ND2\|ND3\|ND4\|ND5\|Ndufa2\|Pdk2\|Rrm2\|Uqcrq\|Ndufa1\|Ndufa3\|Uqcr10\|Gmpr\|Ndufc1\|Ndufb3\|Uqcrh\|Ndufa5\|Ndufb10\|Etfb\|Ndufb6\|Noxa1\|Ndufs6\|Wdr93 |
| GO Cellular Components | GO:0098800 | inner mitochondrial membrane protein complex | -10 | 4 | 29 | Atp5g1\|Atp5k\|Cox6a2\|ATP6\|ATP8\|COX1\|CYTB\|ND1\|ND2\|ND3\|ND4\|ND5\|Ndufa2\|Uqcrq\|Ndufa1\|Ndufa3\|Uqcr10\|Ndufc1\|Ndufb3\|Uqcrh\|Romo1\|Atp5e\|Ndufa5\|Ndufb10\|Chchd10\|Atp5g3\|Ndufb6\|Ndufs6\|Wdr93 |
| GO Cellular Components | GO:0005746 | mitochondrial respirasome | -9.9 | 5.1 | 22 | Cox6a2\|Cox7a1\|COX1\|CYTB\|ND1\|ND2\|ND3\|ND4\|ND5\|Ndufa2\|Uqcrq\|Ndufa1\|Ndufa3\|Uqcr10\|Ndufc1\|Ndufb3\|Uqcrh\|Ndufa5\|Ndufb10\|Ndufb6\|Ndufs6\|Wdr93 |
| GO Cellular Components | GO:0042571 | immunoglobulin complex, circulating | -9.2 | 3.7 | 29 | Ighg2b\|Ighg1\|Ighv9-1\|Ighv1-78\|Ighv14-3\|Ighg3\|Ighv10-3\|Ighv1-64\|Ighv1-66\|Ighv1-72\|Ighv9-2\|Ighv14-4\|Ighv1-12\|Ighv1-26\|Ighv5-16\|Ighv9-4\|Ighv14-2\|Ighv3-3\|Ighv1-7\|Ighv1-85\|Ighv2-2\|Ighv5-17\|Ighv9-3\|Ighv1-39\|Ighv1-53\|Ighv8-8\|Ighv1-58\|Ighv1-63\|Ighv8-12 |
| GO Cellular Components | GO:0019814 | immunoglobulin complex | -9.1 | 3.6 | 30 | Ighg2b\|Ighg1\|Iglv2\|Ighv9-1\|Ighv1-78\|Ighv14-3\|Ighg3\|Ighv10-3\|Ighv1-64\|Ighv1-66\|Ighv1-72\|Ighv9-2\|Ighv14-4\|Ighv1-12\|Ighv1-26\|Ighv5-16\|Ighv9-4\|Ighv14-2\|Ighv3-3\|Ighv1-7\|Ighv1-85\|Ighv2-2\|Ighv5-17\|Ighv9-3\|Ighv1-39\|Ighv1-53\|Ighv8-8\|Ighv1-58\|Ighv1-63\|Ighv8-12 |
| GO Cellular Components | GO:0045271 | respiratory chain complex I | -7.4 | 5.5 | 15 | ND1\|ND2\|ND3\|ND4\|ND5\|Ndufa2\|Ndufa1\|Ndufa3\|Ndufc1\|Ndufb3\|Ndufa5\|Ndufb10\|Ndufb6\|Ndufs6\|Wdr93 |
| GO Cellular Components | GO:0030964 | NADH dehydrogenase complex | -7.4 | 5.5 | 15 | ND1\|ND2\|ND3\|ND4\|ND5\|Ndufa2\|Ndufa1\|Ndufa3\|Ndufc1\|Ndufb3\|Ndufa5\|Ndufb10\|Ndufb6\|Ndufs6\|Wdr93 |
| GO Cellular Components | GO:0005747 | mitochondrial respiratory chain complex I | -7.4 | 5.5 | 15 | ND1\|ND2\|ND3\|ND4\|ND5\|Ndufa2\|Ndufa1\|Ndufa3\|Ndufc1\|Ndufb3\|Ndufa5\|Ndufb10\|Ndufb6\|Ndufs6\|Wdr93 |
| GO Cellular Components | GO:0001772 | immunological synapse | -6.9 | 5.5 | 14 | Cd28\|Cd3e\|Cd53\|Cd6\|Icam1\|Itgal\|Lck\|Myh9\|Crtam\|Skap1\|Card11\|Havcr2\|Scimp\|H2-Eb2 |
| GO Cellular Components | GO:0098798 | mitochondrial protein-containing complex | -6.2 | 2.5 | 36 | Slc25a4\|Atp5g1\|Atp5k\|Cox6a2\|ATP6\|ATP8\|COX1\|CYTB\|ND1\|ND2\|ND3\|ND4\|ND5\|mt-Rnr1\|Ndufa2\|Pdk2\|Uqcrq\|Timm13\|Ndufa1\|Ndufa3\|Uqcr10\|Tomm7\|Ndufc1\|Ndufb3\|Uqcrh\|Romo1\|Atp5e\|Ndufa5\|Ndufb10\|Mrpl14\|Chchd10\|Etfb\|Atp5g3\|Ndufb6\|Ndufs6\|Wdr93 |
| GO Cellular Components | GO:0005743 | mitochondrial inner membrane | -5.4 | 2 | 50 | Slc25a4\|Atp5g1\|Atp5k\|Bcl2l1\|Clu\|Cox6a2\|Cox7a1\|Cyp1a1\|Cyp2e1\|Endog\|ATP6\|ATP8\|COX1\|COX2\|COX3\|CYTB\|ND1\|ND2\|ND3\|ND4\|ND5\|ND6\|Ndufa2\|Ucp3\|Uqcrq\|Timm13\|Ndufa1\|Ndufa3\|Uqcr10\|Ndufc1\|Ndufb3\|Uqcrh\|Uqcr11\|Romo1\|Gatm\|Atp5e\|Mtln\|Ndufa5\|Ndufb10\|Tmem256\|Slc25a33\|Rcc1l\|Chchd10\|Atp5g3\|Ndufb6\|Slc25a48\|Slc25a34\|Ndufs6\|Wdr93\|Pet100 |
| GO Cellular Components | GO:0019866 | organelle inner membrane | -4.6 | 1.8 | 51 | Slc25a4\|Atp5g1\|Atp5k\|Bcl2l1\|Clu\|Cox6a2\|Cox7a1\|Cyp1a1\|Cyp2e1\|Endog\|Lmnb1\|ATP6\|ATP8\|COX1\|COX2\|COX3\|CYTB\|ND1\|ND2\|ND3\|ND4\|ND5\|ND6\|Ndufa2\|Ucp3\|Uqcrq\|Timm13\|Ndufa1\|Ndufa3\|Uqcr10\|Ndufc1\|Ndufb3\|Uqcrh\|Uqcr11\|Romo1\|Gatm\|Atp5e\|Mtln\|Ndufa5\|Ndufb10\|Tmem256\|Slc25a33\|Rcc1l\|Chchd10\|Atp5g3\|Ndufb6\|Slc25a48\|Slc25a34\|Ndufs6\|Wdr93\|Pet100 |
| GO Cellular Components | GO:1990351 | transporter complex | -4.1 | 1.9 | 40 | Chrna7\|Atp5g1\|Atp10a\|Cacna1a\|Cacna1d\|Cacnb1\|Cldn4\|Gabra3\|Grik5\|COX1\|CYTB\|ND1\|ND2\|ND3\|ND4\|ND5\|Ndufa2\|Pde4b\|Scn1b\|Uqcrq\|Timm13\|Ndufa1\|Trpv6\|Ndufa3\|Uqcr10\|Ndufc1\|Ndufb3\|Uqcrh\|Ndufa5\|Ndufb10\|Kcnip4\|Sacm1l\|Atp1a2\|Lrrc26\|Atp5g3\|Ndufb6\|Cngb1\|Shisa6\|Ndufs6\|Wdr93 |
| GO Cellular Components | GO:0005740 | mitochondrial envelope | -3.8 | 1.6 | 62 | Slc25a4\|Atp5g1\|Atp5k\|Bcl2l1\|Bik\|Cidea\|Clu\|Cox6a2\|Cox7a1\|Cyp1a1\|Cyp2e1\|Endog\|ATP6\|ATP8\|COX1\|COX2\|COX3\|CYTB\|ND1\|ND2\|ND3\|ND4\|ND5\|ND6\|Mt3\|Ndufa2\|Ucp3\|Uqcrq\|Timm13\|Ndufa1\|Ndufa3\|Uqcr10\|Tomm7\|Ndufc1\|Ndufb3\|Uqcrh\|Uqcr11\|Romo1\|Gatm\|Atp5e\|Mtln\|Ndufa5\|Ndufb10\|Tmem256\|Vrk2\|Slc25a33\|Ggnbp1\|Sting1\|Trim14\|Cstad\|Rcc1l\|Chchd10\|Adap2\|Atp5g3\|Ndufb6\|Mief2\|Vps13c\|Slc25a48\|Slc25a34\|Ndufs6\|Wdr93\|Pet100 |
| GO Cellular Components | GO:1902495 | transmembrane transporter complex | -3.8 | 1.9 | 38 | Chrna7\|Atp5g1\|Cacna1a\|Cacna1d\|Cacnb1\|Cldn4\|Gabra3\|Grik5\|COX1\|CYTB\|ND1\|ND2\|ND3\|ND4\|ND5\|Ndufa2\|Pde4b\|Scn1b\|Uqcrq\|Ndufa1\|Trpv6\|Ndufa3\|Uqcr10\|Ndufc1\|Ndufb3\|Uqcrh\|Ndufa5\|Ndufb10\|Kcnip4\|Sacm1l\|Atp1a2\|Lrrc26\|Atp5g3\|Ndufb6\|Cngb1\|Shisa6\|Ndufs6\|Wdr93 |
| GO Cellular Components | GO:0031966 | mitochondrial membrane | -3.7 | 1.6 | 58 | Slc25a4\|Atp5g1\|Atp5k\|Bcl2l1\|Clu\|Cox6a2\|Cox7a1\|Cyp1a1\|Cyp2e1\|Endog\|ATP6\|ATP8\|COX1\|COX2\|COX3\|CYTB\|ND1\|ND2\|ND3\|ND4\|ND5\|ND6\|Mt3\|Ndufa2\|Ucp3\|Uqcrq\|Timm13\|Ndufa1\|Ndufa3\|Uqcr10\|Tomm7\|Ndufc1\|Ndufb3\|Uqcrh\|Uqcr11\|Romo1\|Gatm\|Atp5e\|Mtln\|Ndufa5\|Ndufb10\|Tmem256\|Vrk2\|Slc25a33\|Sting1\|Trim14\|Cstad\|Rcc1l\|Chchd10\|Atp5g3\|Ndufb6\|Mief2\|Vps13c\|Slc25a48\|Slc25a34\|Ndufs6\|Wdr93\|Pet100 |
| GO Cellular Components | GO:0005750 | mitochondrial respiratory chain complex III | -3.7 | 8.1 | 5 | COX1\|CYTB\|Uqcrq\|Uqcr10\|Uqcrh |
| GO Molecular Functions | GO:0140375 | immune receptor activity | -15 | 4.9 | 37 | Cxcr5\|C3ar1\|C5ar1\|Ccr6\|Cd4\|Cxcr2\|Cxcr3\|Ccr1\|Ccr9\|Ccr2\|Ccr8\|Csf2rb\|Csf2rb2\|Csf3r\|Ctsh\|Fcer1g\|Fcgr2b\|Fcgr3\|Ifnar2\|Cd74\|Il12b\|Il12rb1\|Il12rb2\|Il13ra2\|Il2ra\|Il2rb\|Il2rg\|Il3ra\|Klrc1\|Klrc2\|Klrk1\|Ccrl2\|Klrc3\|Il21r\|Il22ra2\|C5ar2\|H2-Eb2 |
| GO Molecular Functions | GO:0003823 | antigen binding | -12 | 3.3 | 43 | Cd48\|Fcnb\|H2-Bl\|H2-K1\|H2-Q1\|H2-T24\|Ighg2b\|Ighg1\|Itga4\|Klrc1\|Klrc2\|Lck\|Klrc3\|Dhcr24\|Spon2\|Ighv9-1\|Ighv1-78\|Ighv14-3\|Ighg3\|Ighv10-3\|Ighv1-64\|Ighv1-66\|H2-Eb2\|Ighv1-72\|Ighv9-2\|Ighv14-4\|Ighv1-12\|Ighv1-26\|Ighv5-16\|Ighv9-4\|Ighv14-2\|Ighv3-3\|Ighv1-7\|Ighv1-85\|Ighv2-2\|Ighv5-17\|Ighv9-3\|Ighv1-39\|Ighv1-53\|Ighv8-8\|Ighv1-58\|Ighv1-63\|Ighv8-12 |
| GO Molecular Functions | GO:0019955 | cytokine binding | -11 | 4 | 31 | Cxcr5\|Ccr6\|Cd4\|Cxcr2\|Cxcr3\|Ccr1\|Ccr9\|Ccr2\|Ccr8\|Csf3r\|Gbp2\|Ifnar2\|Cd74\|Il12b\|Il12rb1\|Il12rb2\|Il13ra2\|Il1rn\|Il2ra\|Il2rb\|Il2rg\|Il3ra\|Itga4\|Tcap\|Elane\|Ccrl2\|Tnfrsf14\|A2m\|Cd109\|Il22ra2\|Tsku |
| GO Molecular Functions | GO:0004896 | cytokine receptor activity | -10 | 4.6 | 25 | Cxcr5\|Ccr6\|Cd4\|Cxcr2\|Cxcr3\|Ccr1\|Ccr9\|Ccr2\|Ccr8\|Csf2rb\|Csf2rb2\|Csf3r\|Ifnar2\|Cd74\|Il12b\|Il12rb1\|Il12rb2\|Il13ra2\|Il2ra\|Il2rb\|Il2rg\|Il3ra\|Ccrl2\|Il21r\|Il22ra2 |
| GO Molecular Functions | GO:0015453 | oxidoreduction-driven active transmembrane transporter activity | -9.1 | 6.1 | 17 | Cox6a2\|Cox7a1\|COX1\|COX2\|COX3\|CYTB\|ND1\|ND2\|ND3\|ND4\|ND5\|ND6\|Ndufa2\|Uqcrq\|Uqcrh\|Uqcr11\|Cyb561a3 |
| GO Molecular Functions | GO:0034987 | immunoglobulin receptor binding | -9.1 | 3.7 | 29 | Ighg2b\|Ighg1\|Ighv9-1\|Ighv1-78\|Ighv14-3\|Ighg3\|Ighv10-3\|Ighv1-64\|Ighv1-66\|Ighv1-72\|Ighv9-2\|Ighv14-4\|Ighv1-12\|Ighv1-26\|Ighv5-16\|Ighv9-4\|Ighv14-2\|Ighv3-3\|Ighv1-7\|Ighv1-85\|Ighv2-2\|Ighv5-17\|Ighv9-3\|Ighv1-39\|Ighv1-53\|Ighv8-8\|Ighv1-58\|Ighv1-63\|Ighv8-12 |
| GO Molecular Functions | GO:0009055 | electron transfer activity | -7.4 | 4.6 | 18 | Cox6a2\|Cox7a1\|Cybb\|COX1\|COX2\|COX3\|CYTB\|ND1\|ND2\|ND3\|ND4\|ND5\|ND6\|Ndufa2\|Uqcrq\|Uqcrh\|Uqcr11\|Etfb |
| GO Molecular Functions | GO:0019956 | chemokine binding | -5.9 | 5.8 | 11 | Cxcr5\|Ccr6\|Cxcr2\|Cxcr3\|Ccr1\|Ccr9\|Ccr2\|Ccr8\|Itga4\|Ccrl2\|A2m |
| GO Molecular Functions | GO:0038187 | pattern recognition receptor activity | -5.7 | 6.2 | 10 | Fcnb\|Ptafr\|Tlr2\|Clec4e\|Clec7a\|Pglyrp2\|Tlr9\|Tlr7\|Tlr8\|Trim30d |
| GO Molecular Functions | GO:0015078 | proton transmembrane transporter activity | -5.5 | 3.2 | 20 | Slc25a4\|Atp5g1\|Atp5k\|Cox6a2\|Cox7a1\|ATP6\|ATP8\|COX1\|COX2\|COX3\|CYTB\|Slc11a1\|Uqcrq\|Uqcrh\|Uqcr11\|Atp5e\|Mfsd3\|Atp6v1b1\|Slc9a2\|Atp5g3 |
| GO Molecular Functions | GO:0016493 | C-C chemokine receptor activity | -5.5 | 6.7 | 9 | Cxcr5\|Ccr6\|Cxcr2\|Cxcr3\|Ccr1\|Ccr9\|Ccr2\|Ccr8\|Ccrl2 |
| GO Molecular Functions | GO:0001637 | G protein-coupled chemoattractant receptor activity | -5.3 | 6.4 | 9 | Cxcr5\|Ccr6\|Cxcr2\|Cxcr3\|Ccr1\|Ccr9\|Ccr2\|Ccr8\|Ccrl2 |
| GO Molecular Functions | GO:0004950 | chemokine receptor activity | -5.3 | 6.4 | 9 | Cxcr5\|Ccr6\|Cxcr2\|Cxcr3\|Ccr1\|Ccr9\|Ccr2\|Ccr8\|Ccrl2 |
| GO Molecular Functions | GO:0019957 | C-C chemokine binding | -5.3 | 6.4 | 9 | Cxcr5\|Ccr6\|Cxcr2\|Cxcr3\|Ccr1\|Ccr9\|Ccr2\|Ccr8\|Ccrl2 |
| GO Molecular Functions | GO:0045028 | G protein-coupled purinergic nucleotide receptor activity | -4.7 | 9 | 6 | Ptafr\|P2ry13\|P2ry10\|Gpr87\|P2ry14\|Gpr171 |
| GO Molecular Functions | GO:0023023 | MHC protein complex binding | -4.7 | 6.2 | 8 | B2m\|Cd4\|Cd8a\|Cd74\|Klrc1\|Klrc2\|Klrc3\|H2-Eb2 |
| GO Molecular Functions | GO:0016679 | oxidoreductase activity, acting on diphenols and related substances as donors | -4.6 | 11 | 5 | Cyp1a1\|CYTB\|Uqcrq\|Uqcrh\|Uqcr11 |
| GO Molecular Functions | GO:0003777 | microtubule motor activity | -4.2 | 3.5 | 13 | Kif11\|Kif4\|Kif20a\|Dnah10\|Kif18b\|Kif23\|Kif15\|Cenpe\|Kif20b\|Kif19a\|Dnah2\|Kif14\|Dnah7a |
| GO Molecular Functions | GO:0015399 | primary active transmembrane transporter activity | -4.2 | 2.4 | 23 | Atp7a\|Cox6a2\|Cox7a1\|Slc26a3\|COX1\|COX2\|COX3\|CYTB\|ND1\|ND2\|ND3\|ND4\|ND5\|ND6\|Ndufa2\|Uqcrq\|Slc26a4\|Uqcrh\|Uqcr11\|Atp5e\|Atp2c2\|Atp1a2\|Cyb561a3 |
| GO Molecular Functions | GO:0003774 | cytoskeletal motor activity | -4 | 2.8 | 17 | Kif11\|Kif4\|Myh9\|Myo1f\|Kif20a\|Dnah10\|Kif18b\|Kif23\|Kif15\|Cenpe\|Myo1h\|Kif20b\|Kif19a\|Dnah2\|Kif14\|Myo1a\|Dnah7a |
| KEGG Pathway | mmu05012 | Parkinson's disease | -10 | 3.9 | 31 | Slc25a4\|Atp5g1\|Cox6a2\|Cox7a1\|ATP6\|ATP8\|COX1\|COX2\|COX3\|CYTB\|ND1\|ND2\|ND3\|ND4\|ND5\|ND6\|Ndufa2\|Uqcrq\|Ndufa1\|Ndufa3\|Uqcr10\|Ndufc1\|Ndufb3\|Uqcrh\|Uqcr11\|Atp5e\|Ndufa5\|Ndufb10\|Atp5g3\|Ndufb6\|Ndufs6 |
| KEGG Pathway | mmu04060 | Cytokine-cytokine receptor interaction | -10 | 3 | 44 | Cxcr5\|Ccr6\|Cxcr2\|Cxcr3\|Ccr1\|Ccr9\|Ccr2\|Ccr8\|Csf2rb\|Csf2rb2\|Csf3r\|Fasl\|Hgf\|Ifnar2\|Ifnb1\|Ifng\|Il12b\|Il12rb1\|Il12rb2\|Il1a\|Il1b\|Il2\|Il2ra\|Il2rb\|Il2rg\|Il3ra\|Il4\|Il6\|Ccl21a\|Ccl2\|Ccl3\|Ccl4\|Ccl5\|Cxcl2\|Tnfrsf8\|Vegfb\|Cxcl13\|Il21r\|Il21\|Tnfrsf13c\|Tnfrsf14\|Il22ra2\|Tnfsf15\|Ccl21b |
| KEGG Pathway | mmu05162 | Measles | -8.6 | 3.6 | 28 | Cd28\|Cd3e\|Cd3g\|Fasl\|Fcgr2b\|Hspa1b\|Ifnar2\|Ifnb1\|Ifng\|Il12b\|Il1a\|Il1b\|Il2\|Il2ra\|Il2rb\|Il2rg\|Il4\|Il6\|Myd88\|Tnfaip3\|Traf6\|Trp73\|Tlr2\|Irf7\|Tlr9\|Tlr7\|Hspa1a\|Oas3 |
| KEGG Pathway | mmu00190 | Oxidative phosphorylation | -8.3 | 3.1 | 33 | Atp5g1\|Atp5k\|Cox6a2\|Cox7a1\|ATP6\|ATP8\|COX1\|COX2\|COX3\|CYTB\|ND1\|ND2\|ND3\|ND4\|ND5\|ND6\|Ndufa2\|Uqcrq\|Ndufa1\|Ndufa3\|Uqcr10\|Ndufc1\|Ndufb3\|Uqcrh\|Uqcr11\|Atp5e\|Ppa1\|Ndufa5\|Ndufb10\|Atp6v1b1\|Atp5g3\|Ndufb6\|Ndufs6 |
| KEGG Pathway | mmu05016 | Huntington's disease | -7 | 2.9 | 31 | Slc25a4\|Atp5g1\|Cox6a2\|Cox7a1\|ATP6\|ATP8\|COX1\|COX2\|COX3\|CYTB\|Ndufa2\|Uqcrq\|Ndufa1\|Dnah10\|Ndufa3\|Uqcr10\|Ndufc1\|Polr2l\|Ndufb3\|Uqcrh\|Uqcr11\|Atp5e\|Ndufa5\|Ndufb10\|Dnai1\|Dnali1\|Atp5g3\|Ndufb6\|Dnah2\|Ndufs6\|Dnah7a |
| KEGG Pathway | mmu05321 | Inflammatory bowel disease (IBD) | -6.2 | 4.6 | 15 | Ifng\|Il12b\|Il12rb1\|Il12rb2\|Il1a\|Il1b\|Il2\|Il2rg\|Il4\|Il6\|Rorc\|Foxp3\|Tlr2\|Il21r\|Il21 |
| KEGG Pathway | mmu04640 | Hematopoietic cell lineage | -5.8 | 3.5 | 19 | Ms4a1\|Cd33\|Cd3e\|Cd3g\|Cd4\|Cd5\|Cd8a\|Cd8b1\|Csf3r\|Fcer2a\|Il1a\|Il1b\|Il2ra\|Il3ra\|Il4\|Il6\|Itga4\|Dntt\|Siglech |
| KEGG Pathway | mmu05010 | Alzheimer's disease | -5.8 | 2.8 | 27 | Apoe\|Atp5g1\|Cacna1d\|Cox6a2\|Cox7a1\|Il1b\|ATP6\|ATP8\|COX1\|COX2\|COX3\|CYTB\|Ndufa2\|Uqcrq\|Ndufa1\|Ndufa3\|Uqcr10\|Ndufc1\|Ndufb3\|Uqcrh\|Uqcr11\|Atp5e\|Ndufa5\|Ndufb10\|Atp5g3\|Ndufb6\|Ndufs6 |
| KEGG Pathway | mmu04932 | Non-alcoholic fatty liver disease (NAFLD) | -5.5 | 2.7 | 26 | Adipoq\|Cox6a2\|Cox7a1\|Cyp2e1\|Fasl\|Il1a\|Il1b\|Il6\|Irs1\|COX1\|COX2\|COX3\|CYTB\|Ndufa2\|Uqcrq\|Ndufa1\|Ndufa3\|Uqcr10\|Ndufc1\|Ndufb3\|Uqcrh\|Uqcr11\|Ndufa5\|Ndufb10\|Ndufb6\|Ndufs6 |
| KEGG Pathway | mmu04660 | T cell receptor signaling pathway | -5.3 | 3.2 | 19 | Cd28\|Cd3e\|Cd3g\|Cd4\|Cd8a\|Cd8b1\|Ifng\|Il2\|Il4\|Itk\|Lck\|Grap2\|Nfkbie\|Nr4a3\|Ptprc\|Icos\|Card11\|Raf1\|Pak6 |
| KEGG Pathway | mmu04514 | Cell adhesion molecules (CAMs) | -5 | 2.6 | 25 | Cd28\|Cd4\|Cd6\|Cd8a\|Cd8b1\|Cldn3\|Cldn4\|H2-Bl\|H2-K1\|H2-Q1\|H2-T24\|Icam1\|Itga4\|Itgal\|Itgb2\|Ptprc\|Selplg\|Siglec1\|Icos\|Cldn10\|Cd274\|Nlgn1\|Cd226\|Nrcam\|Tigit |
| KEGG Pathway | mmu04612 | Antigen processing and presentation | -4.9 | 3.3 | 17 | B2m\|Ciita\|Cd4\|Cd8a\|Cd8b1\|Hspa5\|H2-Bl\|H2-K1\|H2-Q1\|H2-T24\|Hspa1b\|Ifng\|Cd74\|Klrc1\|Klrc2\|Klrc3\|Hspa1a |
| KEGG Pathway | mmu04659 | Th17 cell differentiation | -4.9 | 3.2 | 18 | Cd3e\|Cd3g\|Cd4\|Ifng\|Il12rb1\|Il1b\|Il2\|Il2ra\|Il2rb\|Il2rg\|Il4\|Il6\|Lck\|Nfkbie\|Rorc\|Foxp3\|Il21r\|Il21 |
| KEGG Pathway | mmu05144 | Malaria | -4.8 | 4.3 | 12 | Hgf\|Icam1\|Ifng\|Il1b\|Il6\|Itgal\|Itgb2\|Myd88\|Ccl2\|Tlr2\|Tlr9\|Hbb-bt |
| KEGG Pathway | mmu05332 | Graft-versus-host disease | -4.8 | 3.7 | 14 | Cd28\|Fasl\|Gzmb\|H2-Bl\|H2-K1\|H2-Q1\|H2-T24\|Ifng\|Il1a\|Il1b\|Il2\|Il6\|Klrc1\|Prf1 |
| KEGG Pathway | mmu05340 | Primary immunodeficiency | -4.7 | 5 | 10 | Ciita\|Cd3e\|Cd4\|Cd8a\|Cd8b1\|Il2rg\|Lck\|Ptprc\|Icos\|Tnfrsf13c |
| KEGG Pathway | mmu05143 | African trypanosomiasis | -4.6 | 4.8 | 10 | Apoa1\|Fasl\|Icam1\|Ifng\|Il12b\|Il1b\|Il6\|Myd88\|Tlr9\|Hbb-bt |
| KEGG Pathway | mmu04621 | NOD-like receptor signaling pathway | -4.6 | 2.5 | 24 | Bcl2l1\|Camp\|Cybb\|Gbp2\|Ifi204\|Ifnar2\|Ifnb1\|Il1b\|Il6\|Myd88\|Naip2\|Ccl2\|Ccl5\|Cxcl2\|Tnfaip3\|Traf6\|Trpm2\|Irf7\|Mefv\|Ripk3\|Sting1\|Nlrp3\|Gbp5\|Oas3 |
| KEGG Pathway | mmu04650 | Natural killer cell mediated cytotoxicity | -4.5 | 2.8 | 20 | Cd48\|Fasl\|Fcer1g\|Gzmb\|H2-Bl\|H2-K1\|H2-Q1\|Icam1\|Ifnar2\|Ifnb1\|Ifng\|Itgal\|Itgb2\|Klrc1\|Klrc2\|Lck\|Prf1\|Rac2\|Klrk1\|Raf1 |
| KEGG Pathway | mmu05142 | Chagas disease (American trypanosomiasis) | -4.3 | 3 | 17 | Cd3e\|Cd3g\|Fasl\|Ifnb1\|Ifng\|Il12b\|Il1b\|Il2\|Il6\|Myd88\|Serpine1\|Ccl2\|Ccl3\|Ccl5\|Traf6\|Tlr2\|Tlr9 |
