# Supplementary material for: Exploring the immune-inflammatory mechanism of Maxing Shigan Decoction in treating influenza virus A-induced pneumonia based on an integrated strategy of single-cell transcriptomics and systems biology
Source: Eur J Med Res. 2024 Apr 15;29:234. doi: 10.1186/s40001-024-01777-9 (PMC11017673; doi:10.1186/s40001-024-01777-9)
Supplement: Supplementary file 5 — Additional file 5: Table S4. Top 20 different metabolites of model/blank comparison. [file 40001_2024_1777_MOESM5_ESM.docx]

Table S4 Top 20 different metabolites of model/blank comparison

| **Model** | **Annotation** | **Retention time (rt)** | **VIP** | **P-value** | **Log2FC** |
| --- | --- | --- | --- | --- | --- |
| POS model | Pipecolic acid | 295.1845 | 1.559038 | 0.02256 | -0.58139 |
|  | Phenylethylamine | 205.6045 | 2.18261 | 0.03146 | -5.20042 |
|  | Kynurenic acid | 194.26 | 1.595453 | 0.044728 | 0.98814 |
|  | 3-Formyl-6-hydroxyindole | 63.4403 | 1.818837 | 0.030135 | -2.11383 |
|  | Tyramine | 256.9645 | 2.624719 | 0.025537 | -4.00728 |
|  | Methylpyrazine | 466.701 | 1.906947 | 0.036024 | -1.86439 |
|  | Histamine | 466.8 | 1.539758 | 0.027438 | -1.40069 |
|  | Ethyl carbamate | 391.496 | 2.0178 | 0.04682 | -1.08341 |
|  | Phenylacetaldehyde | 257.029 | 2.594307 | 0.023264 | -3.83359 |
|  | 2-Methylbutyrylglycine | 342.719 | 1.727998 | 0.018207 | -0.89732 |
|  | Styrene | 205.563 | 2.161042 | 0.031563 | -5.14775 |
|  | 2,4-Dimethyl-1H-indole | 205.4965 | 2.126084 | 0.032018 | -5.18567 |
|  | 3-Methyl-1-butylamine | 232.918 | 1.838349 | 0.036084 | -3.46263 |
|  | Mulberrofuran E | 265.894 | 1.686626 | 0.009407 | -1.17797 |
|  | S-4-Hydroxymephenytoin | 69.884 | 1.847969 | 0.030986 | -0.6275 |
|  | N-Acetylornithine | 411.905 | 1.908858 | 0.021194 | -0.9805 |
|  | Tetracosahexaenoic acid | 58.94145 | 2.324949 | 0.017326 | -2.29756 |
|  | PC(22:6(4Z,7Z,10Z,13Z,16Z,19Z)/22:6(4Z,7Z,10Z,13Z,16Z,19Z)) | 157.642 | 1.32937 | 0.026374 | -1.46865 |
|  | L-Histidinol | 128.48 | 1.923247 | 0.006378 | -1.05715 |
|  | N-Palmitoylsphingosine | 34.2258 | 2.077204 | 0.020828 | 0.844434 |
| NEG model | Isolithocholic acid | 64.7734 | 2.096411 | 0.021025 | -1.86197 |
|  | Taurocholic acid | 269.5595 | 1.959909 | 0.02343 | 2.003604 |
|  | 2-Hydroxystearic acid | 53.25155 | 2.272855 | 0.041253 | -1.55103 |
|  | Chenodeoxycholic acid | 164.039 | 2.267271 | 0.000506 | -1.74829 |
|  | Pyroglutamic acid | 310.917 | 1.601207 | 0.042098 | -0.71884 |
|  | Uric acid | 335.242 | 1.847568 | 0.018973 | -0.9105 |
|  | Dodecanedioic acid | 244.6235 | 1.882238 | 0.0048 | -0.81032 |
|  | 4-Acetylbutyrate | 41.3821 | 1.858866 | 0.02405 | 1.361861 |
|  | 6-Methyladenine | 120.556 | 1.597821 | 0.049315 | -1.12526 |
|  | 9,10-DHOME | 74.4808 | 1.645986 | 0.021136 | -0.81489 |
|  | (10E,12Z)-(9S)-9-Hydroperoxyoctadeca-10,12-dienoic acid | 175.658 | 1.80192 | 0.018833 | -0.92851 |
|  | Xanthine | 225.75 | 1.447703 | 0.026687 | -0.98542 |
|  | Emodin | 121.5335 | 2.186727 | 0.013394 | 1.477934 |
|  | Pantothenic acid | 283.192 | 1.568006 | 0.030696 | -1.0279 |
|  | Prostaglandin B1 | 57.864 | 1.707056 | 0.024237 | -0.91679 |
|  | Enterodiol | 50.0962 | 1.417517 | 0.021375 | -0.73862 |
|  | Indole-3-propionic acid | 92.3476 | 1.524834 | 0.0119 | -1.20143 |
|  | 8-Isoprostaglandin F2a | 198.297 | 1.862703 | 0.006714 | -0.52622 |
|  | 3b-Hydroxy-5-cholenoic acid | 55.3378 | 1.916433 | 0.023887 | -1.1522 |
|  | N-Acetylmuramate | 302.51 | 1.553691 | 0.019888 | -1.16593 |
