# Supplementary material for: Exploring the immune-inflammatory mechanism of Maxing Shigan Decoction in treating influenza virus A-induced pneumonia based on an integrated strategy of single-cell transcriptomics and systems biology
Source: Eur J Med Res. 2024 Apr 15;29:234. doi: 10.1186/s40001-024-01777-9 (PMC11017673; doi:10.1186/s40001-024-01777-9)
Supplement: Supplementary file 6 — Additional file 6: Table S5. Top 20 different metabolites of MXSGD/model comparison. [file 40001_2024_1777_MOESM6_ESM.docx]

Table S5 Top 20 different metabolites of MXSGD/model comparison

| **Model** | **Annotation** | **Retention time (rt)** | **VIP** | **P-value** | **Log2FC** |
| --- | --- | --- | --- | --- | --- |
| POS model | Metenamine | 428.748 | 1.803146 | 0.021071 | -0.13053 |
|  | PE(20:3(8Z,11Z,14Z)/14:1(9Z)) | 171.6175 | 1.72945 | 0.035542 | -1.55212 |
|  | PE(20:5(5Z,8Z,11Z,14Z,17Z)/15:0) | 171.619 | 1.966276 | 0.016013 | -1.56922 |
|  | PE(20:4(5Z,8Z,11Z,14Z)/15:0) | 170.754 | 2.383409 | 0.017803 | -1.46898 |
|  | 25-Hydroxycholesterol | 34.40575 | 1.743646 | 0.032721 | -0.44203 |
|  | PC(22:6(4Z,7Z,10Z,13Z,16Z,19Z)/22:6(4Z,7Z,10Z,13Z,16Z,19Z)) | 157.642 | 1.678435 | 0.0376 | 0.76503 |
|  | 4-Hydroxy-5-phenyltetrahydro-1,3-oxazin-2-one | 38.65465 | 2.101559 | 0.037533 | -0.71993 |
|  | 4-Oxo-2-nonenal | 40.4749 | 1.736387 | 0.038732 | 0.808962 |
|  | (2alpha,3alpha,5alpha,22R,23R)-2,3,22,23-Tetrahydroxy-25-methylergost-24(28)en-6-one | 36.12915 | 1.913977 | 0.019667 | -0.458 |
|  | N1-Caffeoyl-N10-feruloylspermidine | 284.0625 | 1.339452 | 0.023082 | -1.06168 |
|  | 3',5'-Cyclic AMP | 145.4015 | 2.292171 | 0.004851 | -0.23103 |
|  | Cyhexatin | 146.376 | 2.490035 | 0.013846 | 1.352833 |
|  | Diphosphate | 17.8119 | 2.176635 | 0.015679 | -0.38199 |
|  | 1-(2-Hydroxyphenylamino)-1-deoxy-beta-D-gentiobioside 1,2-carbamate | 17.9276 | 1.940168 | 0.025177 | -0.69733 |
|  | 3-Carboxy-cis,cis-mucote | 19.9467 | 1.904875 | 0.031587 | -0.25941 |
|  | Dihydrokaempferol | 22.6439 | 1.681898 | 0.029974 | -0.26944 |
|  | 5-Amino-4-imidazolecarboxyamide | 23.65065 | 2.129709 | 0.005646 | -0.33137 |
|  | 6-({13,14-dihydroxy-9-oxo-8,17-dioxatetracyclo[8.7.0.0,.0,]heptadeca-1(10),2(7),3,5,11(16),12,14-heptaen-5-yl}oxy)-3,4,5-trihydroxyoxane-2-carboxylic acid | 34.3885 | 1.352599 | 0.040409 | -0.49923 |
|  | Caffeoyl aspartic acid | 35.61215 | 1.667614 | 0.0485 | -0.63841 |
|  | Clavulanic acid | 37.8585 | 2.257807 | 0.00276 | 0.855726 |
| NES model | Isolithocholic acid | 64.7734 | 2.481052 | 0.009535 | 1.679724 |
|  | Chenodeoxycholic acid | 164.039 | 2.906651 | 0.007957 | 1.523446 |
|  | Citramalic acid | 386.8315 | 2.775142 | 0.004416 | -0.75782 |
|  | 3b-Hydroxy-5-cholenoic acid | 55.3378 | 1.474586 | 0.034225 | 1.341832 |
|  | Mesaconic acid | 197.4235 | 2.589609 | 0.032215 | 0.89949 |
|  | 2-(3,4-dihydroxyphenyl)-3,5-dihydroxy-7-methoxy-4H-chromen-4-one | 161.6895 | 2.090589 | 0.042301 | -2.10119 |
|  | L-Cysteine | 158.1195 | 1.825748 | 0.041745 | 0.529806 |
|  | EDTA | 31.261 | 3.030019 | 0.027707 | 3.252789 |
|  | Metoxadiazone | 32.3124 | 2.732096 | 0.018475 | 3.360033 |
|  | Methylgallic acid-O-sulphate | 37.4839 | 3.012921 | 0.02483 | 2.61064 |
|  | Brevifolincarboxylic acid 9-sulfate | 41.3575 | 2.227885 | 0.024545 | 1.725454 |
|  | Prothiofos | 4.86581 | 2.371686 | 0.002162 | -2.78481 |
|  | dTDP-4-dimethylamino-4,6-dideoxy-5-C-methyl-L-mannose | 33.3178 | 2.197322 | 0.035021 | 2.076465 |
|  | XDP | 37.3578 | 2.130647 | 0.023382 | 1.720996 |
|  | 3'-(2',3'-Digalloyl-4',6'-hexahydroxydiphenoylglucosyl)-phloroacetophenone | 61.8845 | 1.844021 | 0.026324 | 2.674047 |
|  | (L-Seryl)adenylate | 65.1664 | 2.3887 | 0.011196 | 1.559267 |
|  | Cyanotriphenylborate | 95.4112 | 1.118934 | 0.049557 | -1.25706 |
|  | (2R)-O-Phospho-3-sulfolactate | 108.9755 | 2.635805 | 0.030548 | -1.54984 |
|  | 5-Phosphonooxy-L-lysine | 110.713 | 1.987676 | 0.044744 | 0.541874 |
|  | (R)-5-Phosphomevalote | 111.3885 | 2.144168 | 0.031636 | 0.905852 |
