# Supplementary material for: Exploring the immune-inflammatory mechanism of Maxing Shigan Decoction in treating influenza virus A-induced pneumonia based on an integrated strategy of single-cell transcriptomics and systems biology
Source: Eur J Med Res. 2024 Apr 15;29:234. doi: 10.1186/s40001-024-01777-9 (PMC11017673; doi:10.1186/s40001-024-01777-9)
Supplement: Supplementary file 7 — Additional file 7. Intestinal metabolite. [file 40001_2024_1777_MOESM7_ESM.docx]

**Additional file 7-Intestinal Metabolite**

**1 Metabolite Identification Results**

A total of 2321 compounds in Positive Ion Mode (POS) and 1325 compounds in Negative Ion Mode (NEG) were identified, including Organonitrogen compounds, Carboxylic acids and derivatives, Imidazopyrimidines, Fatty Acyls, Pyrrolotriazines, Tropane alkaloids, Dihydrofurans, Isoflavonoids, Azoles, Diazines, Pyridines and derivatives, Steroids and steroid derivatives, Purine nucleotides, Piperidines, Allyl sulfur compounds, etc (Figure S2). The metabolites in the stool sample are the products of co-metabolism between the intestinal microbiome and the host.


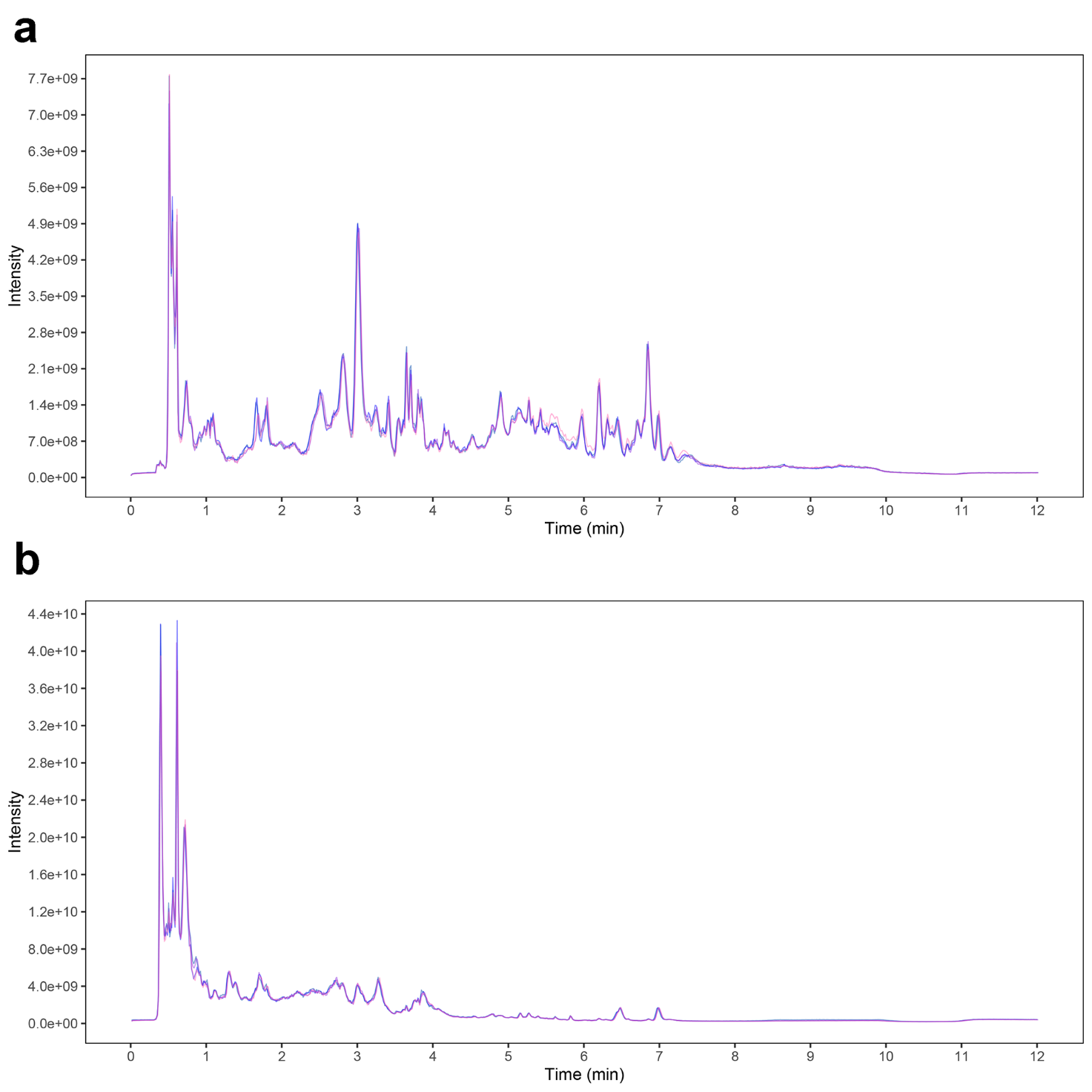


Figure S2 Total Ion Chromatography for UHPLC-QE-MS Detection of Fecal Samples (a: POS model; b: NEG model. The x-axis represents chromatographic peak retention time, and the y-axis represents chromatographic peak signal intensity)
